# Natural climate solutions for the United States

**DOI:** 10.1126/sciadv.aat1869

**Published:** 2018-11-14

**Authors:** Joseph E. Fargione, Steven Bassett, Timothy Boucher, Scott D. Bridgham, Richard T. Conant, Susan C. Cook-Patton, Peter W. Ellis, Alessandra Falcucci, James W. Fourqurean, Trisha Gopalakrishna, Huan Gu, Benjamin Henderson, Matthew D. Hurteau, Kevin D. Kroeger, Timm Kroeger, Tyler J. Lark, Sara M. Leavitt, Guy Lomax, Robert I. McDonald, J. Patrick Megonigal, Daniela A. Miteva, Curtis J. Richardson, Jonathan Sanderman, David Shoch, Seth A. Spawn, Joseph W. Veldman, Christopher A. Williams, Peter B. Woodbury, Chris Zganjar, Marci Baranski, Patricia Elias, Richard A. Houghton, Emily Landis, Emily McGlynn, William H. Schlesinger, Juha V. Siikamaki, Ariana E. Sutton-Grier, Bronson W. Griscom

**Affiliations:** 1 The Nature Conservancy, Minneapolis, MN 55415, USA.; 2 The Nature Conservancy, Santa Fe, NM 87501, USA.; 3 The Nature Conservancy, Arlington, VA 22203, USA.; 4 Institute of Ecology and Evolution, University of Oregon, Eugene, OR 97403, USA.; 5 Natural Resources Ecology Laboratory, Colorado State University, Fort Collins, CO 80523, USA.; 6 Smithsonian Environmental Research Center, Edgewater, MD 21037, USA.; 7 Food and Agriculture Organization, Rome, Italy.; 8 Marine Sciences Program, Florida International University, North Miami, FL 33181, USA.; 9 Graduate School of Geography, Clark University, Worcester, MA 01610, USA.; 10 Trade and Agriculture Directorate, Organization for Economic Cooperation and Development, Paris 75016, France.; 11 Department of Biology, University of New Mexico, Albuquerque, NM 87131, USA.; 12 Woods Hole Coastal and Marine Science Center, United States Geological Survey, Woods Hole, MA 02543, USA.; 13 Center for Sustainability and the Global Environment, University of Wisconsin-Madison, Madison, WI 53726, USA.; 14 The Nature Conservancy, Oxford OX1 1HU, UK.; 15 Department of Agricultural, Environmental and Development Economics, Ohio State University, Columbus, OH 43210, USA.; 16 Duke University Wetland Center, Nicholas School of the Environment, Durham, NC 27708, USA.; 17 Woods Hole Research Center, Falmouth, MA 02540, USA.; 18 TerraCarbon LLC, Charlottesville, VA 22903, USA.; 19 Department of Ecosystem Science and Management, Texas A&M University, College Station, TX 77843, USA.; 20 College of Agriculture and Life Sciences, Cornell University, Ithaca, NY 14853, USA.; 21 U.S. Department of Agriculture, Washington, DC 20250, USA.; 22 Department of Agriculture and Resource Economics, University of California, Davis, Davis, CA 95616, USA.; 23 Cary Institute of Ecosystem Studies, Millbrook, NY 12545, USA.; 24 International Union for Conservation of Nature, Washington, DC 20009, USA.; 25 The Nature Conservancy, Bethesda, MD 20814, USA.; 26 Earth System Science Interdisciplinary Center, University of Maryland, College Park, MD 20740, USA.

## Abstract

Limiting climate warming to <2°C requires increased mitigation efforts, including land stewardship, whose potential in the United States is poorly understood. We quantified the potential of natural climate solutions (NCS)—21 conservation, restoration, and improved land management interventions on natural and agricultural lands—to increase carbon storage and avoid greenhouse gas emissions in the United States. We found a maximum potential of 1.2 (0.9 to 1.6) Pg CO_2_e year^−1^, the equivalent of 21% of current net annual emissions of the United States. At current carbon market prices (USD 10 per Mg CO_2_e), 299 Tg CO_2_e year^−1^ could be achieved. NCS would also provide air and water filtration, flood control, soil health, wildlife habitat, and climate resilience benefits.

## Teaser

U.S. land management can contribute 1.2 Pg CO_2_e year^−1^ of greenhouse gas mitigation, 76% of which costs USD 50 Mg CO_2_e^−1^ or less.

## INTRODUCTION

Limiting global warming below the 2°C threshold set by the Paris Climate Agreement is contingent upon both reducing emissions and removing greenhouse gases (GHGs) from the atmosphere (*1*, *2*). Natural climate solutions (NCS), a portfolio of discrete land stewardship options (*3*), are the most mature approaches available for carbon conservation and uptake compared to nascent carbon capture technologies (*4*) and could complement increases in zero-carbon energy production and energy efficiency to achieve needed climate change mitigation. Within the United States, the maximum and economically viable mitigation potentials from NCS are unclear.

**Fig. 1:**
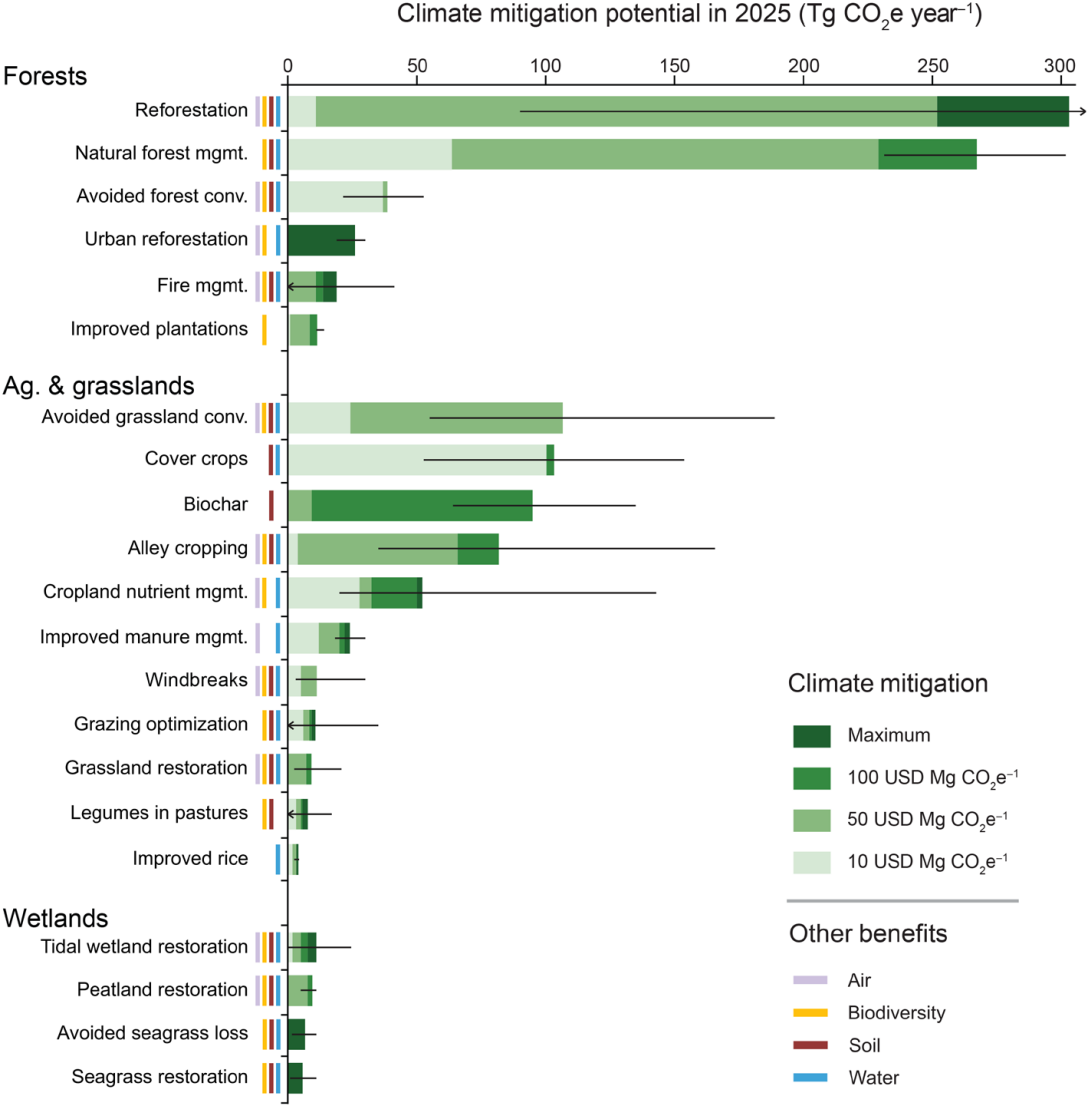
Climate mitigation potential of 21 NCS in the United States. Black lines indicate the 95% CI or reported range (see table S1). Ecosystem service benefits linked with each NCS are indicated by colored bars for air (filtration), biodiversity (habitat protection or restoration), soil (enrichment), and water (filtration and flood control). See the Supplementary Materials for detailed findings and sources.

Here, we quantify the maximum potential for NCS in the United States and the portion of this maximum that could be achieved at several price points. We consider 21 distinct NCS to provide a consistent and comprehensive exploration of the mitigation potential of conservation, restoration, and improved management in forests, grasslands, agricultural lands, and wetlands (Fig. 1), carefully defined to avoid double counting (details in the Supplementary Materials). We estimate the potential for NCS in the year 2025, which is the target year for the United States’ Nationally Determined Contribution (NDC) under the Paris Agreement to reduce GHG emissions by 26 to 28% from 2005 levels. Our work refines a coarser-resolution global analysis (*3*) and updates and expands the range of options considered in previous analyses for the United States (*5*–*8*).

For each NCS opportunity (Fig. 1 and the Supplementary Materials), we estimate the maximum mitigation potential of GHGs measured in CO_2_ equivalents (CO_2_e), given the below constraints. We then estimate the reductions obtainable for less than USD 10, 50, and 100 per Mg CO_2_e. Current carbon markets pay around USD 10 (*9*). The social cost of carbon in 2025 is approximately USD 50, using a 3% discount rate (*10*). However, a price of at least USD 100 is thought to be needed to keep the 100-year average temperature from warming more than 2.5°C (*11*), and an even higher price may be needed to meet the Paris Agreement <2°C target. Many NCS also generate co-benefits, which, even without a price on carbon, provide incentives to invest in NCS implementation. We identified co-benefits generated by each NCS in four categories of ecosystem services: air, biodiversity, water, and soil (Fig. 1 and table S2).

To avoid conflicts with other important societal goals for land use, we constrain our maximum estimate to be compatible with human needs for food and fiber (Supplementary Materials). Within these constraints, 5.1 Mha of cropland can be restored to grasslands, forests, and wetlands, equal to the area that has left the Conservation Reserve Program (CRP) since 2007 (*8*) and less than half the land currently dedicated to corn ethanol. We also estimate that 1.3 Mha of pasture could be reforested without affecting livestock production, assuming recent improvements in efficiency continue (see the Supplementary Materials). We assume that timber production can temporarily decrease by 10%, which maintains timber production levels within the historic range of variation and enables managed forests and plantations to transition to longer harvest rotations (see the Supplementary Materials). We assume that extensive natural forests on private lands can all undergo harvest extension, with the temporary loss of timber supply replaced by reforestation and thinning for fire risk reduction (*12*) or with thinning or select harvest practices that still provide timber but maintain carbon levels (Supplementary Materials) (*13*, *14*). We further constrain our analysis to avoid impacts on biodiversity. This biodiversity constraint precludes both the conversion of natural habitat to energy crops and the afforestation of native grasslands.

## RESULTS

We find a maximum additional NCS mitigation potential of 1.2 Pg CO_2_e year^−1^ [95% confidence interval (CI), 0.9 to 1.6 Pg CO_2_e year^−1^] in the year 2025 (Fig. 1 and table S1). This is 21% of the 5794.5 Tg CO_2_e of net emissions in 2016 (*15*). The majority (63%) of this potential comes from increased carbon sequestration in plant biomass, with 29% coming from increased carbon sequestration in soil and 7% coming from avoided emissions of CH_4_ and N_2_O. At the USD 10, 50, and 100 price points, 25, 76, and 91%, respectively, of the maximum mitigation would be achieved. This means that 1.1 Pg CO_2_e year^−1^ are available at USD 100 per Mg CO_2_e, which equals the emission reductions needed to meet the U.S. NDC under the Paris Agreement (see the Supplementary Materials). If NCS were pursued in combination with additional mitigation in the energy sector, then it would therefore enable the United States to exceed its current NDC ambition. This is important because, globally, current NDCs (7 to 9 Pg CO_2_e year^−1^) would need to be dramatically increased (by an additional 10 to 16 Pg CO_2_e year^−1^) to limit warming below 2°C (*16*).

This estimate of maximum NCS potential is similar to or higher than several previous syntheses of mitigation opportunities in the land sector. For example, the United States Mid-Century Strategy for Deep Decarbonization estimated a potential land sink of 912 Tg CO_2_e year^−1^, 30% lower than our estimate (*5*). While other efforts have focused on the forest sector (*7*) or the agricultural sector (*6*), this analysis presents a comprehensive and up-to-date synthesis of NCS opportunities in the United States. For example, this analysis considers potential additional mitigation from tidal wetlands and seagrass (“blue carbon”), which has been comprehensively analyzed for its current status in the United States (*17*), but not its potential for additional mitigation.

Reforestation has the single largest maximum mitigation potential (307 Tg CO_2_e year^−1^). The majority of this potential occurs in the northeast (35%) and south central (31%) areas of the United States (fig. S1). This mitigation potential increases to 381 Tg CO_2_e year^−1^ if all pastures in historically forested areas are reforested. Previous estimates of reforestation potential range widely from 208 to 1290 Tg CO_2_e year^−1^ (*7*). Higher estimates than ours can be obtained by reforesting or afforesting areas that we excluded (e.g., productive crop and pasture lands and natural grasslands) and/or by using rates of carbon sequestration from plantation systems rather than from natural regenerating forests [e.g., (*7*)].

Natural forest management of privately held forests has the second largest maximum mitigation potential (267 Tg CO_2_e year^−1^). This maximum mitigation is achieved by extending harvest cycles. Mitigation can also be achieved through forest management practices such as reduced impact logging and improved silvicultural practices that release suppressed forest growth (*18*–*20*), although often at lower sequestration rates than extending harvest cycles. These management practices can be implemented at low or no net cost (*21*, *22*) and do not require a change in business-as-usual (BAU) land use or ownership rights.

Another promising opportunity associated with forests is fire management (18 Tg CO_2_e year^−1^; fig. S6). Fire management entails restoring frequent, low-intensity, understory fires in fire-prone forest ecosystems to reduce the potential for high-severity wildfires (*23*). The primary carbon benefit from fire management is avoiding decreased net ecosystem production from tree-killing wildfire. In the absence of improved fire management, climate change is expected to continue to increase the frequency of high-severity fires and compromise the ability of forests to regenerate following these fires (*24*). The high uncertainty associated with the climate mitigation benefits of fire management would be reduced by additional research to quantify the carbon storage benefits of prescribed fire across a diversity of forest types, including the length of time that prescribed fire reduces the risk of subsequent high-severity fires.

Avoided conversion protects carbon stored in extant forests and grasslands from ongoing losses. More than two-thirds of the avoided forest conversion potential (38 Tg CO_2_e year^−1^) occurs in the Southern and Pacific Northwest regions (table S14 and fig. S9). Many of the most intensive areas of rapid forest conversion were located near urban zones, with additional hot spots in recent agricultural expansion zones (such as California’s Central Valley) and semi-arid regions of the West. Avoided conversion of grassland to cropland prevents emissions from soils and root biomass (107 Tg CO_2_e year^−1^; fig. S12). The emissions from grassland conversion exceed the emissions from forest conversion because both the rate of conversion and the per hectare emissions are higher (table S1). Cropland expansion is a major cause of conversion that affects grasslands much more than forests (*25*). The higher rate of emissions occurs because the conversion of grasslands to croplands results in a 28% loss of soil carbon from the top meter of soil (*26*). This generates 125 Mg CO_2_e ha^−1^ in emissions, comprising 81% of the emissions from grassland conversion (see the Supplementary Materials). Because research shows conflicting conclusions regarding the impact of forest conversion in the United States on soil carbon, we do not include the soil carbon pool in our estimate of emissions from forest conversion (see the Supplementary Materials).

Carbon sequestration opportunities in croplands include the use of cover crops and improved cropland nutrient management. Cover crops, grown when fields are normally bare, provide additional carbon inputs to soils. Growing cover crops on the 88 Mha of the five primary crops in United States not already using cover crops presents a substantial opportunity for mitigation (103 Tg CO_2_e year^−1^). Cover crops are increasingly used by U.S. farmers to improve soil health, yields, and yield consistency (*27*). Improved management of nitrogen fertilizers reduces N_2_O emissions and avoids fossil fuel emissions associated with fertilizer production (52 Tg CO_2_e year^−1^). Fertilizer rates can be reduced while maintaining yields by using precision agriculture to apply only the amount required in each part of the field and by splitting fertilizer applications to match the timing and supply of fertilizer with crop demand (see the Supplementary Materials). Emissions can also be reduced by switching from anhydrous fertilizer to urea, which has lower N_2_O emission (*6*).

The agronomic practices of biochar incorporation (95 Tg CO_2_e year^−1^) and alley cropping (planting widely spaced trees interspersed with a row crop; 82 Tg CO_2_e year^−1^) also have high maximum potential. However, current adoption is negligible due to a variety of cultural, technological, and cost barriers that would need to be overcome if these practices were to achieve their mitigation potential (*28*, *29*).

Tidal wetland restoration is the largest wetland NCS (12 Tg CO_2_e year^−1^). Roughly 27% of U.S. salt marshes are disconnected from the ocean and subject to freshwater inundation. This results in a large increase in CH_4_ emissions from these “freshened” salt marshes. Reconnecting salt marshes with the ocean, such as via culverts under roads or other barriers, can avoid these CH_4_ emissions (*30*).

The 10 opportunities described above account for 90% (1082 Tg CO_2_e year^−1^) of the maximum NCS mitigation potential across all 21 opportunities. An additional 11 opportunities, which sum to 122 Tg CO_2_e year^−1^, account for just 10% of the maximum potential. However, these NCS may offer optimal ecological and economic opportunities at local scales (Fig. 1 and Supplementary Materials). For example, peatland restoration offers a high per hectare mitigation benefit, especially in regions of the United States with warm temperate climates (8.2 Mg CO_2_e ha^−1^ year^−1^).

Lower-cost opportunities represent particularly promising areas for increased near-term investment. We identified 299 Tg CO_2_e year^−1^ of NCS opportunities that could be realized for USD 10 Mg CO_2_e^−1^ or less (table S1), a price that is in line with many current carbon markets (*9*). The two largest lower-cost opportunities are improved management practices: cover crops (100 Tg CO_2_e year^−1^) and improved natural forest management (64 Tg CO_2_e year^−1^). Both of these practices, along with planting windbreaks (5 Tg CO_2_e year^−1^) and legumes in pastures (3 Tg CO_2_e year^−1^), have the potential to increase yields (*21*, *22*, *27*) and therefore to generate additional revenue for landowners. Improved manure management can also provide low-cost mitigation (12 Tg CO_2_e year^−1^) (*8*). In addition, lower-cost NCS include increased efficiencies (cropland nutrient management, 28 Tg CO_2_e year^−1^; grazing optimization, 6 Tg CO_2_e year^−1^) and avoided conversion (avoided forest conversion, 37 Tg CO_2_e year^−1^; avoided grassland conversion, 24 Tg CO_2_e year^−1^).

By itself, the marginal abatement cost gives an incomplete picture of the potential for implementation of NCS, in part because NCS provide a variety of co-benefits (Fig. 1 and table S2). The values of these co-benefits are not captured in our marginal abatement costs yet may drive NCS implementation. For example, investments in fire management are needed to avoid impacts on air quality and drinking water provision; urban forestry provides human health, aesthetic, and direct temperature reduction benefits; nutrient management is needed to improve water quality and avoid toxic algal blooms (table S2). Further, NCS can help provide resilience to climate change impacts on nature and people. For example, building soil carbon increases the resilience of cropland (*31*); protecting coastal wetlands can provide coastal defense against storms (*32*); and fire management can help avoid damaging wildfires (*23*).

We have restricted our analysis to those opportunities where the literature conclusively demonstrates the potential for mitigation. This suggests that new research may reveal additional opportunities for NCS, which would increase the potential identified here. At the same time, substantial uncertainties exist in some NCS opportunities (Fig. 1 and table S1), highlighting the need for implementation to be coupled with monitoring and assessment of NCS.

## DISCUSSION

The United States is the largest cumulative emitter of carbon dioxide from fossil fuels (*33*). Despite the immense size of U.S. GHG emissions from fossil fuel use, we find that NCS have the potential to generate mitigation equivalent to 21% of net annual emissions. This reveals the important contribution to climate mitigation that the land sector can make, even in developed countries such as the United States.

Globally, current NCS efforts receive only 0.8% of public and private climate financing (*34*), despite offering roughly 37% of potential mitigation needed through 2030 (*3*). One concern that may have limited the adoption of NCS to date includes competition with other land uses such as food and bioenergy production. A growing body of literature suggests that future global food demand can be met via investments in yield increases, closing yield gaps, diet shifts, aquaculture, and biofuel policy, without the need to further expand cropland into natural areas (*35*, *36*). In the United States, marginal cropland, much of which is unprofitable (*37*), could be restored to grassland or forests with net societal benefits (*38*). Similarly, NCS may compete with bioenergy production. However, this conflict can be reduced or avoided depending on the form of bioenergy production or NCS. Some forms of biomass production, such as residues and wastes, or high-yielding methods, such as algae, do not require productive land (*39*). Our grassland restoration pathway could produce a limited amount of additional biomass while maintaining carbon sequestration in soils if low-productivity croplands are converted to perennial energy grasses (*40*). Further, NCS based on improved management of existing land uses do not create land use conflict and can even increase productivity within that land use (e.g., fire management or cover crops). However, aggressive expansion of dedicated bioenergy crops, given the large land requirement of both first- and second-generation bioenergy crops (*41*), would be likely to reduce the mitigation potential available through NCS, notably via reforestation, avoided grassland conversion, and natural forest management.

A second concern is that ecosystems have a limited ability to store additional carbon. For each pathway, we quantified the duration of time for which mitigation is expected to occur at the rates we estimate, before saturation effects decrease this rate (table S1). We note that carbon can continue to accumulate in forests for hundreds of years and in soils for centuries or millennia (table S1 and the Supplementary Materials). Further, four of our NCS opportunities (cropland nutrient management, tidal wetland restoration, manure management, and improved rice management) are based on avoided emissions of CH_4_ and N_2_O, which are benefits that do not saturate. The mitigation potential of avoided conversion of habitat is limited by the total carbon contained in the habitat. Our analysis assumes that rates of conversion persist at current levels in a BAU scenario, which would represent a continuing source of emissions for at least 67 years for each habitat considered here before reaching “saturation” when the total area has been lost. However, the long-term benefit of avoided conversion depends on assumed future BAU conversion rates.

The permanence of the ~2270 Pg C currently stored globally in biomass (*42*) and soils to 1 m (*26*) is a significant concern, because unmitigated climate change is likely to cause feedbacks that may increase disturbances such as fire or pest outbreaks (*43*) or limit net ecosystem productivity or forest regeneration (*24*). While NCS would marginally increase this large carbon pool, putting some additional carbon at risk, rapid and widespread implementation of NCS would reduce the overall risk of impermanence to the terrestrial biosphere that unmitigated climate change is likely to cause.

Another challenge is that avoiding conversion in one area can cause conversion to shift to other areas, often referred to as “leakage.” Large-scale sectoral and landscape approaches to land use planning and policies will be needed to realize the NCS opportunities identified here. These approaches can and should be designed to buffer risks of leakage associated with individual projects (*44*).

Reducing carbon-intensive energy consumption is necessary but insufficient to meet the ambitious goals of the Paris Agreement. Comprehensive mitigation efforts that include fossil fuel emission reductions coupled with NCS hold promise for keeping warming below 2°C. Beyond providing meaningful climate mitigation, NCS investment can increase other important ecosystem services. The conservation, restoration, and improved management of lands in the United States represent a necessary and urgent component of efforts to stabilize the climate.

## MATERIALS AND METHODS

Below, we provide a brief overview of methods for each of the 21 NCS that we quantified. Full methodological details are provided in the Supplementary Materials.

Reforestation: Additional carbon sequestration in above- and belowground biomass and soils gained by converting nonforest (<25% tree cover) to forest [>25% tree cover (*45*)] in areas of the conterminous United States where forests are the native cover type. We excluded areas with intensive human development, including all major roads (*46*), impervious surfaces (*47*), and urban areas (*48*). To eliminate double counting with the peatland restoration pathway, we removed Histosol soils (*49*). To safeguard food production, we removed most cropland and pasture. We discounted the carbon sequestration mitigation benefit in conifer-dominated forests to account for albedo effects.

Natural forest management: Additional carbon sequestration in above- and belowground biomass gained through improved management in forests on private lands under nonintensive timber management. The maximum mitigation potential was quantified on the basis of a “harvest hiatus” scenario starting in 2025, in which natural forests are shifted to longer harvest rotations. This could be accomplished with less than 10% reduction in timber supply with new timber supply from thinning treatments for fuel risk reduction until new timber from reforestation is available in 2030.

Fire management: Use of prescribed fire to reduce the risk of high-intensity wildfire. We considered fire-prone forests in the western United States. We assume that treatment eliminates the risk of subsequent wildfire for 20 years, but only on the land that was directly treated. We assume that 5% of lands are treated each year, and we calculated the benefits that accrue over 20 years, finding that the initial increase in emissions associated with prescribed fire treatment is more than offset over time by the avoided impacts of wildfires. We report the average annual benefit across these 20 years. The impact of wildfires includes both direct emissions from combustion and suppression of net ecosystem productivity following wildfires.

Avoided forest conversion: Emissions of CO_2_ avoided by avoiding anthropogenic forest conversion. Most forest clearing is followed by forest regeneration rather than conversion to another land use. To estimate the rate of persistent conversion (i.e., to another land use), we first calculated forest clearing in the conterminous United States from 2000 to 2010 and then used the proportion of forest clearing that historically was converted to another land use to estimate conversion rates in 2000 to 2010. We used estimates of avoided carbon emissions from above- and belowground biomass that are specific to each region and forest type. We did not count forest loss due to fire to avoid double counting with the improved fire management opportunity. We did not count forest loss due to pests because it is unclear whether this loss can be avoided. We reduced the benefit of avoided conversion in conifer-dominated forests to account for their albedo effects.

Urban reforestation: Additional carbon sequestration in above- and belowground biomass gained by increasing urban tree cover. We considered the potential to increase urban tree cover in 3535 cities in the conterminous United States. We considered the potential for additional street trees, and for those cities not in deserts, we also considered the potential for park and yard tree plantings. The potential percent increase in tree cover was estimated on the basis of high-resolution analysis of 27 cities, which excluded sports fields, golf courses, and lawns (*50*).

Improved plantations: Additional carbon sequestration gained in above- and belowground tree biomass by extending rotation lengths for a limited time in even-aged, intensively managed wood production forests. Rotation lengths were extended from current economic optimal rotation length to a biological optimal rotation length in which harvest occurs when stands reach their maximum annual growth.

Cover crops: Additional soil carbon sequestration gained by growing a cover crop in the fallow season between main crops. We quantified the benefit of using cover crops on all of the five major crops in the United States (corn, soy, wheat, rice, and cotton) that are not already growing cover crops (*27*), using the mean sequestration rate quantified in a recent meta-analysis (*51*).

Avoided conversion of grassland: Emissions of CO_2_ avoided by avoiding conversion of grassland and shrubland to cropland. We quantified avoided emissions from soil and roots (for shrubs, we also considered aboveground biomass) based on the spatial pattern of conversion from 2008 to 2012. We used spatial information on location of recent conversion and variation in soil carbon and root biomass to estimate mean annual emission rate from historic conversion. We estimated a 28% loss of soil carbon down to 1 m (*26*). We modeled spatial variation in root biomass based on mean annual temperature and mean annual precipitation using data from (*52*).

Biochar: Increased soil carbon sequestration by amending agricultural soils with biochar, which converts nonrecalcitrant carbon (crop residue biomass) to recalcitrant carbon (charcoal) through pyrolysis. We limited the source of biochar production to crop residue that can be sustainably harvested. We assumed that 79.6% of biochar carbon persists on a time scale of >100 years (*53*, *54*) and that there are no effects of biochar on emissions of N_2_O or CH_4_ (*55*, *56*).

Alley cropping: Additional carbon sequestration gained by planting wide rows of trees with a companion crop grown in the alleyways between the rows. We estimated a maximum potential of alley cropping on 10% of U.S. cropland (15.4 Mha) (*57*).

Cropland nutrient management: Avoided N_2_O emissions due to more efficient use of nitrogen fertilizers and avoided upstream emissions from fertilizer manufacture. We considered four improved management practices: (i) reduced whole-field application rate, (ii) switching from anhydrous ammonia to urea, (iii) improved timing of fertilizer application, and (iv) variable application rate within field. We projected a 4.6% BAU growth in fertilizer use in the United States by 2025. On the basis of these four practices, we found a maximum potential of 22% reduction in nitrogen use, which leads to a 33% reduction in field emissions and a 29% reduction including upstream emissions.

Improved manure management: Avoided CH_4_ emissions from dairy and hog manure. We estimated the potential for emission reductions from improved manure management on dairy farms with over 300 cows and hog farms with over 825 hogs. Our calculations are based on improved management practices described by Pape *et al*. (*8*).

Windbreaks: Additional sequestration in above- and belowground biomass and soils from planting windbreaks adjacent to croplands that would benefit from reduced wind erosion. We estimated that windbreaks could be planted on 0.88 Mha, based on an estimated 17.6 Mha that would benefit from windbreaks, and that windbreaks would be planted on ~5% of that cropland (*8*).

Grazing optimization: Additional soil carbon sequestration due to grazing optimization on rangeland and planted pastures, derived directly from a recent study by Henderson *et al*. (*58*). Grazing optimization prescribes a decrease in stocking rates in areas that are overgrazed and an increase in stocking rates in areas that are undergrazed, but with the net result of increased forage offtake and livestock production.

Grassland restoration: Additional carbon sequestration in soils and root biomass gained by restoring 2.1 Mha of cropland to grassland, equivalent to returning to the 2007 peak in CRP enrollment. Grassland restoration does not include restoration of shrubland.

Legumes in pastures: Additional soil carbon sequestration due to sowing legumes in planted pastures, derived directly from a recent global study by Henderson *et al*. (*58*). Restricted to planted pastures and to where sowing legumes would result in net sequestration after taking into account potential increases in N_2_O emissions from the planted legumes.

Improved rice management: Avoided emissions of CH_4_ and N_2_O through improved practices in flooded rice cultivation. Practices including mid-season drainage, alternate wetting and drying, and residue removal can reduce these emissions. We used a U.S. Environmental Protection Agency (EPA) analysis that projects the potential for improvement across U.S. rice fields, in comparison with current agricultural practices (*59*).

Tidal wetland restoration: In the United States, 27% of tidal wetlands (salt marshes and mangroves) have limited tidal connection with the sea, causing their salinity to decline to the point where CH_4_ emissions increase (*30*). We estimated the potential for reconnecting these tidal wetlands to the ocean to increase salinity and reduce CH_4_ emissions.

Peatland restoration: Avoided carbon emissions from rewetting and restoring drained peatlands. To estimate the extent of restorable peatlands, we quantified the difference between historic peatland extent [based on the extent of Histosols in soil maps (*60*)] and current peatland extent. Our estimate of mitigation potential accounted for changes in soil carbon, biomass, and CH_4_ emissions, considering regional differences, the type of land use of the converted peatland, and whether the peatland was originally forested.

Avoided seagrass loss: Avoided CO_2_ emissions from avoiding seagrass loss. An estimated 1.5% of seagrass extent is lost every year (*61*). We assumed that half of the carbon contained in biomass and sediment from disappearing seagrass beds is lost to the atmosphere (*62*).

Seagrass restoration: Increased sequestration from restoring the estimated 29 to 52% of historic seagrass extent that has been lost and could be restored (*61*). We estimated the average carbon sequestration rate in the sediment of seagrass restorations based on data from six seagrass restoration sites in the United States (*63*).

## Supplementary Material

http://advances.sciencemag.org/cgi/content/full/4/11/eaat1869/DC1

## SUPPLEMENTARY MATERIALS

Supplementary material for this article is available at http://advances.sciencemag.org/cgi/content/full/4/11/eaat1869/DC1 Supplementary Materials and Methods Fig. S1. Mapped reforestation opportunity areas in the lower 48 states. Fig. S2. Conceptual framework for improved forest management carbon accounting. Fig. S3. MAC for carbon sequestration through forest management and aging, after Golub *et al*. (*99*). Fig. S4. MAC for natural forest management after Latta *et al*. (*98*) and best-fit functions. Fig. S5. MAC curves for improved plantations. Fig. S6. Fire management analysis area. Fig. S7. Regions used for reporting avoided forest conversion results. Fig. S8. Forest conversion from 1986 to 2000. Fig. S9. Potential carbon emissions from areas at high risk of forest conversion. Fig. S10. Cities included in the urban reforestation analysis. Fig. S11. Calibration of remote sensing data for forest cover estimation in urban areas. Fig. S12. Avoided grassland conversion map. Fig. S13. MAC curve for avoided grassland conversion. Fig. S14. Nitrogen fertilizer use in the United States. Fig. S15. Marginal abatement cost curve for reducing N fertilizer rate. Fig. S16. Marginal abatement cost curve for applying variable rate technology fertilizer application. Fig. S17. Grazing optimization map. Fig. S18. Legumes in pastures map. Fig. S19. Grassland restoration map. Fig. S20. MAC curve for grassland restoration. Fig. S21. Break-even prices for GHG abatement from rice production. Fig. S22. MAC curve for salt marsh restoration. Fig. S23. MAC of avoided GHG emissions from seagrass. Table S1. Mitigation potential of NCS in 2025. Table S2. Co-benefits of NCS. Table S3. Literature MAC estimates for reforestation of agricultural lands. Table S4. Literature estimates of reforestation costs used to estimate MAC of reforesting natural ecosystems. Table S5. Estimated marginal abatement cost of fire management by major forest region. Table S6. Forest disturbance rates by source. Table S7. Mean annual forest hectares cleared per year from 1986 to 2000. Table S8. Mean annual forest hectares cleared per year from 2001 to 2010. Table S9. Mean annual forest hectares converted per year from 1986 to 2000. Table S10. Proportion of areas cleared from 1986 to 2000 that had not regenerated to forest by 2010. Table S11. Mean predisturbance dry biomass (kg m^−2^) in forest areas converted from 1986 to 2000. Table S12. Mean predisturbance dry biomass (kg m^−2^) in forest areas converted from 2001 to 2010. Table S13. Carbon emissions (Mg C year^−1^) from estimated forest conversion from 2001 to 2010. Table S14. Albedo-adjusted carbon emissions equivalent (Mg C_e_ year^−1^) from estimated forest conversion from 2001 to 2010. Table S15. Urban reforestation maximum potential annual net C sequestration in 2025. Table S16. Uncertainty in urban reforestation average annual abatement (Tg CO_2_) by 2025 at a cost of USD 100 per Mg CO_2_. Table S17. Profitability impacts of cover crops for selected crops. Table S18. Marginal abatement costs of cover crops in the five primary crops. Table S19. Maximum feasible N_2_O reduction for multiple nitrogen fertilizer practices. Table S20. Results from the literature of the potential for reducing N fertilizer rate using within-field management. Table S21. Current and projected GHG emissions from nitrogen fertilizer manufacturing in the United States. Table S22. Mitigation potential for grazing optimization and legumes in pasture NCS at different marginal abatement costs. Table S23. Areas and carbon fluxes for Histosols in the conterminous United States. Table S24. Peatland restoration mitigation calculations for climate zones within the United States. Table S25. 95% CIs for Histosol calculations. References (*64*–*398*)
