# Supplementary material for: Natural climate solutions for the United States
Source: Sci Adv. 2018 Nov 14;4(11):eaat1869. doi: 10.1126/sciadv.aat1869 (PMC6235523; doi:10.1126/sciadv.aat1869)
Supplement: http://advances.sciencemag.org/cgi/content/full/4/11/eaat1869/DC1 [file supp_4_11_eaat1869__index.html]

Science Advances | Science Advances

## Supplementary Materials

**This PDF file includes:**

- Supplementary Materials and Methods
- Fig. S1. Mapped reforestation opportunity areas in the lower 48 states.
- Fig. S2. Conceptual framework for improved forest management carbon accounting.
- Fig. S3. MAC for carbon sequestration through forest management and aging, after Golub *et al*. (*99*).
- Fig. S4. MAC for natural forest management after Latta *et al*. (*98*) and best-fit functions.
- Fig. S5. MAC curves for improved plantations.
- Fig. S6. Fire management analysis area.
- Fig. S7. Regions used for reporting avoided forest conversion results.
- Fig. S8. Forest conversion from 1986 to 2000.
- Fig. S9. Potential carbon emissions from areas at high risk of forest conversion.
- Fig. S10. Cities included in the urban reforestation analysis.
- Fig. S11. Calibration of remote sensing data for forest cover estimation in urban areas.
- Fig. S12. Avoided grassland conversion map.
- Fig. S13. MAC curve for avoided grassland conversion.
- Fig. S14. Nitrogen fertilizer use in the United States.
- Fig. S15. Marginal abatement cost curve for reducing N fertilizer rate.
- Fig. S16. Marginal abatement cost curve for applying variable rate technology fertilizer application.
- Fig. S17. Grazing optimization map.
- Fig. S18. Legumes in pastures map.
- Fig. S19. Grassland restoration map.
- Fig. S20. MAC curve for grassland restoration.
- Fig. S21. Break-even prices for GHG abatement from rice production.
- Fig. S22. MAC curve for salt marsh restoration.
- Fig. S23. MAC of avoided GHG emissions from seagrass.
- Table S1. Mitigation potential of NCS in 2025.
- Table S2. Co-benefits of NCS.
- Table S3. Literature MAC estimates for reforestation of agricultural lands.
- Table S4. Literature estimates of reforestation costs used to estimate MAC of reforesting natural ecosystems.
- Table S5. Estimated marginal abatement cost of fire management by major forest region.
- Table S6. Forest disturbance rates by source.
- Table S7. Mean annual forest hectares cleared per year from 1986 to 2000.
- Table S8. Mean annual forest hectares cleared per year from 2001 to 2010.
- Table S9. Mean annual forest hectares converted per year from 1986 to 2000.
- Table S10. Proportion of areas cleared from 1986 to 2000 that had not regenerated to forest by 2010.
- Table S11. Mean predisturbance dry biomass (kg m−2) in forest areas converted from 1986 to 2000.
- Table S12. Mean predisturbance dry biomass (kg m−2) in forest areas converted from 2001 to 2010.
- Table S13. Carbon emissions (Mg C year−1) from estimated forest conversion from 2001 to 2010.
- Table S14. Albedo-adjusted carbon emissions equivalent (Mg Ce year−1) from estimated forest conversion from 2001 to 2010.
- Table S15. Urban reforestation maximum potential annual net C sequestration in 2025.
- Table S16. Uncertainty in urban reforestation average annual abatement (Tg CO2) by 2025 at a cost of USD 100 per Mg CO2.
- Table S17. Profitability impacts of cover crops for selected crops.
- Table S18. Marginal abatement costs of cover crops in the five primary crops.
- Table S19. Maximum feasible N2O reduction for multiple nitrogen fertilizer practices.
- Table S20. Results from the literature of the potential for reducing N fertilizer rate using within-field management.
- Table S21. Current and projected GHG emissions from nitrogen fertilizer manufacturing in the United States.
- Table S22. Mitigation potential for grazing optimization and legumes in pasture NCS at different marginal abatement costs.
- Table S23. Areas and carbon fluxes for Histosols in the conterminous United States.
- Table S24. Peatland restoration mitigation calculations for climate zones within the United States.
- Table S25. 95% CIs for Histosol calculations.
- References (*64*–*398*)

Download PDF

**Files in this Data Supplement:**

- Adobe PDF - aat1869\_SM.pdf
