# Supplementary material for: Natural climate solutions for the United States
Source: Sci Adv. 2018 Nov 14;4(11):eaat1869. doi: 10.1126/sciadv.aat1869 (PMC6235523; doi:10.1126/sciadv.aat1869)
Supplement: http://advances.sciencemag.org/cgi/content/full/4/11/eaat1869/DC1 [file aat1869_SM.pdf]

Science Advances
AAAS

advances.sciencemag.org/cgi/content/full/4/11/eaat1869/DC1

Supplementary Materials for

Natural climate solutions for the United States

Joseph E. Fargione*, Steven Bassett, Timothy Boucher, Scott D. Bridgham, Richard T. Conant, Susan C. Cook-Patton, Peter W. Ellis, Alessandra Falcucci, James W. Fourqurean, Trisha Gopalakrishna, Huan Gu, Benjamin Henderson, Matthew D. Hurteau, Kevin D. Kroeger, Timm Kroeger, Tyler J. Lark, Sara M. Leavitt, Guy Lomax, Robert I. McDonald, J. Patrick Megonigal, Daniela A. Miteva, Curtis J. Richardson, Jonathan Sanderman, David Shoch, Seth A. Spawn, Joseph W. Veldman, Christopher A. Williams, Peter B. Woodbury, Chris Zganjar, Marci Baranski, Patricia Elias, Richard A. Houghton, Emily Landis, Emily McGlynn, William H. Schlesinger, Juha V. Siikamaki, Ariana E. Sutton-Grier, Bronson W. Griscom

*Corresponding author. Email: jfargione@tnc.org

Published 14 November 2018, *Sci. Adv.* **4**, eaat1869 (2018)
DOI: 10.1126/sciadv.aat1869

This PDF file includes:

Supplementary Materials and Methods
Fig. S1. Mapped reforestation opportunity areas in the lower 48 states.
Fig. S2. Conceptual framework for improved forest management carbon accounting.
Fig. S3. MAC for carbon sequestration through forest management and aging, after Golub *et al.* (*99*).
Fig. S4. MAC for natural forest management after Latta *et al.* (*98*) and best-fit functions.
Fig. S5. MAC curves for improved plantations.
Fig. S6. Fire management analysis area.
Fig. S7. Regions used for reporting avoided forest conversion results.
Fig. S8. Forest conversion from 1986 to 2000.
Fig. S9. Potential carbon emissions from areas at high risk of forest conversion.
Fig. S10. Cities included in the urban reforestation analysis.
Fig. S11. Calibration of remote sensing data for forest cover estimation in urban areas.
Fig. S12. Avoided grassland conversion map.
Fig. S13. MAC curve for avoided grassland conversion.
Fig. S14. Nitrogen fertilizer use in the United States.
Fig. S15. Marginal abatement cost curve for reducing N fertilizer rate.
Fig. S16. Marginal abatement cost curve for applying variable rate technology fertilizer application.
Fig. S17. Grazing optimization map.
Fig. S18. Legumes in pastures map.
Fig. S19. Grassland restoration map.
Fig. S20. MAC curve for grassland restoration.
Fig. S21. Break-even prices for GHG abatement from rice production.

Fig. S22. MAC curve for salt marsh restoration.
Fig. S23. MAC of avoided GHG emissions from seagrass.
Table S1. Mitigation potential of NCS in 2025.
Table S2. Co-benefits of NCS.
Table S3. Literature MAC estimates for reforestation of agricultural lands.
Table S4. Literature estimates of reforestation costs used to estimate MAC of reforesting natural ecosystems.
Table S5. Estimated marginal abatement cost of fire management by major forest region.
Table S6. Forest disturbance rates by source.
Table S7. Mean annual forest hectares cleared per year from 1986 to 2000.
Table S8. Mean annual forest hectares cleared per year from 2001 to 2010.
Table S9. Mean annual forest hectares converted per year from 1986 to 2000.
Table S10. Proportion of areas cleared from 1986 to 2000 that had not regenerated to forest by 2010.
Table S11. Mean predisturbance dry biomass (kg m^{-2}) in forest areas converted from 1986 to 2000.
Table S12. Mean predisturbance dry biomass (kg m^{-2}) in forest areas converted from 2001 to 2010.
Table S13. Carbon emissions (Mg C $year^{-1}$) from estimated forest conversion from 2001 to 2010.
Table S14. Albedo-adjusted carbon emissions equivalent (Mg C_e $year^{-1}$) from estimated forest conversion from 2001 to 2010.
Table S15. Urban reforestation maximum potential annual net C sequestration in 2025.
Table S16. Uncertainty in urban reforestation average annual abatement (Tg CO_2) by 2025 at a cost of USD 100 per Mg CO_2.
Table S17. Profitability impacts of cover crops for selected crops.
Table S18. Marginal abatement costs of cover crops in the five primary crops.
Table S19. Maximum feasible N_2O reduction for multiple nitrogen fertilizer practices.
Table S20. Results from the literature of the potential for reducing N fertilizer rate using within-field management.
Table S21. Current and projected GHG emissions from nitrogen fertilizer manufacturing in the United States.
Table S22. Mitigation potential for grazing optimization and legumes in pasture NCS at different marginal abatement costs.
Table S23. Areas and carbon fluxes for Histosols in the conterminous United States.
Table S24. Peatland restoration mitigation calculations for climate zones within the United States.
Table S25. 95% CIs for Histosol calculations.
References (*64–398*)

Supplementary Materials and Methods

Cross-Cutting Methods

Below, we describe some cross-cutting methods and constraints that apply across all 21 NCS pathways before providing detailed methods for each of the NCS pathways.

Maximum potential

We consider additional mitigation potential for natural climate solutions (NCS) in the year 2025. By "additional," we mean mitigation due to human actions taken beyond business-as-usual (BAU) activities in the land use sector as they are expected to occur in 2025. We chose the year 2025 as a reference year for three reasons: 1) its policy relevance to the United States Nationally Determined Contribution under the Paris Agreement, 2) it is distant enough to envision a scaling up of activities by that year, but also 3) soon enough to contribute meaningfully to the urgent need for mitigation of climate change.

We constrain our maximum estimate in order to be consistent with meeting human needs for food and fiber. For food, we constrain the loss of cropland and pasture. For cropland, we assume that 5.1 Mha of cropland can be restored. This is the equivalent of returning to the amount of lands retired from cropland in 2007, when the CRP saw its maximum enrollment. The CRP pays farmers to retire marginal land from production. Enrollment in the program decreased starting in 2008 due to a combination of a policy change (a reduction in the cap on the number of CRP acres) and a spike in commodity prices that made it temporarily profitable to farm marginal lands. Future food demand can be met without expanding cropland via investments in yield increases, closing yield gaps, diet shifts, aquaculture, and biofuels policy, suggesting that some marginal cropland can be retired while meeting food demand (*35*, *36*). For pasture, we estimate that 1.3 Mha could be reforested without impacting livestock production, as described in the reforestation pathway methods. For timber production, we assume that production could be decreased by 10%, equivalent to the coefficient of variation in timber production in the U.S. from 1986 to 2011, as further described in the improved forest management methods. We also constrain our maximum estimate to exclude practices that would harm biodiversity, such as establishing forests where they are not the native cover type (*64*).

Saturation

We recognize that, for most pathways, the mitigation potential that we estimate for 2025 will not persist indefinitely; rather, NCS based on increased sequestration will eventually saturate, given the finite potential for natural ecosystems to store additional carbon. However, such saturation takes decades, if not longer. For each NCS opportunity, we estimate the expected duration of the potential for sequestration at the maximum rate (table S1). Forests can continue to sequester carbon for >100 years (*65*, *66*). Urban trees are more stressed, but can continue to sequester carbon for >40 years (*67*). Restored grasslands can continue to sequester carbon for >55 years (*68*). Seagrasses can continue to sequester carbon for millennia (*69*, *70*). Peatlands can continue to sequester carbon for millennia (*71*). Note that NCS based on avoided emissions of CH_4 and N_2O do not saturate (e.g. cropland nutrient management, improved manure management, improved rice production, and avoided CH_4 emissions from restored tidal marsh); rather, they

continue to provide additional sequestration for as long as the BAU case indicates there is potential for avoided emissions. In the case of seagrass loss, the BAU is for 1.5% loss every year, such that seagrasses would disappear entirely after 67 years.

Uncertainty

To estimate uncertainty of the maximum potential for each NCS opportunity, we considered uncertainty in both the extent (i.e. hectares) and flux (i.e. sequestration or reduced emissions per hectare). Unless otherwise noted, we used the IPCC approach to combining uncertainty (*72*). Where possible, we present 95% confidence intervals based on multiple published estimates. Where a sample of estimates was not available, we report uncertainty as a range, typically based on the minimum and maximum estimates available from the literature. Where enough data exist to calculate the 95% CI, this is indicated by black text (gray text otherwise) in table S1.

To calculate overall uncertainty, we used a Monte Carlo simulation, with 100,000 iterations, to combine uncertainty across all 21 NCS opportunities (*72*), fitting the data with a log-normal distribution when appropriate (e.g., values could not go below zero or a log-distribution was a more appropriate fit and uncertainty was large) and a normal distribution otherwise.

Committed Emissions

For avoided conversion, we include "committed emissions." When conversion of natural ecosystems occurs, carbon is not all emitted in the same year that the conversion occurs. For example, emissions from decaying wood, or from tilled soils, can continue for many years following conversion. Calculating committed emissions simplifies carbon accounting by including all of the avoided emissions in the year that the conversion was avoided. We use an accounting horizon of 20 years and consider any emissions that would have occurred in that timeframe in our estimate of committed emissions.

Marginal Abatement Costs

For each NCS opportunity, we constructed the marginal abatement cost (MAC) curve from the available information in the literature. A marginal abatement cost curve represents the monetary cost of achieving one additional ton of sequestered greenhouse gases (GHG) or avoided GHG emissions and indicates the total quantity of net GHG reductions that can be achieved at different price points. We estimated the total abatement available at USD 10, 50, and 100 Mg CO_2e^{-1}. Prices on the dominant U.S. regulatory carbon market (California) since 2012 continually have exceeded USD 10 (*73*), while prices are around USD 10 CO_2e^{-1} on voluntary carbon markets for offsets that have strong co-benefits (*74*). The social cost of carbon, that is, the economic value of the society-wide damages caused in a given year by one additional ton of carbon dioxide emissions or its equivalent, is projected to be around USD 50 in 2025, depending on the discount rate used (*10*). A price of at least USD 100 is needed to keep the 100-year average temperature from warming more than 2.5 °C (*11*). All cost estimates presented in this study are expressed in 2015 dollars using the U.S. Bureau of Labor Statistics' Consumer Price Index (*75*).

In some instances, MAC curves from the literature exceeded the maximum potential identified in our analysis. For example, a literature MAC might go to 200 Tg CO_2e yr^{-1}, but we may identify 100 Tg CO_2e yr^{-1} as the limit in our biophysical analysis. In these cases, we use the literature MAC curve up to the 100 Tg CO_2e yr^{-1} that we identified as the maximum mitigation potential.

Characterizing Co-Benefits
NCS have the potential to produce strong co-benefits (table S2). We reviewed the literature for evidence that each pathway produces co-benefits from one or more of four generalized types of ecosystem services (biodiversity, water, soil, air). We define biodiversity benefits as any increases in alpha, beta, and/or gamma diversity as is described in the Convention on Biological Diversity (*76*). Water ecosystem benefits include hydrologic regulation, water purification, and storm protection as defined in the Millennium Ecosystem Assessment (*77*). Soil benefits include improved water holding capacity and fertility as described in the Millennium Ecosystem Assessment (*77*). We define air benefits as improvements in air quality as described in the Millennium Ecosystem Assessment (*77*).

Additional mitigation required to meet Paris Agreement NDCs
The United States' NDC for the Paris Agreement is a 26-28% reduction in emissions compared to a 2005 baseline. The EPA quantifies net emissions in 2005 as 6,582.3 Tg CO_2e, which indicates that net emissions of 4,739.3-4,870.9 Tg CO_2e in 2025 are required to meet the NDC. The EPA (*78*) quantified net emissions in 2015 as 5,827.7 Tg CO_2e. To estimate BAU emissions in 2025, we used EIA projections which predict an increase in energy sector emissions of 91 Tg CO_2e yr^{-1} between 2015 and 2025. We used the EIA scenario without the clean power plan to reflect the current administration's proposal to repeal the clean power plan. This suggests a 2025 BAU net emissions of 5,918.7 Tg CO_2e, requiring an additional 1,047.8-1,179.4 Tg CO_2e of mitigation to meet the NDC.

<u>Reforestation</u>
We define reforestation as conversion from non-forest (<25% tree cover) to forest [>25% tree cover (*45*)], in areas where the historical natural vegetation is forest. Note that, in order to avoid conflicts with biodiversity protection, our analysis excludes planting trees in areas where they do not naturally grow, which is sometimes called afforestation (*64*, *79*, *80*).

Mapping Reforestation Opportunity
We created a novel 30 m resolution raster map of reforestation opportunity in the conterminous United States (CONUS) (fig. S1). To do so, we first identified all areas that historically had forest cover >25% using LANDFIRE Biophysical Setting (BPS) data (*81*) (N = 152 forested BPS groups). This created a map of all CONUS areas with potential vegetation of >25% forest cover. We also included an additional 40 marginal BPS groups where forest cover depends on spatial scale and/or environmental conditions. These latter types include ecosystems with highly variable historical tree cover including many savannas, woodlands, and forest-grassland mosaics (note that we treated these latter cover types differently; see below).

From this map, we removed existing forests using the National American Forest Dynamics (NAFD) 2010 map (*82*). We next removed areas with intensive human development, including all major roads (*46*), impervious surfaces [National Land Cover Data (*47*)], and urban areas (*48*). Removing urban areas also eliminated double counting with the urban forestry pathway. To eliminate double counting with the peatland restoration NCS, we removed pixels with >5% Histosol soils (*60*). Where soils data were missing (7% of land area), we used the BPS vegetation map to remove riparian vegetation (*81*). The resulting map included a total of 154.4 Mha of land potentially available for reforestation.

Deductions
We then applied a suite of deductions to either eliminate or down-weight areas where reforestation is less feasible. These deductions were primarily non-spatial; we did not specify the exact pixel where they should occur, but rather applied proportional deductions across areas that met relevant criteria.

For the 40 marginal BPS groups (i.e., those with potentially less than 25% forest cover), we applied a 50% deduction, assuming only half of these lands would meet our 25% forest cover threshold. Moreover, reforestation efforts in the remaining marginal areas should proceed with caution and seek to maintain natural disturbance regimes (e.g. fire), as well as a natural balance of trees and herbaceous species.

To protect food production we removed almost all croplands in the 2015 National Agricultural Statistics Service Cropland Data Layer that overlapped with our reforestation map (*83*). We retained <1% of the reforestable cropland, based on a U.S. Department of Agriculture (*8*) report that developed a carbon sequestration scenario where 0.36 Mha of riparian forest buffers (out of a total of 42.8 Mha of cropland in the U.S.) are reforested through the CRP (*8*).

Next, we deducted background reforestation rates, because we are interested in additional reforestation opportunities beyond what is already occurring. We calculated background reforestation rates from the annual average forest gain between 1986 and 2010 (NAFD) (*82*) in each U.S. Forest Service (USFS) forest type, and assumed that historical reforestation rates will continue through 2025. Note that this deduction effectively removes areas that regrow without intervention after natural forests are clear-cut a or where plantation forests are harvested and re-planted, which prevents double counting with our natural forest management pathway and improved plantations pathway, respectively.

After these deductions, 79.0 Mha remained available for reforestation. This represents the maximum potential reforestation extent for CONUS, which includes the reforestation of 17.5 Mha of pasture, and therefore would likely require dietary shifts that reduce meat consumption.

Finally, we estimated how much reforestation is possible without impacting livestock production by removing 92.4% of all pasture lands in the 2011 National Land Cover Data (NLCD) (*84*) that overlapped with the remaining reforestable area. We estimated that 7.6% of pasture land could be reforested without compromising livestock production. This is based on trends in beef cattle production efficiency between 1996 and 2014 (*85*), which show that production has held steady even while the number of animals has decreased. During this time, the number of beef cattle in the U.S. has been decreasing linearly at approximately 270,000 head per year, on average. Extrapolating this trend to 2025 we find that the head of cattle will decrease by 7.6% between 2017 and 2025. We assume that the need for pastureland will decrease proportionally. We apply this percentage to only those pasture acres that we deem reforestable rather than to all pastureland, making the deduction more conservative. After all of these deductions, the remaining area available for reforestation is 62.9 Mha.

Estimating Carbon Sequestration

We used USFS yield tables to estimate carbon sequestration rates (*86*). These tables provide estimates of live tree carbon, above and belowground, at different time steps [Appendix A/B in (*86*)]. The estimates are specific to both forest type and USFS region. For each forest type and region combination, we calculated mean annual sequestration per hectare over the first 20 years. This provides an average growth rate over a time span that is relevant to climate mitigation and is shorter than harvest age for forests that might be reforested as a timber plantation. When there was no 20-year data point, we calculated sequestration rates with the 15 and 25-year data and used the average of those two values. Sequestration rates ranged from 0.31 Mg C ha^{-1} yr^{-1} (Ponderosa Pine, *Pinus ponderosa*) to 3.15 Mg C ha^{-1} yr^{-1} (Douglas-fir, *Pseudotsuga menziesii*). To these values we added a soil carbon accumulation rate of 0.09 Mg C ha^{-1} yr^{-1} for all forest types in all regions, based on published reforestation soil carbon accumulation rates (*87*).

To assign these sequestration rates to the LANDFIRE BPS types, we matched the USFS cover types to the BPS types, using the National Forest Type Dataset (*88*). We constructed a crosswalk based on 1) spatial overlap, 2) cover type name similarity, and 3) environmental similarity (e.g., riparian species). In cases where the USFS yield tables did not include a carbon estimate for a specific forest type and regional combination, we used forest type data from the most climatically and geographically proximate region.

When calculating the mitigation benefit of reforestation, we applied one final deduction. We halved the carbon sequestration rates of conifer-dominated forest types to account for the albedo-driven warming that is associated with increased conifer cover (*89*). We then area-weighted the sequestration rate for each BPS cover type to calculate an average reforestation sequestration intensity of 1.33 Mg C ha^{-1} yr^{-1}. Multiplying this value by the feasible reforestation extent of 62.9 Mha gave us a total mitigation potential of 306.6 Tg CO_2e yr^{-1}. If we allow reforestation of all reforestable pasture lands, the total mitigation potential increases to 381.3 Tg CO_2e yr^{-1} on 79.0 Mha.

Uncertainty for reforestation

There are multiple estimates of reforestation opportunity in the U.S. (*90–94*). These range from 7.9 Mha to 134.3 Mha, and are highly variable due to the diversity of the methods used to quantify extent. Across all studies, the average extent was 68 Mha with a 95% CI of ±46%, which aligns well with our own estimate. Applying this uncertainty gives an estimated feasible reforestation extent of 62.9 Mha (95% CI: 34.0 to 91.8)

To quantify the uncertainty of our reforestation sequestration rate, we acquired the original data in Smith *et al.* (*86*) for stands between 1 and 25 years old. We used these data to estimate percent uncertainty for flux by fitting the data with a log-normal distribution and running a Monte Carlo simulation (100,000 iterations) that took into account the relative area of each Forest Service cover type. We used resulting percent uncertainty to calculate a 95% CI of 0.17 to 5.01 Mg C ha^{-1} yr^{-1}.

Marginal abatement costs for reforestation

To estimate potential annual sequestration at USD 100 Mg CO_2e^{-1}, USD 50 Mg CO_2e^{-1} and USD 10 Mg CO_2e^{-1}, we partitioned the areas of reforestation opportunity by their current land uses, to

reflect different marginal costs on agricultural (cropland and pasture) and other land. For reforestation of agricultural lands, we construct a MAC curve from MAC points reported in four of the five studies included in the most recent comprehensive literature review of the cost of reforestation on U.S. agricultural lands (*7*). These studies report mitigation and associated MAC estimates for time horizons of 20 years or less, or allow us to calculate those estimates from their reported results. We exclude the fifth study (*95*) that uses the same model (FASOM-GHG) used in two more recent studies included in our MAC analysis (*96*, *97*) but that is over a decade old and reports much lower reforestation costs. The four included studies use partial or general equilibrium models and report higher MAC for reforestation than studies using econometric models (*7*).

Latta *et al.* (*98*) estimate annualized MAC of reforestation on U.S. private lands using a 20-year timeframe. Their analysis is based on the FASOM-GHG sectoral optimization model of the U.S. forest and agricultural sectors, modified to allow incorporation of two offset program types—voluntary and mandatory. We estimate a best-fit (power) function for Latta *et al.*'s (*98*) data points and use that function to estimate the abatement levels achievable at our three carbon prices (USD 10, 50, and 100 Mg CO_2e^{-1}, respectively).

Alig *et al.* (*96*) use the FASOM-GHG model to estimate cumulative sequestration from reforestation of pasture and crop lands over a 45-year time horizon at prices of USD 0, 25, and 50 Mg CO_2e^{-1}, respectively. We use their base rate development scenario and their estimated total cumulative net sequestration during the first ten years of their analysis horizon in their base case (no carbon pricing) and carbon pricing scenarios (USD 25 Mg CO_2e^{-1} and USD 50 Mg CO_2e^{-1}) to calculate average annual sequestration gains for these price points during that 10-year period.

Haim *et al.* (*97*) also use FASOM-GHG to estimate reforestation and GHG stocks under a hypothetical national carbon market and two carbon prices (USD 30 Mg CO_2e^{-1} and USD 50 Mg CO_2e^{-1}) over a 45-year time horizon, but also provide results for a 20-year horizon. To estimate abatement at USD 10 Mg CO_2e^{-1} and USD 100 Mg CO_2e^{-1} from Alig *et al.*'s (*96*) and Haim *et al.'s* (*97*) data, we fit exponential MAC curves to the two data points in each study. This is a conservative assumption as other studies suggest a linear or logarithmic functional form (*98*, *99*), which would result in lower MAC for given abatement levels. Golub *et al.* (*99*) estimate MAC for both avoided forest conversion and reforestation combined. We use a best-fit (polynomial) function to their seven data points to estimate abatement at our three carbon price points.

We construct mean abatement estimates at the three carbon prices as the unweighted average of the abatement quantities calculated from the four studies using the best-fit MAC functions described above (table S3). Where MAC curve-based estimates of abatement quantities for given carbon prices exceed the abatement potential associated with our feasible implementation level, we constrain abatement to the latter. Our estimated MAC do not include any transaction costs that may be associated with certification, monitoring, and enforcement needed to implement economic incentives for forest-based carbon mitigation because none of the studies we used to construct our MAC estimates include those costs.

To determine how the other (non-crop and non-pasture) areas are currently used, we cross-referenced our spatial map of reforestation opportunity with the 2011 NLCD (*84*). Note that this spatial analysis does not incorporate the non-spatial deductions described above, but we assumed that the non-spatial deductions applied uniformly across NLCD types and that relative proportions would remain constant. We found that the current land use fell into four categories: production lands (e.g., pasture and crop), natural ecosystems (e.g., forests and shrublands), low human development areas (e.g., parks), and barren lands (e.g., mine lands) or those with perennial snow/ice. Excluding production lands, we found that 82% of our spatial reforestation pixels fell into natural ecosystems, 2% into low human development areas, and 17% into barren and perennial snow lands. We assumed that the latter two categories would be cost-prohibitive to reforest for carbon sequestration, because of the high opportunity cost of converting recreational areas and the high implementation cost of reforesting barren lands. For the remaining 82% (or 50.1 Mha) that fell into natural ecosystems, we assumed zero opportunity cost for conversion and estimated MAC based on only the costs of forest reestablishment.

To estimate the cost of reforesting natural ecosystems, we use the mean of the per hectare reforestation cost reported in several studies (*100–104*) (table S4). We annualize costs over nine years using the average September 2012 to September 2017 U.S. Treasury Bond yield rate of 2.2% (*105*). Using the estimated mean sequestration rate of 1.33 Mg C ha^{-1} yr^{-1}, we estimate the mean MAC of reforesting natural ecosystems as USD 21 Mg CO_2e^{-1}. Thus, we estimate that none of these natural lands would be reforested with a USD 10 Mg CO_2e^{-1} price on carbon and that all of them would be reforested at USD 50 Mg CO_2e^{-1}.

<u>Natural Forest Management and Improved Plantations</u>

Natural forest management includes changes in timber management practices to increase net forest carbon sequestration. The natural forest management pathway covers mixed native species forests under private ownership, which primarily occur in the eastern continental U.S. Public forests are generally already managed with longer rotations, such that the potential for increasing carbon storage on these lands through the improved management practices considered here is negligible. We considered activities that maintain long-term wood harvest levels, but may constrain near and mid-term harvest levels. Our estimate for maximum biophysical potential in natural forests is based on a harvest hiatus of ≥25 years for private forests already under timber management (excluding plantations, which are covered in the improved plantations pathway). While this calculation determines our estimate of the maximum potential, we note that other activities (e.g. reduced-impact logging, thinning treatments) can deliver more constrained mitigation levels for this pathway without delaying wood harvest.

The improved plantations pathway covers intensively managed planted monoculture forests, which primarily occur in southeastern and northwestern U.S. Our estimate of maximum mitigation potential for the improved plantations pathway is based on extending rotations by 5 to 20 years, depending on region. We quantify this potential on private, intensively-managed forest in the two major production centers of the U.S. (the South and the Pacific Northwest).

We considered forest land in the U.S. (including Alaska and Hawaii) in timber production as of 2012 based on the latest U.S. Forest Service Resources Planning Act (RPA) Assessment (*106*).

We considered carbon pools in live tree biomass (aboveground and belowground), coarse woody debris, and harvested wood products. The term "improved forest management" (IFM) includes both natural forest management and improved plantations. Mitigation potential is quantified as the difference in annual forest carbon stock changes between an IFM and a BAU scenario, where

$$\textit{Maximum mitigation potential} = \textit{stock}\ \Delta\ \textit{IFM} - \textit{stock}\ \Delta\ \textit{BAU}$$

For each scenario, stock change is quantified as the net of carbon sequestration via forest growth and carbon emissions due to harvest and natural disturbance (e.g. fire, pests, wind, ice). We assume that carbon emissions from natural disturbance are the same under IFM and BAU scenarios. While we note that there are interactions between harvest regimes and frequency and severity of natural disturbance events, the direction of cumulative harvest impacts on natural disturbance is ambiguous and we find no clear basis for altering the assumption of equal natural disturbance in BAU and IFM scenarios. For example, susceptibility to wind-throw and ice damage may increase post-thinning, while thinning activities may improve residual tree vigor and reduce incidence of forest pest infestation (*107–111*). Assuming no change in natural disturbance between BAU and IFM allows us to define changes in stocks based on growth and harvest alone

$$\textit{stock}\ \Delta\ \textit{IFM} = \textit{forest growth IFM} - \textit{harvest IFM}$$
$$\textit{stock}\ \Delta\ \textit{BAU} = \textit{forest growth BAU} - \textit{harvest BAU}$$

We further simplify this equation by assuming no change in average forest growth rates during the first 25 years of deferred harvests. While net sequestration rates can be expected to reach a peak and then decline as forests mature (*112*), evidence supports the assumption of no change from BAU in mean net sequestration rates within the first 25 years of deferred harvests. Over the last 25 years, estimates of forest growth per unit area in the U.S. have remained fairly constant, ranging between 3.9 to 5.1 Mg CO_2 ha^{-1} yr^{-1} net ecosystem productivity. There have been no apparent trends from 1990 to 2013, despite generally maturing age class distributions (*113–118*). Further, stopping logging in these mostly uneven-aged systems would avoid the short-term reductions in growth rates following harvest (*14*, *119–121*), making our overall assumption of no change in growth rates a conservative one. Eventual reductions in growth rates would be expected to occur where harvests are deferred indefinitely. Therefore, based on the literature reviewed above, we assume that this pathway begins to saturate after 25 years.
With these assumptions, and only applying these equations to the years before saturation occurs, forest growth is equivalent under BAU and IFM. Consequently, our accounting for both natural forests and plantations simplifies to become a function of relative harvest emissions

$$\textit{Maximum mitigation potential} = \textit{harvest BAU} - \textit{harvest IFM}$$

Harvest Reduction Scenarios for Improved Plantations
The IFM NCS involve extending rotations on private lands in two intensively managed softwood forest regions in the U.S.: The South and Pacific Northwest (PNW). In 2011, these two sources made up 88% of U.S. plantation acreage (*122*) and 49% of total U.S. timber production, as calculated from 2012 RPA data (*106*). Most of these production landscapes are currently managed on short, economically optimal rotations. Under BAU, when the growth rate of timber

in the field is less than the growth rate of cash in the bank, the trees are harvested and sold so that the capital can be reinvested to maximize returns. Note that, if harvest is delayed past the economically optimal rotation length, while the growth rate may decline as a percentage of standing timber, the quantity of wood and carbon added to these forests annually continues to increase for a period of time. Under IFM, the rotation length is moved from the economic optimum to the biological optimum. In each landscape, we set the target extended rotation length as the biological optimum rotation of the dominant timber species. The biological optimum is defined as the point at which the maximum annual increment (MAI) occurs, representing a rotation length for production forests that would increase long-term average timber output.

The carbon benefit of extending rotations is realized during the transition between management regimes, which is essentially a stop-harvest period resulting in increased standing forest carbon. After the stop-harvest period, production is resumed at former levels (now with higher stocking per unit area at harvest age, but with a smaller area harvested each year) and harvest emissions return to BAU levels.

For improved plantations, we impose a constraint to maintain the intensively managed forest landscape, allowing no more than 10% reduction in harvest output at any point in time from the private plantation forestry landscape. This level of reduction falls within the historic range of fluctuations in timber production in the U.S. Specifically, from 1986 to 2011, the coefficient of variation (average deviation from the mean as a percent) of annual timber production was 10.6% [calculated from 2012 RPA data (*106*)].

To constrain reductions in annual harvest to 10% of total harvest, we simply reduce annual harvest from historic levels by 10%, and postpone harvest of these forests until they reach the biological optimum. Thus, these areas are subject to a stop-harvest period equal to the biological rotation age minus the economic rotation age. Once a stop-harvest period is over, those older forests start being harvested and an additional ten percent of younger forests can enter the stop-harvest period (i.e., from 10% to 20%, then from 20% to 30%, etc.). In this manner, with only a 10% reduction in overall production in any year, all of the forests would be shifted to a longer rotation over the length of the stop-harvest period multiplied by ten. Such a transition would generate increased carbon sequestration in forests for that entire time. At the end of this transition there is no additional net sequestration (and timber production returns to BAU levels or higher).

Softwood forests on private lands in the South would undergo a stop-harvest period of five years to extend economically optimum rotation to a biologically optimum rotation. In the South, 94% of 2009 softwood timber outputs are from southern pines (Loblolly, *Pinus taeda*; Shortleaf, *Pinus echinata*; Slash, *Pinus elliottii*; Longleaf, *Pinus palustris*) (*123*), and 62% of the acreage of these species is in planted stands [calculated from 2012 RPA data (*106*)]. Under the improved plantations pathway, rotations in these forests can be extended from the typical intensive private management regime of 20-year rotation [review of age class distributions derived from 2012 RPA data (*106*, *122*)], to a 25-year biologically optimum rotation (*86*). As a result, the period of emission reductions from the BAU is 50 years (5 years * 10 cohorts).

Softwoods on private lands in the PNW would undergo a stop-harvest period of 15 to 20 years to extend economically optimum rotation to a biologically optimum rotation. In the PNW, softwood timber outputs are dominated by Douglas-fir, and 55% of Douglas-fir acreage is in planted stands [calculated from 2012 RPA data (*106*)]. Under the improved plantations pathway, rotations in these forests (e.g. Douglas-fir) can be extended from the typical intensive private management regime of 40 to 45-year rotations [review of age class distributions derived from 2012 RPA data (*106*, *122*, *124*)] to 60-year biologically optimum rotation (*86*). As a result, emission reductions would continue for 150 to 200 years (15-20 years * 10 cohorts).

Harvest Reduction Scenarios for Natural Forest Management
The natural forest management pathway considers natural forests on private lands under uneven-aged and less intensive management. These less intensively managed forests have a wide range of typical rotation lengths and harvest approaches (e.g. selective harvests). Here we model reductions in harvest-associated emissions from stopping harvest entirely between 2025 and 2050, shifting forests to overall more mature conditions, after which production would resume while retaining increased standing timber biomass compared to BAU.

Given this "stop logging" scenario we use to calculate maximum mitigation potential of the natural forest management pathway, and our requirement that total domestic wood volume production is not reduced below 90% of historical mean production levels (266 million m^3 yr^{-1}), we meet 90% of the production from private natural forests (240 million m^3 yr^{-1}) by other domestic sources between 2025 and 2050.

During 2030 to 2050, new wood production from the reforestation pathway could more than offset lost wood production from natural production forests on private lands (see below for how production is replaced between 2025 and 2030). Although the reforestation pathway is based exclusively on natural regeneration, we find that 52.7% of potentially reforestable lands (see reforestation pathway methods) occur within forest types (*88*) that could support intensive plantation management. If plantations were established in these areas in lieu of naturally regenerating forests, we estimate the new cumulative wood production would be 447 million m^3 in 2030, 1.55 billion m^3 in 2032, and 4.52 billion m^3 by 2048, more than enough wood to fully meet timber demand from stopping harvest on uneven-aged natural forests. While this would require markets to accommodate any shift in the species of timber delivered to market, this potential additional wood production from reforestation with plantations could more than compensate for wood production from private natural forest lands between 2030 and 2050.

To replace production between 2025 and 2030, we estimate that an additional 1,211 million m^3 could be generated by carbon neutral thinning of fire prone western forests to enhance fire resilience (Skog et al. 2006a, Skog et al. 206b). Thinning for fire risk reduction would generate over 240 million m^3 per year between 2025 to 2029. Thus, this material could meet the demand gap, recognizing that this approach requires use of newly available technology, such as cross laminated timber, to enable smaller diameter trees to meet demand for wood products. Removal of this biomass reduces the likelihood of high intensity fires with positive carbon benefits. Alternatively, improved practices that increase forest stocks, such as reduced-impact logging techniques and improved silvicultural methods that release more vigorous stand growth (*125*, *126*), could achieve mitigation without reducing production. These improved silvicultural

practices include thinning from below and crop tree thinning (Davis et al. 2009, Hoover and Stout 2007). Further, increasing vine loads in eastern US forests have been decreasing tree growth rates (Matthews et al. 2016) and cutting vines can increase stand carbon stocks compared to a no-harvest scenario (van der Heijden et al. 2015). Also, we do not account for additional wood production that could be achieved by other means such as genetic improvements to plantation forests and advances to reduce wood waste from improved wood processing technologies and biomass markets (*127*). In summary, there are a variety of potential mechanisms that would achieve the maximum mitigation potential that we quantify, either through substitution from new sources or through improved practices.

Estimating Avoided Emissions – Natural Forest Management and Improved Plantations
We define harvest emissions as all carbon emitted in the first 20 years following harvest from aboveground biomass (AGB), belowground biomass (BGB), coarse woody debris, and harvested wood products pools (i.e. not retained in harvested wood products or coarse woody debris beyond 20 years after harvest) (fig. S2). The principal data source from which estimates were derived was the U.S. Forest Service RPA Assessment (*106*, *122*, *128*).

The BAU scenario assumes constant annual harvest levels. BAU is set as the average of annual roundwood removals (disaggregated by softwood and hardwood) reported for 1986, 1996, and 2006 [calculated from 2012 RPA data (*106*)]. Roundwood is defined as volume harvested for industrial and non-industrial products from growing stock and other sources (non-growing stock, i.e. saplings, stumps, tops, and limbs). Annual removals nationwide were fairly stable from 1986 to 2006, and are currently recovering to pre-2008 economic crash levels (*106*).

To calculate emissions based on roundwood production, we estimate associated emissions that occur in the field (from logging residue and belowground biomass) and at the mill (from mill residue). Ratios of *logging residue volume:roundwood volume* and *mill residue volume:roundwood volume* were calculated from 2012 RPA data (*106*), and are assumed to be constant among scenarios and through the projection period. Belowground biomass was estimated using a root:shoot ratio of 0.2 (*129–131*).

Logging residues (includes both residual portion of trees and trees downed incidentally that are left on the ground following harvest) and belowground biomass remaining after 20 years are calculated by applying an annual coarse woody debris decomposition rate of 0.04 [review of North American values from (*132*)]. Based on this decomposition rate, 56% of logging residue is considered "committed emissions" and are counted in the year the forest is harvested.

The portion of mill residues used as commercial fuel or "not used" is assumed to be emitted immediately at the time of harvest. This proportion is 29% of softwood mill residue in the PNW, 50% of softwood mill residue in the South, and 46% of mill residue in the remainder of U.S. production, calculated from 2012 RPA data and assumed to be constant over projection period. Transformed wood products (= roundwood removals – mill residue used as commercial fuel or "not used") that are retired and oxidized in the first 20 years (i.e. not remaining stored in-use or in landfills after 20 years), applying factors from Smith *et al.* 2006 (*86*), are assumed to be emitted immediately at the time of harvest.

Volume removals (m^3) were converted to Mg CO_2e applying specific gravities of 0.41 g per cm^3 for softwood and 0.45 g per cm^3 for hardwood [U.S. averages applied by (*133*)], a carbon fraction of biomass of 0.5, and a conversion factor of CO_2e to carbon of 44/12.

Results
Average annual harvest emissions in the BAU scenario are estimated to be 389.9 Tg CO_2e. Modeled annual benefits of IFM are 10.1 Tg CO_2e for intensive forestry in the South, 2.1 Tg CO_2e for intensive forestry in the PNW, and 267.2 Tg CO_2e for extensive forestry. Total annual IFM benefit is modeled to be 279.4 Tg CO_2e.

Uncertainty for natural forest management and improved plantations
The derived U.S. BAU estimate of total annual harvest emissions is 106.4 Tg C yr^{-1}. Comparable estimates compiled by Williams *et al.* (*133*) range from 45 to 153 Tg C yr^{-1}, with a 95% CI of ±13%. The uncertainty represents not only differences in emission estimates for the same year, but also variation in emissions from year to year (estimates span the period from 1950 to 2012), and consequently offers a relevant gauge of uncertainty in annual emissions over the 34-year projection period.

Marginal abatement costs for natural forest management
We identified two analyses that estimate MAC for natural forest management in the U.S. for time horizons similar to ours. Using a 20-yr analysis horizon and a 5% discount rate for costs and carbon, Golub *et al.* (*99*) estimate the total average annual U.S. forest carbon supply for seven carbon price points (from USD 2 to USD 183 Mg CO_2e^{-1}) for combined changes in forest management and aging ("intensive margin"). They do not estimate maximum abatement potential for this pathway; at USD 183 Mg CO_2e^{-1} they estimate annual sequestration potential at 1051 Tg CO_2 yr^{-1}. Latta *et al.* (*98*) estimate annual carbon sequestration and MAC from natural forest management of existing private U.S. forests up to prices of USD 49 Mg CO_2e^{-1} under a voluntary carbon program (yielding 60 Tg CO_2e yr^{-1}) and USD 60 Mg CO_2e^{-1} under a mandatory carbon program (yielding 220 Tg CO_2 yr^{-1}), respectively, using a 100-yr analysis horizon and 4% discount rate for land management returns.

We develop best-fit functions for Golub *et al.*'s (2009) MAC point estimates (fig. S3) and Latta *et al.*'s (*98*) voluntary and mandatory MAC point estimates (fig. S4) and use them to calculate the estimated mean MAC as the unweighted average of the three functions at USD 100, 50, and 10 Mg CO_2e^{-1}, respectively.

Marginal abatement costs for improved plantations
Our estimates of the MAC of extending rotation length on forest plantations are based on Sohngen and Brown's (*134*) analysis of the potential costs and quantity of sequestered aboveground carbon from extending rotation ages in softwood forests in 12 states in the southern and western USA using data from over 300 forest types and site classes. (The authors do not estimate changes in soil carbon, citing other studies that find that changes in rotation length do not affect soil carbon). Sohngen and Brown's MAC and sequestration estimates are derived by discounting annual costs and sequestration over 300 years using a 6% discount rate for both costs and carbon. This approach avoids the arbitrary selection of a time horizon required for applying

carbon flow summation or average carbon storage methods. The authors provide aggregate MAC curves for three regions: Southcentral, Southeast and West coast.

To convert Sohngen and Brown's estimates of total discounted sequestration to their annual equivalents (Tg CO_2e yr^{-1}, the metric used for all NCS in our study), we assume that total annual sequestration at given carbon prices is constant over time. This is justified given the large number of plantations, the different ages of trees within and across plantation, and the relatively small number of years (5-20) by which rotations are extended. We calculate average annual sequestration x from Sohngen and Brown's total discounted (present value) sequestration (PV_S) at given carbon prices by rearranging the standard equation used to calculate the present value of a constant annuity paid out once annually over n years, as

$$x = PV_S/(1+(1-(1+r)^{-n})/r)$$

where r and n are the annual discount rate (6%) and time horizon (300 years), respectively, that Sohngen and Brown (*134*) used to calculate total discounted sequestration. Given that Sohngen and Brown (*134*) discount both costs and sequestration at the same annual rate (6%), their MAC estimates (in USD Mg CO_2e^{-1}) are not affected by our conversion of sequestration from discounted *PV* to annual. Figure S5 shows Sohngen and Brown's (*134*) MAC curves with the x-axis rescaled to annual sequestration. Estimated annual sequestration is 0.9 Tg CO_2e yr^{-1} at USD 10 Mg CO_2e^{-1}, 7.8 Tg CO_2e yr^{-1} at USD 50 Mg CO_2e^{-1}, and 12.9 Tg CO_2e yr^{-1} at USD 100 Mg CO_2e^{-1}.
Note that Sohngen and Brown's analysis assumes that landowners receive the carbon payment at the time of harvest rather than annually. This increases the cost of extending rotations compared to annual payments for stored carbon and thus increases estimated MACs. If landowners were to receive annual payments, MACs would be lower than our estimates, all else equal.

Fire Management

We quantified the potential carbon benefits that could result from increasing prescribed fire usage in the western U.S. We did so by comparing two scenarios, BAU scenario and a prescribed fire scenario (Rx) where 5% of the identified land area is burned per year. This represents 0.9 Mha of prescribed fire per year. Current prescribed fire treatments averaged 0.26 Mha yr^{-1} on public lands in the six western USFS regions between 2006 and 2015 (*135*). Increasing the extent of prescribed fire is consistent with calls to reduce the risk of damaging wildfires to communities and their drinking water supplies (*136*).

Analysis Area

We defined the analysis area with LANDFIRE data (*137*), selecting existing vegetation types (EVT) in the western continental U.S. that 1) have a historical mean fire return interval (MFRI) of less than 40 years, 2) are considered forests based on a canopy cover of greater than 25% (*45*), and 3) are types where prescribed fire is appropriately used [see table S1 in (*138*)]. This led to an analysis area of 17 Mha that spanned 18 different EVT cover types. Because of strong geographic differences in vegetation type and fire return intervals, we subdivided the analysis area into seven regions: Black Hills (BH), Cascades (CS), Northern Rockies (NR), Sierra Nevada (SN), Southern Rockies (SR), Southwest (SW), and the Klamath-Siskiyou (KS) (fig. S6). We

delineated regions following Westerling (*139*), and resampled and snapped the canopy cover data to the 30 m LANDFIRE equal area grid.

Carbon Balance

The carbon balance in each scenario is generally determined by the area burned by fire, the amount of carbon initially present, the amount of carbon lost through emissions, and net ecosystem productivity (NEP). For the BAU scenario, the key variables include unburned area, NEP in unburned areas, initial carbon present, extent of wildfire, wildfire emissions, and post-wildfire NEP. For the Rx scenario, the model also includes additional area burned by prescribed fire, prescribed fire emissions, and NEP post-prescribed fire.

Initial Carbon

For each region, we calculated the mean and standard deviation of initial tree carbon using the National Biomass and Carbon Dataset (*140*). We assumed that carbon represented 50% of tree biomass (*141*). For other carbon pools (e.g. herb/shrub, duff/litter, coarse woody debris/fine woody debris) we used the LANDFIRE fuel loading model dataset (*143*). For all forest types, we calculated the mean and standard deviation of each fuel pool, assuming that carbon made up 49% of herb and shrub biomass, 37% of organic duff and litter, and 50% of fine and coarse woody debris (1 to 1000-hour fuel) biomass (*141, 143*).

Fire Emissions

To quantify emissions from wildfire we used percent consumption from Meigs *et al.* (*144*) for three fire severities (low, moderate, high) and two forest types (Ponderosa Pine and Mixed Conifer) to determine a mean and standard deviation for percent consumption of carbon by wildfire. To quantify emissions from prescribed fire we used generic forest type classifications and percent consumption values from Wiedinmyer and Hurteau (*138*) to calculate a mean and standard deviation for percent consumption of carbon by prescribed fire. We calculated prescribed fire emissions for herb and shrub biomass, organic matter biomass, and fine and coarse woody debris biomass, and assumed no loss of carbon from live trees.

Net Ecosystem Productivity

For each region, we calculated mean and standard deviation of NEP for unburned and prescribed-burned areas using values from Collatz *et al.* (*145*). We calculated mean and standard deviation of post-wildfire NEP using values for all three fire severity classes from Meigs *et al.* (*144*) and Dore *et al.* (*146*). Note that these post-wildfire NEP values may be conservative, as recent analyses suggest there have been significant decreases in post-wildfire regeneration after wildfire in the 21st century (*24*).

Extent of Fire

We calculated mean and standard deviation of log-transformed area burned by wildfire per year in each region using data from 2000 to 2014 in the Monitoring Trends in Burn Severity (MTBS) dataset (*147, 148*). For the BAU scenario, we assumed that the distribution of area burned by wildfire per year remained constant over the analysis period. It is important to note that these values inherently include the effect of any on-going treatment effort (e.g. thinning or prescribed burning) and fire suppression activities on area burned. For the prescribed fire scenario, we assumed that on average 5% of each region would be treated each year, leading to complete

treatment after 20 years. To include a measure of inter-annual variability in our prescribed fire area (which can vary due to weather conditions), we assumed that the standard deviation of the area treated per year was 10%. We also assumed that for every hectare treated by prescribed fire, the area burned by wildfire was reduced by one hectare [e.g., a leverage of 1, (*149*)].

Analysis

We used a Monte Carlo simulation to estimate carbon flux per year for both scenarios in each region. For each of 100,000 runs, we used the mean and standard deviation values for each component described above and sampled from normal distributions for each variable, except wildfire extent where we sampled from a lognormal distribution. We calculated the carbon balance for each region for each time-step as

$$C_{balance} = NEP_{WF} + NEP_{Rx} + NEP_{UB} - E_{WF} - E_{Rx}$$

Where *NEP* is net ecosystem productivity for the area burned by wildfire (*WF*), burned by prescribed fire (*Rx*), or unburned (*UB*) and *E* is emissions from wildfire (*WF*) or prescribed fire (*Rx*). We produced model outputs for a simulated 20 years, and tracked cumulative area burned in each time step by summing prior area burned (by prescribed fire and/or wildfire) with the current year's extent. We then multiplied cumulative area by the appropriate post-fire NEP value.

This model produced a mean and 95% CI for the carbon balance per year for both scenarios in each region. We summed the carbon balance across all seven regions and all years, to get an estimate of carbon sequestered under each scenario over a 20-year time horizon.

We found that after 20 years, the prescribed fire scenario sequestered an additional 362 Tg CO_2 compared to the BAU scenario. This leads to a mitigation rate of 18 Tg CO_2e yr^{-1} (95% CI: -5 to 42). It is important to note, however, that the benefits of prescribed fire accrue non-linearly. Initially, the prescribed fire scenario releases more carbon than the BAU scenario because more area is burned. However, because prescribed fire releases fewer emissions and supports higher NEP post-fire than wildfire, eventually more carbon is sequestered in the Rx scenario. While this paper focuses on implementation through 2025, we present an average sequestration rate calculated over a 20-year time horizon to account for the benefit of past prescribed fire treatments that will be realized in future years.

Uncertainty for fire management

We took advantage of the variance produced from the Monte Carlo simulation to calculate overall uncertainty around the mitigation potential of the fire management pathway. We constructed a second Monte Carlo simulation that sampled from normal distributions around the mean carbon balance for each region in each year under the two scenarios. For each of the 100,000 runs, we calculated the difference between the Rx and BAU for each region by year combination and summed these to determine overall mitigation. We used the resulting variance around the 100,000 runs to assess overall uncertainty.

Marginal abatement costs for fire management

Using USFS annual data on cost and treatment extent of prescribed burning by USFS forest region, we calculate the 2012-2015 average cost per hectare of prescribed burning in each USFS

region. We use these USFS region-specific mean costs and the overlap of each of our six regions with the USFS regions to calculate (regional composition-weighted) mean cost per hectare of prescribed burning in each of our six forest regions. We then use these mean costs per hectare and our estimated 20-yr average annual net sequestration per hectare (assuming leverage = 1) in each of the six forest regions, and total hectares in each region treated during 2017-2025 (45% of suitable area in each region) to calculate for each region its estimated mean MAC (USD Mg CO_2e^{-1}; table S5).
Our cost estimates likely are biased upward because prescribed burning reduces wildfire risk (*150*) and associated fire suppression costs and size of damages from wildfires (*151*). Further, managing natural fire ignitions that occur during benign weather conditions can be used to meet the objectives of prescribed fire at a lower cost. We do not account for those cost reductions in our analysis.

Avoided Forest Conversion
We calculate the maximum mitigation potential from avoided forest conversion by estimating the historic quantity of carbon released annually from forest conversion (i.e. deforestation) in CONUS. Most forest areas in the U.S. that are cleared are not converted to another land use. These are temporary clearings, primarily for harvesting timber, which are allowed to regenerate to forest rather than being converted to other land uses. To describe this important distinction, we use the term “cleared” to refer to land that is cleared, but may or may not be converted, and restrict the use of the term “converted” to that subset of cleared land that is converted to another land use instead of regenerating to forest. To quantify emissions from forest conversion, we calculate the biomass emissions from all pixels that experienced anthropogenic forest clearing between 2000 and 2010, and multiply each pixel’s emissions by the proportion of clearings that do not regenerate to forest (i.e. are converted to something other than forest), based on observations of this proportion from 1986 to 2000 within each forest type and USFS region. This approach involves the following sequential calculations, with data sources described further below

$$B_{clear}(\mathrm{x, y}) = B_{preD}(\mathrm{x, y}) * D(\mathrm{x, y})$$

where B_{clear} is the total woody dry biomass per m^2 that was cleared from 2001 to 2010 and mapped for locations (x, y), B_{preD} is the pre-disturbance total woody biomass per m^2 in a given location, and D is a binary variable (0 or 1) that only takes value 1 if a pixel is identified as having been cleared within the time frame of 2001 to 2010.

$$Fc(\mathrm{x, y}) = B_{clear}(\mathrm{x, y}) * Conv(\mathrm{x, y}, for, reg) * Z(\mathrm{x, y}, for, reg) * B_{scale} * CB * EF * A_{pixel}$$

where Fc(x, y) is the committed emissions of carbon from forest conversion, $Conv$ is the proportion of cleared forest that does not regenerate (i.e. is converted from forest), Z is a binary variable (0 or 1) that only takes value 1 for the forest type group and region specified for the given pixel, B_{scale} scales aboveground tree biomass to total woody biomass including belowground biomass, CB is the carbon fraction of dry biomass (assumed to be 0.5), EF is the emissions factor characterizing the proportion (%) of total woody biomass that is released to the atmosphere over the commitment time frame (nominally 20 years), and A_{pixel} is the pixel area (30 m x 30 m = 900 m^2). Lastly, we compute the spatially summed mean annual rate of committed

carbon emissions from forest conversion over the time frame (N_{years} = 10 years) for each forest type group (*for*) and region (*reg*)

$$Fc_{ann}(for, reg) = \Sigma\, Fc(for, reg) / N_{years}$$

Locations cleared each year from 2001 to 2010 ($D(x, y)$) are defined based on data from the NAFD project (*82*). The NAFD project reported CONUS forest and non-forest areas at 30 m resolution for every year from 1986 to 2010 based on Landsat spectral reflectances. The dataset also reports the year of the most recent disturbance, which have been attributed to fire, bark beetle, and other causes (*133*). Most of the *other* category is predominantly harvest and forest clearing activities but also includes some windthrow and severe damage by additional pests and pathogens. By removing natural disturbances such as fire and bark beetle outbreaks, we provide a spatially explicit map of locations that were cleared as a result of human activity from 2000 to 2010. Forested wetlands were also spatially excluded from the analysis, as this would be considered a separate mitigation pathway related to avoided wetland loss. Wetlands were defined as all areas in the gridded Soil Survey Geographic Database (*60*) with a Histosol soil content greater than 5%. Where no soil survey data was available, we used data on the extent of riparian forests identified from the riparian category in the LANDFIRE BPS map (*152*). Urban areas were not excluded, as avoiding forest conversion in urban zones is considered a component of this pathway.

To quantify conversion of forest (*Conv*), we estimated the percentage of cleared area that does note regenerate to forest within 10 years, based on the NAFD dataset. For disturbances that occurred between 1986 and 2000, we identified which of the cleared sites returned to forest within 10 years of disturbance, and which remained non-forest for at least 10 years. The conversion rate for disturbances detected for 2001 to 2010 cannot be confidently assessed because it may take at least 10 years post-disturbance for forest recovery to be detected in Landsat spectral reflectances. Therefore, we assume that the percent of clearing that indicates conversion as measured between 1986 to 2000 is representative of those for 2001 to 2010. The ratio of the total area converted to the total area cleared over the period (1986 to 2000) yields the conversion rate (*Conv*). We computed conversion for each forest type group (*for*) and region (*reg*). We defined forest types using the USFS forest cover definitions (*88*) and regions using the nine CONUS USFS Administrative Regions (*153*) (fig. S7). Large-scale evaluation of the conversion rates obtained with this method shows good agreement with statistics from the US government. Country-wide analysis indicated that about 16% of all non-fire, non-bark beetle forest clearing events did not involve a return to forest within 10 years (table S6). That corresponds to an annual rate of conversion of about 380,417 ha per year, or 0.16% of the 244 Mha of forestland for CONUS as estimated from the NAFD dataset. This is a plausible rate of conversion, broadly consistent with (*154*) who reported a countrywide deforestation rate of about 0.12% per year (or 355,000 ha yr^{-1}), mostly related to housing and urban developments, according to the U.S. National Resources Inventory (*155*, *156*). Spatial patterns of conversion rates are displayed in fig. S8. Many of the highest rates are concentrated around urban centers, with additional hotspots in areas of rapid exurban development. Some agricultural hotspots are also detected, such as in the Central Valley of California. Additional hotspots are scattered across semi-arid regions of the West, with some experiencing wholesale loss of forest cover (see 2010 forest cover extent mapped in fig. S8).

We estimated pre-conversion biomass stocks (B_{preD}) based on the North American Carbon Program Aboveground Biomass and Carbon Baseline Dataset (NBCD) (*140*). This dataset reports CONUS aboveground biomass at a 30 m resolution for the year 2000. We sampled the NBCD map for all pixels that the NAFD product indicated as disturbed from 2001 to 2010. The ratio of total tree dry biomass to aboveground tree dry biomass is assumed to be 1.25 (B_{scale}) based on the component ratios reported in Jenkins *et al.* [Table 2 in (*130*)].

We assume only partial emission of woody biomass stocks, because a portion of the harvested carbon is retained in wood products and a portion of the unharvested carbon decays too slowly to be considered contemporary. We assume that, in conjunction with the deforestation process, 8% of woody biomass is harvested as roundwood (*157*) and 31% of that roundwood is emitted as logging residues (*122*). In addition, 56% of woody biomass from the remaining unharvested "slash" biomass pool is lost within the 20-year committed emissions accounting horizon (*132*). Combining these assumptions, we prescribe an emissions factor (*EF*) of 54%.

We applied a 50% deduction to mitigation benefits of avoided conversion of conifer-dominated forests to account for the direct warming effect of these dark trees (*88*), which have been shown to offset the climate effects of forest carbon sequestration because of changes in albedo (*89, 158*). This deduction was applied to the following forest type groups: White/Red/Jack Pine, Spruce/Fir, Longleaf/Slash Pine, Pinyon/Juniper, Douglas-fir, Ponderosa Pine, Western White Pine, Fir/Spruce/Mountain Hemlock, Lodgepole Pine, Hemlock/Sitka Spruce, Western Larch, Redwood, Other Western Softwood, California Mixed Conifer, Exotic Softwoods. While soil carbon emissions are expected when temperate forests are converted to cropland, a minority of forest conversion is to cropland, so we do not consider that pool in our calculations. The overall effects of conversion of natural systems to residential development is unclear, but may lead to increases in soil carbon (*159, 160*).

Figure S9 illustrates the relative emissions from areas with high risk of conversion. We assumed future conversion was more likely in areas with high rates of historic conversion nearby. For the purposes of mapping, we identified the 98 ha (1089 pixel) patches that experienced the top 25% rates of forest conversion during the historic period of 1986 to 2000 (>1.06%) as areas with high risk of conversion. We estimated the carbon emissions that would occur if any individual 30 m x 30 m location that was forested in the year 2010 was to be converted to non-forest. We excluded protected areas as defined by Gap status 1, 2, or 3 in the Protected Area Database for the U.S. (*161*).

Harvest Rate Results
CONUS mean annual forest clearing for all clearing types (1986 to 2000): 2.369 Mha yr^{-1}
CONUS mean annual forest clearing for all clearing types (2001 to 2010): 2.883 Mha yr^{-1}
Results for specific forest types and regions are shown in tables S7 and S8.

Conversion Rate Results
Average annual cleared area that was converted (for area cleared 1986 to 2000): 380,417 ha yr^{-1}
Percentage of 1986 to 2000 clearing that was interpreted as conversion: 16%
Variations by forest type and region are substantial (tables S9 and S10).

Pre-Disturbance Biomass Results
Tables S11 and S12 report pre-disturbance biomass by forest type and region. Multiply by 0.47 to calculate the mass of carbon.

Carbon Emissions Results
Total CONUS carbon emissions from forest conversion: 14.37 Tg C yr^{-1}
Variations by forest type and region are substantial (table S13).

Albedo-Adjusted Carbon Emissions Equivalent Results
Albedo-adjusted CONUS carbon emissions from forest conversion: 10.46 Tg C yr^{-1} (table S14).

Comparison with other studies
We have found three reports to which our estimates can be formally compared. A recent study by Harris *et al.* (*162*) reported carbon emissions from CONUS deforestation equaling 6 ±1 Tg C yr^{-1}, over 0.1 Mha yr^{-1} of converted land. Their assessment is based on land previously classified as forested becoming re-classified as agriculture, barren land, or settlement in the NLCD during the period 2006 to 2011. Their carbon emissions estimate is about half of that reported here, possibly due to 1) a more conservative approach of requiring reclassification to another land use type, 2) a different forest biomass data product, or 3) a time period of analysis that was coincident with the pronounced economic downturn from 2008 to 2011 when harvesting and land clearing for the housing sector slowed. Another study that also used NLCD data estimated 729 Tg C lost from deforestation across CONUS from 1992 to 2001, equating to 81 Tg C yr^{-1} (*163*). The large discrepancy with what we report is likely related to their much larger estimate of the area deforested annually, 9.3 Mha yr^{-1}, or 24 times the rate we estimate, as well as their assumptions regarding large soil carbon emissions. Lastly, the most recent U.S. Environmental Protection Agency (EPA) Greenhouse Gas Inventory Report (*78*) reports deforestation emissions of about 22 Tg C yr^{-1} for 2011 to 2015. Eighty percent of the emissions (17.6 Tg C yr^{-1}) are derived from loss of live biomass carbon stocks, which is the only component of forest carbon considered in this study's estimate. Conversions to settlements and grasslands dominate, contributing 12.2 and 8.8 Tg C yr^{-1} respectively, with a modest contribution (0.9 Tg C yr^{-1}) from forest conversion to croplands.

Uncertainty for avoided forest conversion
We estimated 13% uncertainty of avoided forest conversion extent by combining the results of NAFD error matrixes for the 6 sites reported in (*164*) and calculated 95% CI following good practice guidelines for area-weighted accuracy from (*165*). We estimated 39% uncertainty of avoided forest conversion flux by combining (*72*) 5% uncertainty from a comparison of five estimates of CONUS carbon stocks (*140*, *166–169*) reviewed in (*170*) and 38% decay uncertainty from the sixteen CONUS studies reviewed in (*132*).

Marginal abatement costs for avoided forest conversion
We were unable to locate studies for the U.S. that assess marginal abatement costs of avoided forest conversion. Both Golub *et al.* (*99*) and Lubowski *et al.* (*171*) estimate marginal abatement costs for both avoided forest conversion and reforestation combined. Because Lubowski *et al.* (*171*) use a 250-yr time horizon while Golub *et al.* (*99*) use a 20-yr horizon in their abatement estimates, we use the latter study. Note that Lubowski *et al.*'s (*171*) abatement cost estimates are

an order of magnitude lower than Golub *et al.*'s (*99*). We use a best-fit (polynomial; $R^2 = 0.99$) function for Golub *et al.*'s (*99*) data for their "extensive margin" (avoided conversion and reforestation) to interpolate the MAC for the abatement levels from avoided forest conversion estimated in our biophysical analysis.

Urban Reforestation

We estimated the potential for an increase in forest cover in urban areas to sequester carbon. We first estimated the current forest cover in U.S. cities and then estimated the potential to increase this cover.

Estimate current forest cover in U.S. cities

We used ESRI's ARCMAP 10.2 to extract forest cover within municipal boundaries (*48*) (fig. S10) from the University of Maryland 30 m spatial resolution (UMd) continuous percent forest cover (%FC) for the year 2010 (*172*). The small percentage of areas lacking 2010 data (probably due to cloud cover) was filled by inserting older (2005) UMd %FC data.

Because 30 m imagery may not detect many street trees, which in some cities account for a high percentage of all FC [e.g. (*173*)], we calibrated the UMd %FC data using National Agricultural Inventory Program (NAIP) 1 m resolution data (*174*), resampled to 2 m to exclude small shrubs and other non-tree vegetation. The NAIP data was from 2012 to 2015, depending on city (*50*). NAIP %FC is strongly correlated ($R^2 = 0.92$) with %FC as estimated using an even higher-resolution (0.15 to 2 m), image analysis-based forest cover assessment (*175*) in a sample of 15 U.S. cities (*50*). Because NAIP data is not available for all U.S. urban areas, we estimate %FC in all urban areas using UMd 2010 %FC, adjusted by the relationship between UMd 2010 %FC and NAIP-calibrated %FC (fig. S11; $R^2 = 0.53$) using data for 27 cities from Kroeger *et al.* (*50*). We estimate city-wide average % tree cover for all U.S. urban areas (N = 3,535) by dividing total forest cover by total municipal land area.

Potential for adding urban tree cover by 2025

We estimate current potential %FC increase for all U.S. municipalities as the mean (and 95% CI) of estimates of total potential %FC increases in the 27 cities from Kroeger *et al.* (*50*) (mean: 17.7%, 95% CI: 14.9 to 20.6%, expressed as share of city land area). Kroeger *et al.* (*50*) identify the potential street tree planting area in each street segment in each of the 27 cities as the difference between current NAIP % tree cover (TC) in a 16 m-wide buffer around the segment centerline and the city's 95th percentile NAIP segment %TC, multiplied by buffer area. In each city, they identify potential patch planting sites outside of street segments by first excluding impervious, agriculture, water and wetland areas and current NAIP tree cover within 2010 U.S. Census city boundaries. To exclude non-impervious areas likely not available for tree planting, Kroeger *et al.* (*50*) extract Normalized Difference Vegetation Index (NDVI) data, adjust NDVI thresholds for each city to account for image variation across the U.S., and use a combination of NDVI and a city-specific entropy-based texture analysis to remove smooth-texture NDVI areas, which correlate with golf-courses, sports fields and lawns. They exclude patches <100 m^2 in size, and, to avoid potential biodiversity conflicts, in non-forest biomes [identified using (*176*)] patches ≥20 ha or >50% in natural land cover.

We use this potential %FC increase and city land area to calculate, for each city, the potential increase in forest cover (in hectares). We assign this potential %FC increase to patch and street plantings using the mean share of potential street (0.46) and patch (0.54) planting area in the 27 cities from Kroeger *et al.* (*50*). To avoid potential biodiversity conflicts or water constraints, we conservatively assume that in cities not located in forest biomes (*176*), only street tree planting will occur. This biases our estimates of available planting area downward since biodiversity conflicts are unlikely for most patches given the already highly altered ecology of most private yards and public parks, and because tree irrigation requirements in arid cities can be minimized through the use of species tolerant of low soil moisture [e.g. (*177*)].

We assume that the total area of potentially reforestable patches within existing urban boundaries will decline over time as a result of gradual increase in impervious surface area (ISA). We assume that increases in ISA will negatively impact the potential for patch tree plantings, but not street tree plantings, because street trees are, by definition, incorporated into areas with primarily impervious surface cover. We note that avoided forest loss from urban growth is included in the analysis for the avoided forest conversion pathway, and so is intentionally excluded here. Bounoua *et al.* (*178*) give projected 2001 to 2020 population and %ISA (as percent of urban area) change for 12 urban areas in the U.S. Assuming average annual %ISA change during their 20-year period is the same, on average, as during our 2017 to 2025 projection period for all U.S. municipalities, we scale their average %ISA change to 2017 to 2025 after excluding the desert cities in their sample (because large-scale patch tree planting would likely not be an option in desert cities).

We multiply the resulting estimated total 2017 to 2025 %ISA change (5.1%) by the average share (0.86) of %ISA increase in Nowak and Greenfield's (*175*) 20 study cities that comes at the expense of a reduction in non-forest covers (grass/herb, soil), to derive the estimated reduction in potentially reforestable area. This yields an estimated average ISA increase of 4.4% (as share of urban land area) during 2017 to 2025 that will come at the expense of reforestable area. We subtract this percentage from the previously estimated average share of U.S. municipal area currently potentially available for urban tree patch plantings (9.5% of urban land area, 95% CI: 8.0 to 11.1%, calculated as 54% of total urban area potentially available for reforestation) to derive our estimates of potentially reforestable city area in 2025, calculated separately for cities in forest (13.3%, 95% CI: 10.5 to 16.2%) and non-forest biomes (8.2%, 95% CI: 6.9 to 9.5%).

We estimate potential absolute increase in tree cover in each of the 3,535 U.S. municipalities (*48*) as the product of the estimated potential %FC increase in a city in 2025 and its terrestrial area (*48*).

Because urban tree planting and maintenance costs are reported per tree (see below), we made estimates of the number of trees for a given canopy area, with separate estimates for street trees and patch trees. These estimates are based on mean growing space requirements for trees without lateral restrictions (*50*). We conservatively assume an average stem diameter at breast height (dbh) at age 40 of 45 cm [63 to 85% of the estimated dbh of 40-year-old urban camphor trees, red oak, and green ash, respectively (*179–181*)] and calculate the estimated average crown radius (5.0 m) as the mean of the crown radii derived using Pretzsch *et al.'s* (*182*) pooled 95th percentile crown radius-stem diameter allometric equations for large (1 equation) and medium size (3

equations) urban trees with dbh = 45 cm. We calculate number of street trees planted ha^{-1} of canopy cover at maturity by dividing canopy area ha^{-1} (10,000 m^2) of street trees by the estimated mean crown area (77.1 m^2). For patch tree plantings, we note that urban plantings are typically not as dense or uniform as reforestation plantings. We assume that reforestation patches have the same sequestration rate used in our reforestation pathway (1.33 Mg C ha^{-1} yr^{-1}). Because this rate is equivalent to 61% of the mean net C sequestration rate of urban tree canopy cover calculated from Nowak *et al.* [2.18 Mg C ha^{-1} of canopy cover yr^{-1} from (*183*), see next sub-section], we calculate the number of patch trees planted as 0.61 of the potential patch reforestation area, divided by mean crown area at maturity (77.1 m^2).

Given the estimated total potential increase in U.S. urban tree cover in 2025 (2.65 to 4.04 Mha), this requires the planting of an estimated 296 to 435 million trees in total. This is equivalent to approximately 7 to 11% of the current estimated total U.S. urban tree population of approximately four billion (*184*). For comparison, annual forestry-associated tree seedling production in the U.S. in 2011 to 2012 was 1.2 billion seedlings yr^{-1} with approximately 1Mha yr^{-1} planted (*185*). This does not include urban tree planting, which is much smaller in scale. Urban plantings generally require larger growing stock (i.e., trees rather than seedlings or saplings) than wildland plantings to better withstand accidental damage and vandalism (*186*). Note that our cost estimates assume replacement of all tree mortality due to impacts from disease, pest, or other events. We therefore do not correct numbers of planted trees for annual survivorship.

Large-scale urban tree cover increases therefore will require a substantial increase in nursery capacity and likely contract-growing to produce the required output, especially of larger stock sizes. We assume that implementation is spread evenly over 9 years (2017 to 2025), with 33 to 48 million new urban trees planted annually (i.e., above and beyond mortality replacement), or approximately 9,300 to 13,400 on average per municipality per year.

C sequestration through urban reforestation

For street trees, we calculate the estimated total net C sequestration from U.S. urban reforestation in a given year as the product of the cumulative canopy cover (number of trees planted by that year, starting in 2017, multiplied by the estimated average canopy area per tree in m^2) and the average annual street tree C sequestration rate. We calculate the street tree C sequestration rate as the population-weighted (*187*) average annual whole tree (shoot and roots), net C sequestration rate by urban trees in the 50 U.S. states and the District of Columbia [0.218 kg C m^{-2} tree cover yr^{-1}, 95% CI: 0.202 to 0.234 kg C m^{-2} tree cover yr^{-1}; (*183*)]. Because Nowak *et al.* (*183*) do not present uncertainty for their state-level average net sequestration rates, we constructed the 95% CI for net sequestration rates by scaling the 95% CI for their gross sequestration estimate for all urban forests in the U.S., which we calculated using their estimate (25.6 Tg C yr^{-1}) and with a 95% CI of 23.7 to 27.4 Tg C yr^{-1} for total annual gross sequestration by urban forests in the 50 states. This net sequestration rate reflects whole tree (shoot and roots) carbon sequestration minus carbon emissions from decomposing dead trees. We reduce this rate by 5%, which is a conservative estimate of the share of annual whole-tree net carbon sequestration by urban trees that is offset by emissions from urban tree planting and maintenance activities [Fig. 4 in (*67*); (*188*)]. It does not reflect the avoided carbon emissions from reduced residential energy use due to the shading of structures or the reduction of ambient air temperature by trees (*50*, *189*), which

can exceed net sequestration by urban trees depending on location (*67*, *190*, *191*). It also does not reflect any soil C accumulation that may result from reforestation.
For patch tree plantings, we calculate the estimated total net C sequestration from U.S. urban reforestation in a given year as the product of the hectares of patches reforested and the estimated mean annual sequestration rate used in our reforestation pathway (1.33 Mg C ha^{-1} yr^{-1}). This rate is lower than for street trees because it represents sequestration per hectare of patch reforestation area—which does not always have full canopy cover—rather than sequestration per hectare of canopy cover.

Our estimates of annual net carbon sequestration by newly planted urban trees are likely conservative for two reasons. First, they do not include potential soil carbon sequestration for street trees. Second, they are based on net sequestration rates of existing urban trees representing a mix of young, mature, and old trees, not newly planted urban trees. Given that mean life expectancy of urban street trees in the U.S. may be around 11 to 28 years (*192*) and that of non-street trees is perhaps 40 years under moderate maintenance levels (*67*), the average annual net sequestration rates from the literature we used in our analysis represent averages for trees ranging from a few years to many decades. Given that stand-level, whole ecosystem carbon sequestration declines with stand age (*193*) and that our cost estimates assume replacement of any tree mortality, use of those average literature sequestration rates biases our sequestration estimates downward.

Results for urban reforestation
We estimate the total potential for urban forest cover increase in 2025 at 3.34 Mha (95% CI: 2.6 to 4.04 Mha), and total annual net C sequestration (reduced by planting and maintenance-related C emissions, but not adjusted for avoided C emissions from reduced building energy use) in 2025 at 23 Tg CO_2e yr^{-1} [(95% CI: 19 to 30 Tg CO_2e yr^{-1}); table S15].

For comparison, existing U.S. urban forests sequester an estimated 18.9 Tg C yr^{-1} net (based on the estimate in Nowak *et al.* (*183*) of 25.6 Tg C yr^{-1} gross sequestration and their reported 0.74 net:gross ratio).

The average estimated cost (street and patch plantings combined) per Mg CO_2 avoided by 2025 is USD 602 (95% CI: USD 452 to 735), which is within the range of estimated costs per Mg CO_2 of four urban reforestation projects in Colorado, if avoided C emissions from reduced energy use resulting from tree shading are excluded (*191*).

An estimated average annual 0.2 Tg CO_2 (0.1 to 0.5 Tg CO_2) could be sequestered by 2025 at USD ≤100 Mg CO_2^{-1} table S16), while zero could be abated at USD ≤10 Mg CO_2^{-1}.

Uncertainty for urban reforestation
Estimates of potential %FC increase are based on the estimated average feasible %FC increase from the 27 U.S. cities in Kroeger *et al.* (*50*), adjusted using three scenarios of future loss of potentially plantable areas due to urban in-fill development. These scenarios were developed from published estimates of rates of ISA increase and conversion of non-tree vegetative covers and bare soil to ISA. The estimate of currently available planting area from Kroger *et al.* (*50*) represents an ambitious reforestation scenario. Our scenarios are more conservative since we

assume that in non-forest biomes only street trees would be planted. Our rate of assumed %ISA increase is the mean of published estimates of predicted %ISA increases in 9 U.S. cities located in forest or grassland/woodland biomes, and may not be representative of the average rate of %ISA increase across U.S. cities. Our rate of conversion of potentially reforestable (grass, herb, soil covers) sites to ISA is based on a 20-city average rate, which likewise may not be representative of the U.S. average rate of this land cover conversion type.

Nowak *et al.* (*183*) provide the standard error (SE) for their estimate of total annual, whole-tree gross C sequestration by urban forests in all 50 states (US50), which is based on field data across the U.S. Our use of those rates results in conservative net C sequestration estimates (and upward-biased MAC estimates) for two reasons. First, forest stand-level C accumulation exceeds whole-tree C accumulation in forest stands (*193*). Second, since stand-level C sequestration declines with stand age (*193*), the C sequestration rate in new plantings is expected to exceed the average C sequestration rate in existing urban forests–the rate we used in our analysis.

We use the ratio of 2*SE to the mean of the Nowak *et al.* (*183*) US50 total gross C sequestration estimates to construct a 95% CI for our estimate of the average annual net sequestration rate by urban street tree plantings in the U.S., thus assuming that the SEs of grass and net sequestration are proportional to their sequestration ratio. Given that Nowak *et al.* (*183*) state that net C sequestration equals 74% of gross sequestration, we do not think this assumption increases the uncertainty in our estimates.

For patch plantings, we use a mean sequestration rate for reforestation of natural ecosystems (see reforestation pathway).

Marginal abatement costs for urban reforestation

We calculate average annual urban reforestation costs ha^{-1} for street tree plantings as the average annualized costs ha^{-1} of urban street and patch tree planting in the 27 U.S. cities analyzed in Kroeger *et al.* (*50*). These costs include all planting and maintenance-related tree costs including tree replacement. For patch plantings, we multiply Kroeger *et al.'s* (*50*) cost ha^{-1} by the ratio of our (52%) and their (100%) estimated percent of patch tree canopy cover. We assume that urban reforestation projects would be financed through municipal bonds and increase the above costs by the 2011 to 2016 average of municipal bond interest rates for Aa, A, and Baa rated bonds with 20-year maturity (4.25%) (*194*). We calculate annual urban reforestation costs by multiplying the total areas of street and patch trees, respectively, planted by a given year in 2017-2025 by the average annualized, finance cost-augmented street and patch planting costs ha^{-1}.

We assume that Kroeger *et al.'s* (*50*) 27-city average street and patch tree planting and maintenance costs ha^{-1} of street and patch tree increases are representative of average urban street and patch tree costs in the U.S.

<u>Avoided Grassland Conversion</u>

We used data on the spatial location of previous conversion of grassland and shrubland (<25% tree cover) to cropland (2008 to 2012) to estimate annual emissions from conversion, and then assume that this rate continues (fig. S12). Conversion to other sources (e.g. residential

development) has an ambiguous impact on soil carbon pools, so conversion to land uses other than cropland are not considered.

We considered emissions from biomass and soil carbon. In grasslands, we only consider belowground biomass. In both grasslands and croplands, aboveground biomass is annually harvested, burned, grazed, or decomposed within a few years and therefore is not considered in avoided emissions calculations. In shrublands, we considered carbon in aboveground biomass, belowground biomass, and litter.

For soil carbon flux, we assume the average percent loss of soil organic carbon from the top 1 m to be 28% following conversion to cropland (95% CI: 15 to 41%) (*26*). This estimate, from a recent literature review, is based on the average of studies that compared uncultivated grasslands to paired cultivated soils in the U.S. (N = 18) where soil organic carbon data was available to a depth of 1 m and reflects the committed emissions that occur over time (~20 years) when grassland is converted to cropland. We applied this loss rate to the amount of soil carbon as estimated to a depth of 1 m by the SoilGrids250m v.2 soil carbon database to get a national map of potential soil carbon emissions (*195*). SoilGrids250m contains globally gridded, 250 m resolution maps of soil organic carbon that were generated using machine learning techniques and explicitly considers land cover as reported by the 2010 CCI Land Cover Product (*196*) when determining the soil organic carbon stock of a given pixel. Due to temporal and thematic disagreement between our land use change layer (*25*) and the classification scheme used to generate SoilGrids250m (*195*), we limited our analysis to pixels that are classified as grasslands or shrublands by both datasets to best reflect potential C emissions from conversion of these land cover types to cropland.

We assume all perennial root biomass is lost when converted to an annual cropping system. We used root biomass data from U.S. grasslands in Mokany *et al.* (*52*) to develop a predictive model for root biomass in grassland based on mean annual temperature (MAT), mean annual precipitation (MAP), and their interaction. We set the minimum root biomass in grasslands to 1.15 Mg biomass ha^{-1} (*197*) to avoid unrealistically low model predictions in the most hot and dry places in the country. This is based on values observed on grazed grassland at the hot and dry Jornada experimental station in southern New Mexico [MAT: 15 °C, MAP: 250 mm; (*198*)]. We assumed that shrubland contained 10 Mg C ha^{-1} in biomass (*199, 200*).

We then used an analysis of the spatial location of conversion to cropland from Lark *et al.* (*25*) to estimate the committed emissions from soil carbon and root biomass for the years 2008 to 2012. The conversion data is based on analysis of the USDA Cropland Data Layer combined with additional processing and data to correct for biases, account for cropland rotations, and improve identification of conversion locations (*25*).

Lark *et al.* (*25*) estimate a rate of grassland loss of 0.69 Mha per year. Our analyses found an average rate of 34.2 Mg C ha^{-1} of soil carbon loss, and 8.3 Mg C ha^{-1} biomass loss on these lands, resulting in a total potential avoided loss of 29.3 Tg C yr^{-1}.

Uncertainty for avoided grassland conversion
We estimated uncertainty for area and for flux per hectare. For area, we used the temporal variation, based on four years of conversion from 2008 to 2012, which found a 95% CI of ±13%. For flux per hectare we calculated uncertainty in the percentage of soil carbon lost and used the model uncertainty for root biomass.

Marginal abatement costs for avoided grassland conversion
We used county-level average per hectare payments from the CRP to estimate the spatial variation in the cost of avoiding conversion of grassland and shrubland (*201*). We averaged county level per-hectare payments from the last three years for which data were available, U.S. federal fiscal years 2007-2009. Before averaging, we adjusted payments to 2015USD. We created MAC curves based on this spatial variation in cost and the spatial variation in soil carbon and root biomass that we mapped, as described above (fig. S13).

<u>Cover Crops</u>
Within the cover crops pathway, we focus on cover crops as a practice that has consistent positive recorded effects on carbon sequestration. Cover crops are defined as the cultivation of additional crops in fallow periods between main crops (*52*). To estimate the maximum potential extent of cover crops, we assume that they could be implemented on all five major field crops in the U.S., which cover 97.5 Mha of land (*202*). The most recent USDA Census of Agriculture (*203*) reported the use of cover crops on 4.2 Mha. Therefore, we quantify the benefit of expanding this practice by 88.2 Mha. It is possible for cover crops to be used on all of these acres, although interseeding (planting of the cover crop before harvest of the main crop) may be the best practice for establishing cover crops in northern latitudes in order to ensure successful establishment before winter (*204*).

Poeplau and Don (*51*) is the most comprehensive and rigorous meta-analysis of carbon sequestration from cover crops to date, and finds an average effect of 0.32 Mg C ha^{-1} yr^{-1} that is consistent across crop choice, tillage regime, and climate. It is also similar to other prior estimates (e.g. Eagle *et al.'s* (*6*) estimate of 0.37 Mg C ha^{-1} yr^{-1}). Poeplau and Don's estimate is based on field observations of cover crop implementation for up to 54 years. Their model suggests that a new equilibrium is reached after 155 years. We assume that their sequestration rate applies for at least 50 years.

In total, we find a potential of increasing mitigation from cover crops by 109.5 Tg CO_2e yr^{-1}. We do not include no-till as a carbon sequestration practice. Although several studies report carbon sequestration benefits of no-till [e.g. (*205*)], more recent research has questioned this finding, suggesting that no-till fields have less carbon than conventional fields at depths below 25-30 cm, largely offsetting the increased carbon at shallower depths (*206*–*210*). Another sampling issue is that failure to correct for changes in soil bulk density in no-till soils has led to overestimates of the benefit of no-till (*209*). No-till has also been shown to increase N_2O emissions for a decade after it is initiated (*211*, *212*). Therefore, any potential benefits of no-till would likely require continuous implementation of the practice for over a decade. However, less than 14% of no-till fields in the corn belt have been in continuous no-till for at least 6 years (*213*). In total, while it is possible that cropping systems including reduced or zero tillage do

have potential to sequester carbon in soils, the current state of knowledge does not enable a reliable estimate, and consequently this practice is not included here.

Uncertainty for cover crops
There is minimal uncertainty in the number of acres planted in the five major crops in the U.S., and we do not consider this as a source of uncertainty. Poeplau and Don (*51*) report a sequestration rate with a 95% CI of 0.16 to 0.48 Mg C ha^{-1} yr^{-1}, which translates to a 95% CI of 53 to 154 Tg CO_2e yr^{-1} for total mitigation potential.

Marginal abatement costs for cover crops
Cover crops can generate a variety of benefits and costs, both internal and external to the farm (*214, 215*). The net effect of these impacts on farm-level profitability is a function of many factors and in a given case may be either negative or positive (*216*), though appropriate selection of cover cropping design can dramatically reduce the likelihood of negative outcomes (*217*). We identified studies that assessed the profitability impact of cover crops for corn, corn-soybean rotation and cotton.

We calculate estimated costs for cover crops on corn as the average of the mean absolute change in profitability per hectare of corn under cover crops reported in the three observations for corn shown in table S17. (We calculate low and high costs the same way from low and high profitability impacts per hectare shown in table S17). We calculate the estimated cost of cover crops on soybean as the change in short-term profitability per hectare reported for the first rotation and for the following rotations (table S17), weighted by 1/9 and 8/9, respectively, to account for the fact that the first rotation cost estimate only applies to 1 out of the 9 years of our analysis period. For cotton, we calculate the cost of cover crops for three different scenarios. For conventional till cotton, we calculate cover crop cost as the reduction in profit from planting the most competitive cover crop under conventional tillage with no cover crop, from data in Cochran *et al.* (*218*). For cotton under no-till, we calculate cost as the reduction in profit from switching from no cover crop to planting the most competitive cover crop [from (*218*)]. For strip-till cotton, we calculate cost based on the profit impact from cover crops under reported by Smith *et al.* (*219*). (For soybeans, we calculate the estimated low and high costs —not provided in the source study—based on the average of the ratios of the percent difference of the low to the mean (-110%) or the high to the mean (+193%) impacts on profitability, respectively, of the corn and cotton observations shown in table S17. We calculate the estimated profitability impacts of cover crops on the other two major cereal crops (wheat, rice) as the average of the profitability impacts on corn, cotton and soybean. The profit enhancing impact of cover crops on corn or corn and soybean (table S17) is confirmed by three additional studies on cover crop profitability impacts we were able to locate (*220–222*). Except for cotton, were one of the studies we found reported negative impacts of cover crops on profitability (*218*), all other studies report profit increased from planting at least one of the cover crop options the studies examined. Thus, the studies we reviewed support the hypothesis that well-chosen cover crops increase profitability. A survey by the Conservation Technology Information Center and the Sustainable Agriculture Research and Education program (*223*) confirms this assessment, reporting that among respondents who could evaluate how cover crops impacted their profit on average, only 9% indicated that cover crops reduced their profits.

For each crop, we calculate GHG MAC as the estimated change in profitability (USD ha^{-1} yr^{-1}) divided by the average GHG sequestration (Mg CO_2e ha^{-1} yr^{-1}). Total annual abatement potential of cover crops for each crop is derived as the product of average annual abatement potential of cover crops per hectare (0.32 Mg CO_2e) and total crop production area of the crop in 2016 (*202*). For cotton, we calculated the potential application extent as 4.1 Mha [cotton cropland (*202*) reduced by 4.2%, which is the average cropland extent under cover crops (*203*)]. We assumed that the share of cotton currently (2012) under conventional till is equal to the share of all croplands under conventional till [44%; (*203*)], and assigned the cost of applying cover crops on conventional till cotton to 44% of the cotton cropland currently not under cover crops. We assigned the two conservation till (strip and no till, table S17) cover crop cost estimates for cotton to the remaining 56% of cotton cropland currently assumed not under conventional till based on the relative extent of croplands in no till and conservation till [0.56:0.44; (*203*)].

Table S18 shows the estimated MACs and total annual abatement levels for cover crops on the five rows crops included in our analysis. Under mean cost assumptions, abatement costs are negative, and the full potential abatement is achieved at any of the three carbon prices.

Biochar

Biochar is a form of charcoal designed to be incorporated into soils. When applied, it can significantly increase soil carbon, and has potential agronomic benefits (*224*). The potential for biochar to sequester carbon depends on the supply of suitable biomass feedstocks. We constrain our estimate to biochar that can be produced from agricultural residues, at residue removal levels that do not negatively affect soils. Because some agricultural residues are already harvested (e.g. for use as forage or bedding), we only consider the potential for increased harvest of agricultural residues beyond this non-biochar demand for agricultural residue. An estimate of crop residue availability for biochar in 2025 was taken from the Department of Energy’s 2016 Billion Ton Report baseline scenario (*225*), which considers both recoverable field residues from the five major crops and secondary agricultural wastes from the processing of harvested biomass. The scenario projects 144 Tg yr^{-1} of dry biomass from crop residues available in 2025, additional to that already being harvested, at a farmgate cost (i.e. not including the cost of transportation beyond the farm gate) of USD <66 Mg^{-1}.

Estimating Carbon Sequestration

To estimate the long-term carbon sequestration per Mg of dry biomass, we assume the average carbon content of residues is 45% (*226*). We assume that, after the pyrolysis used to create the charcoal from biomass, 50% of the carbon is retained in biochar (*227*). Of that carbon, 97% of it is recalcitrant, meaning that it decomposes very slowly when the biochar is incorporated into the soil. This recalcitrant carbon has a mean residence time in soils estimated at 556 years (*56*). Assuming the recalcitrant fraction decays exponentially, we used the mean residence time to calculate that an average of 81% of char carbon persists for at least 100 years (using the formula $F_{100} = \exp(-100/MRT)$, where F_{100} is the fraction remaining after 100 years and MRT refers to the mean residence time). As such, “long-term” mitigation per unit of dry biomass feedstock is 0.18 Mg C per Mg of dry feedstock. Our estimate is conservative because we do not assume that heat or gases generated during the pyrolysis process are used to offset fossil fuel use. Although this may increase the total emissions abatement potential in some contexts, this is likely to be a minority of cases and this heat is also useful for drying the biomass prior to pyrolysis.

Additionally, we do not include any second order effects of biochar on soil organic matter or emissions of N_2O or CH_4, based on recent meta-analyses showing these effects to be neutral or only weakly beneficial on average (*55*, *56*).

Uncertainty for biochar
To estimate uncertainty in the mitigation per unit of biomass, we conducted a Monte Carlo analysis in R to combine uncertainties across each term in the following equation

$$M = B * F_C * Y_C * R * \exp(-100/MRT)$$

Where M is total mitigation, B is biomass supply (in Tg yr^{-1}), F_C is carbon content of residue feedstocks by mass (in %), Y_C is "carbon yield" (% of feedstock carbon retained in char during pyrolysis), R is the fraction of char carbon that is recalcitrant (in %), and MRT is mean residence time of recalcitrant carbon. The Billion Ton Report (*225*) does not provide internal estimates of uncertainty for biomass supply. To capture the uncertainty in biomass supply from agricultural residues, we combine the 2015 estimate from the Billion Ton Report with five other estimates within the U.S. since 2000 (*228–232*). Mean and standard error estimates for R and MRT are drawn from a recent meta-analysis of 121 data points (*56*). We derived means and confidence intervals for F_C and Y_C based on data points drawn from studies of residue feedstocks (*226*, *233–236*) and pyrolysis yields (*234–237*).

Marginal abatement costs for biochar
Using a sequestration factor of 0.18 Mg C per Mg of dry feedstock, the USD 66 Mg^{-1} of feedstock cost threshold for residues in the Billion Ton Report (*225*) corresponds to a mitigation cost of USD 100 Mg CO_2e^{-1}, excluding capital or operation costs for pyrolysis equipment and value generated by biochar or co-products. To generate an estimate at USD 50 Mg CO_2e^{-1}, we therefore repeat the process using supply curves provided by the 2016 Billion Ton Report at a farmgate cost of USD 33 per Mg. Biochar is not viable at USD 10 Mg CO_2e^{-1}, although research demonstrating agronomic benefits could inspire adoption even in the absence of payments for carbon storage.

To summarize, we estimate total mitigation potential at USD <100 Mg CO_2e^{-1} as

$$144 \text{ Tg dry matter yr}^{-1} * 0.18 \text{ Mg C per Mg dm} = 95 \text{ Tg CO}_2\text{e yr}^{-1}$$

At USD <50 Mg CO_2e^{-1}, the available feedstock, and thus mitigation potential, is much lower

$$13.3 \text{ Tg dm yr}^{-1} * 0.18 \text{ Mg C per Mg dm} = 8.8 \text{ Tg CO}_2\text{e yr}^{-1}$$

<u>Alley Cropping</u>
Alley Cropping is planting rows of trees at wide spacings with a companion crop grown in the alleyways between the rows. Udawatta and Jose (*238*) estimate a maximum potential of alley cropping on 10% of U.S. cropland (15.4 Mha). Based on a literature review of biomass and soil sequestration rates compared to cropping systems without trees, we estimate an average

additional sequestration from alley cropping of 1.45 Mg C ha^{-1} yr^{-1} (*239–245*). This yields a total annual mitigation potential of 82 Tg CO_2 yr^{-1}.

To estimate uncertainty, we estimated the expected range as 7.7 to 80 Mha by taking the maximum value from the literature (*246*) and halving Udawatta and Jose's (*238*) more conservative estimate to set the minimum. For flux uncertainty, we calculated a 95% CI of ±49% based on the range of values observed in the literature. Combined, this yields a range of 35 to 166 Tg CO_2 yr^{-1}.

The MAC curve depends on multiple agronomic factors. Alley cropping can increase total farm productivity if soil moisture availability is sufficient and if tree-based intercropping systems are designed in a locally adapted way and allow the crops to interact in a complementary rather than competitive manner (*247–249*). The economic attractiveness of alley cropping systems relies, to a large extent, on the high value of tree products (often nuts) in addition to the value of the timber at harvest (*250*), but the direct net effect on profits of switching to alley cropping depends on the particulars of the operation (*251*). Alley cropping also indirectly affects profits by enhancing or diversifying a farm enterprise by adding tree products (e.g., nuts and high-value timber, or wood as energy feedstock), by reducing surface water runoff and soil erosion, by altering water table depths, by improving utilization and reducing offsite movement of nutrients, and by modifying the microclimate for improved crop production (*252*, *253*).

Marginal abatement costs for alley cropping
Frey *et al.* [(*254*); see also Mercer et al. (*255*)] is the only study that estimates break-even net revenue per metric ton CO_2 for alley cropping, compared to soybeans with average crop revenue election (ACRE) or fixed direct payment (FDR) payments. They study three alley cropping systems (pecan-soybeans, cottonwood-soybeans, hard hardwoods-soybeans) in the Lower Mississippi Alluvial Valley (LMAV) on moderately marginal lands [land capability class (LCC) 3]. At a 5% discount rate (DR), the economic return of three different alley cropping systems was positive, but varied threefold among the three tree species studied, with none of them competitive with monoculture soybean or corn cropping. On the most marginal lands, cottonwood alley cropping exceeded the economic performance of the monoculture row cropping systems at 5% DR (*255*).

We assume that alley cropping could be carried out on all crop lands (LCC1 thru LCC8). We calculate break even prices for alley cropping for all LCCs by fitting linear functions to the break-even prices (updated to 2015USD) reported in Frey *et al.* (*254*) on LLC3 and LCC5 lands for pecan-soybean, hard hardwoods-soybean and cottonwood-soybean, respectively. We distribute total potential alley cropping area (15.4 Mha) across LCCs using the percent distribution of croplands among LCCs in 2012 [Table 13 in (*256*)]. Our estimated MAC curve for alley cropping is composed of 18 segments (3 break-even prices per LCC, multiplied by 6 LCCs, noting that LCC6, 7, and 8 are lumped since there is very low acreage in the latter two categories). To partition lands within each LCC across the 3 break-even prices, we assume they are evenly distributed (i.e. with 1/3 in of the three cost classes). Across these 18 segments, we find MAC costs span USD -16.5 to 70.9 Mg CO_2e^{-1}.

Our estimates are consistent with other studies in the literature, which also show that the cost-effectiveness of alley cropping varies widely depending on the location and cropping system. For example, for a site in Missouri, Stamps *et al.* (*252*) report an average annual profit increase of USD 98.84 ha^{-1} yr^{-1} over the life of a plantation (including sale of nuts but not timber) with walnut-alfalfa alley cropping (in their case, using wide alleyways with 24.4 m between tree rows) compared to alfalfa mono-cropping. In another example, using the mean annual C sequestration rate of black walnut-soybean alley cropping (1.8 Mg C ha^{-1} yr^{-1} over 25 yrs) and the net carbon loss from soybean mono-cropping (-1.2 Mg C ha^{-1} yr^{-1}) reported in southern Ontario (*257*), this translates into a break-even price of USD 9.0 Mg CO_2e^{-1} — lower than the prices reported in Frey *et al.* for their three tree species on LCC 3 lands, and comparable to Frey *et al.'s* average of the three tree species on LCC 5 lands (*254*).

<u>Cropland Nutrient Management</u>
Current and future nitrogen fertilizer use under BAU was projected to be 11.86 Tg N yr^{-1} (year 2016) and 12.41 Tg N yr^{-1} (year 2025) based on a linear trend from reported data from 1980 through 2010 (fig. S14).
Translating nitrogen fertilizer use into N_2O emissions, we follow Davidson (*258*) in using a total emissions factor of 2.54% for fertilizer N. This is slightly higher than the IPCC central value of emissions factors (2% direct + indirect), but is on the low end of the range of estimates reviewed by Snyder *et al.* (*259*) of 2 to 5%. Total N_2O emissions from fertilizer under BAU are therefore 0.473 Tg N_2O yr^{-1} in 2016 and 0.495 Tg N_2O yr^{-1} in 2025.

Saving in N_2O emissions under maximum mitigation
To model the effect of Improved Nutrient Management, we reviewed the literature on various best management practices (BMPs) for improved management of inorganic N fertilizer. We found adequate support to estimate four different improved practices: 1) reduce whole-field application rate, 2) switch from anhydrous ammonia to urea, 3) improve timing of fertilizer application, 4) use variable application rate within field. Practices 1 and 4 reduce the total N application rate, while practices 2 and 3 reduce the N_2O emissions per unit of N applied. Notably, the studies demonstrating these benefits also demonstrate that they can be achieved without reductions in yields and have significant health benefits due to improved air quality (*260*, *261*). For each of these practices, we estimated the potential reduction in emissions weighted by the area for which the practice is applicable (table S19). For the use of variable rate within field, we assembled literature results including both N_2O reductions and N rate reductions due to this practice, and calculated the average reduction across all studies to be 15% (tables S19 and S20). Finally, we calculated the combined reduction in emissions for all practices, comparing the overall projected emissions under BAU with our conservation scenario that implements multiple fertilizer practices (table S19). We found that all practices together could reduce N fertilizer use to 78% of the BAU total and overall field emissions (i.e., not including upstream) to 67% of BAU (table S19).

Multiplying the potential reduction in emissions by the total projected fertilizer use we find that under BMP in 2025, field N_2O emissions will be 0.333 Tg N_2O yr^{-1}.
The N_2O emissions reduction in 2025 is calculated as the BAU emission (0.495 Tg N_2O yr^{-1}) minus the BMP emission (0.333 Tg N_2O yr^{-1}) for a total of 0.164 Tg N_2O yr^{-1}. To calculate

emissions reduction in carbon dioxide equivalents, we multiply this value by the 100-year global warming potential (GWP) value of 298 (*262*) resulting in 49 Tg CO_2e yr^{-1}.

In addition to direct field emissions, fertilizer production itself is a significant source of "upstream" greenhouse gas emissions, both through CO_2 emitted during ammonia production and excess nitrous oxide emitted during nitric acid production. We calculated these upstream emissions for both the BAU and BMP scenarios, accounting for the differences in upstream emissions across fertilizer type. We note the BMP of switching from anhydrous to urea increases upstream emissions, but that this is more than offset by the downstream benefits calculated above. We used values presented by Snyder *et al*. (*259*) for the most common nitrogen fertilizer formulations to estimate upstream emissions as shown in table S21, substituting the average value for any formulations without a specific emission factor identified. We estimated average upstream emissions for the current mix of N fertilizer formulations to be 4.41 kg CO_2e kg N^{-1} (table S21). Applying this upstream rate to the total projected N fertilizer under the future BAU (12.41 Tg N yr^{-1}) we project upstream BAU emissions in 2025 of 54.78 Tg CO_2e yr^{-1} (table S21).

To estimate the reduction in upstream emissions, we subtract the upstream BMP emissions from the BAU emissions calculated above. The CO_2 emissions per unit of N manufactured under BMP practices in 2025 actually increase compared to the current value because of greater production of urea relative to anhydrous ammonia (table S19), which has a higher average emission factor (table S21). Total upstream emissions using BMP practices in 2025 are calculated by multiplying the total fertilizer use (9.66 Tg N yr^{-1}) by the upstream emissions factor calculated for 2025 (4.62 kg CO_2 kg N^{-1}), resulting in upstream emissions for the BMP of 44.62 Tg CO_2e yr^{-1} (table S21). Upstream emissions reductions are simply the BAU emissions (54.78 Tg CO_2e yr^{-1}) minus the BMP emissions (44.62 Tg CO_2e yr^{-1}), which equals 10.16 Tg CO_2e yr^{-1}. Total emissions reductions are the sum of the in-field (48.4 Tg CO_2e yr^{-1}) and upstream emissions (10.16 Tg CO_2e yr^{-1}), a total of 59 Tg CO_2e yr^{-1}.

Uncertainty for cropland nutrient management

To estimate uncertainty in our result, we simplified the calculation to the following

$$M = F * (EF_{N2O} (1 - r_1 * r_2 * r_3 * r_4) + EF_{CO2} (1 - r_1 * r_4))$$

Where M is overall mitigation, F is BAU fertilizer use in 2025 in Tg N, EF_{N2O} and EF_{CO2} are in-field and upstream emissions per unit of N applied in Tg CO_2e Tg N^{-1}, and $r_{1\text{-}4}$ are the fractions of total emissions that would remain if each of the four practices were implemented on their maximum land area. For example, practice 1 is reduction of application rates across the whole field by 21%, which is an opportunity across 64% of cropland (*6*, *263*); the total reduction achievable through this practice alone would thus be 21% * 64% = 13.5%, so r_1 is thus (1 - 0.21 * 0.64) = 0.865. We approximate EF_{CO2} as constant.

We estimate percentage confidence intervals in each parameter through a survey of literature-reported values for F (*264*), EF_{N2O} (*258*, *259*), EF_{CO2} (*259*), r_2 [switch from anhydrous ammonia to urea, (*265–268*)], and r_3 [improve timing of fertilizer application, (*269–272*)]. We estimated uncertainty in the product of $r_1 * r_4$ (reduce whole-field rate and use variable rate within field)

through literature estimates in potential to reduce rate or improve N-use efficiency (*273–276*). We calculated overall uncertainty bounds through a Monte Carlo analysis conducted in R.

Marginal abatement costs for cropland nutrient management

Ribaudo *et al.* (*277*) find that 53% of all N applied in U.S. cropping systems is applied on fields on which N application (commercial and manure) rates exceed 140% of the N removed with the crop at harvest. In 2006, 66% of all N fertilizer use was on corn (*277*).

N application rate and timing

In 113 field trials of the Adapt-N tool in New York and Iowa that covered four growing seasons, Sela *et al.* (*263*) found that N fertilizer application rates on corn could be reduced on average by 34% and 37%, respectively, compared to grower N fertilizer rate, leading to no statistically significant differences in yields and increasing average grower profits by USD 65 ha^{-1}. Similarly, Smith *et al*. (*205*) estimated that a 20% reduction in N application rates was feasible without severely negative yield impacts, and Millar *et al*. (*278*) estimated that 12% to 15% reductions are possible by shifting from the high to the low end of the profitable N rate range for grain corn.

We calculate a MAC curve for N fertilizer application rate from Sela *et al.*'s (*263*) 113 observations (site × year combinations), where change in grower profit indicates MAC (fig. S15). This curve includes upstream CO_2e emissions as well as both direct and indirect N_2O emissions calculated as described in the supplementary information for the cropland nutrient management pathway. Based on this curve, 72% of observations cost USD 0 Mg CO_2e^{-1} or less, 73% cost USD 10 Mg CO_2e^{-1} or less, 81% cost USD 50 Mg CO_2e^{-1}, and 86% cost 100 Mg CO_2e^{-1} or less. We assume that this MAC curve is representative of all nitrogen fertilizer rate and timing practices.

Variable rate technology

In research sites in Germany, decreasing N fertilizer application rates in low-yielding portions of fields from 150 to 125 kg ha^{-1} resulted in no decrease in yield (*279*). An analysis of corn fields in Iowa estimated the average profit loss for low-yielding portions of fields without VRT at USD 250 ha^{-1} yr^{-1} (*37*). This indicates large economic potential for application of VRT. For example, crop canopy reflectance sensors (*280*) reduced N application rates on Missouri corn by 8% (16 kg ha^{-1}) on average while increasing yield (1%, or 110 kg ha^{-1}), resulting in partial producer profit (value of grain yield minus fertilizer cost) gains of USD 45 ha^{-1}. Schimmelpfenning (*281*) finds that the impact of VRT on profits for U.S. corn producers is positive, though small, increasing both net returns (including overhead) and operating profits by about 1%, for both small (57-162 ha) and large (>486 ha) farms.

We use Scharf *et al.*'s [Table 2 from (*280*)] field-level data on partial profits (value of grain yield minus fertilizer cost; updated to 2015 prices) and N reductions per ha from 55 replicated on-farm VRT demonstrations in Missouri, and account for upstream emission reductions, to construct a partial profit MAC curve for VRT (fig. S16). Based on this curve, 49% of observations cost USD 0 Mg CO_2e^{-1} or less, 53% cost USD 10 Mg CO_2e^{-1} or less, 62% cost USD 50 Mg CO_2e^{-1}, and 67% cost 100 Mg CO_2e^{-1} or less. We assume that this MAC curve is representative of all VRT applications.

While VRT entails fixed costs for equipment purchase, we do not include those costs in our analysis. According to Scharf *et al.* (*280*), on a hypothetical 200 ha farm using sidedress N application, VRT should pay for itself within a few average years. Thus, for reasonable amortization periods, in 2025, the focal year of this analysis, any fixed costs associated with adoption of VRT now (2017) would be minimal.

Switch from anhydrous ammonia to urea
We calculate the cost of switching from anhydrous ammonia to urea fertilizer as the mean difference in the average 2011-2013 prices ((*282*); converted to 2015 dollars) of the two per kg N [anhydrous ammonia, 82.2% N by weight (*283*); urea, 46% N by weight; (*284*)]. Adjusting for differences in upstream emissions (anhydrous ammonia, 2.6 kg CO_2e kg N^{-1}; urea, 3.2 kg CO_2e kg N^{-1}), we calculate the MAC of switching from anhydrous ammonia to urea by dividing the increased cost of urea per kg N (USD 0.28 kg N^{-1}) by the difference in total emissions between the two fertilizers per kg N (reduction of 0.00285 Mg CO_2e kg N^{-1}). This MAC of switching from anhydrous to urea is USD 99 per Mg CO_2e, meaning that all emission reductions from switching to urea can be obtained at USD 100 Mg $CO2e^{-1}$, and zero for USD 10 and USD 50. We assume that these MAC curves are representative to the total area to which each of the respective N management strategies are applicable.

To calculate the percentage of the total feasible mitigation potential that all four N management strategies can achieve if applied in conjunction (59 Tg CO_2e yr^{-1}) on the respective applicable areas, we multiply the percentage of its total abatement that each individual control strategy can achieve at a given MAC by its relative contribution to total feasible abatement from all four N control strategies. We then multiply the weighted joint abatement percentage at USD 10, 50 and 100 Mg CO_2e^{-1} by the aggregate abatement potential of all four N management strategies to derive aggregate abatement at each of our three MAC values. This yields a total estimated abatement of 28.4 Tg CO_2e yr^{-1} at USD 10, 32.1 Tg CO_2e yr^{-1} at USD 50, and 49.9 Tg CO_2e yr^{-1} at USD 100, respectively.

<u>Improved Manure Management</u>
Dairy cattle and hogs account for >85% of the CH_4 emissions from manure in the U.S. (*8*). The USDA estimates the potential for improved manure management on dairy farms with over 300 cows and hog farms with over 825 hogs (*8*). They considered seven types of mitigation options and six baseline manure management practices. Mitigation options vary based on the existing management practice, with between three and six options available depending on the existing management practice. Based on these factors, the USDA estimates MAC curves for manure management in ten U.S. regions (*85*). Overall, they find a mitigation potential of 24 Tg CO_2e yr^{-1} for confined dairy and swine operations at a cost of up to per USD 100 Mg CO_2e.

Uncertainty for improved manure management
The mitigation potential for this pathway is roughly proportional to the number of head of livestock in production. Based on USDA numbers from 2000 to 2017, the variation in the number of head of livestock for dairy cattle and hogs is relatively low with a 95% CI of ±1.3% (*85*). The EPA estimated uncertainty for this pathway, finding that “the largest sources of uncertainty are associated with the estimated CH_4 emission calculations related to animal population data, the estimates of the number of animals using each type of manure management

system, the volatile solids excretion rates, the maximum CH_4 production capacity data, and the CH_4 conversion factors." (*8*). Considering all of these sources, they estimate the overall uncertainty range of 25%. This suggests a potential mitigation range of 18 to 30 Tg CO_2e yr^{-1}.

Marginal abatement costs for improved manure management
Pape *et al.* (*8*) estimate a highly detailed MAC curve for U.S. animal production systems. They find that changes in manure management on confined dairy and swine operations have the potential to mitigate about 24 Tg CO_2e yr^{-1} for break-even prices (2010USD) between USD 1 and USD 100 per Mg CO_2e, or about 50% of total CH_4 emissions related to manure management on livestock operations. We update their MAC from 2010 to 2015 prices, which reduces mitigation potential at USD 100 Mg CO_2e^{-1} to 22 Tg CO_2e yr^{-1}.

<u>Windbreaks</u>
Windbreaks are rows of trees or shrubs planted around fields to protect cropland from wind erosion and control snow accumulation. Pape *et al.* (*8*) identified the potential for planting windbreaks on 0.88 Mha of cropland. This is based on an estimate of 17.6 Mha of cropland that would benefit from windbreaks, and that windbreaks would optimally occupy ~5% of that cropland (*8, 285*). We estimated that windbreaks provide 3.56 Mg C ha^{-1} yr^{-1} additional sequestration in cropland biomass and soils, calculated as the mean of available literature estimates (*286–290*). This yields a maximum mitigation potential of 11.47 Tg CO_2e yr^{-1}.

To generate MAC curves we used region-specific low and high break-even prices for carbon sequestration by windbreaks, based on farm-level installation, annual operation and maintenance costs, and opportunity costs (*216*). We assume windbreaks to be distributed according to the share of total cumulative windbreak acreage installed under the CRP in 2010, considering both the CRP's CP-5A (field windbreak establishment) and CP-16A (shelterbelt establishment) (*291*). These break-even prices do not account for any farm-level benefits of wind breaks such as reduced soil erosion or reduced desiccation of crops. We use the average break-even prices in each region (adjusted to 2015 prices), the average estimated C sequestration rate of windbreaks, and the estimated regional potential increase in windbreak acreage to construct a U.S. MAC curve for windbreaks.

Uncertainty for Windbreaks
To estimate area uncertainty, we estimated a range of 0.45 to 1.7 Mha (i.e. windbreaks on 9 to 34 Mha of cropland) by taking the maximum value from the literature (*238*) and halving Pape *et al.'s* (*8*) more conservative estimate to set the minimum. For flux uncertainty, we estimated a range of ±47%, based on the range of values observed in the literature. Combined, this yields a range of 3 to 30 Tg CO_2e yr^{-1}.

Marginal Abatement Costs for Windbreaks
To generate cost curves, we used farm region-specific means of low and high break-even prices for carbon sequestration by windbreaks, based on farm-level installation, annual operation and maintenance costs and opportunity costs (*216*). Because those prices are based on an assumed C flux rate of windbreaks of 0.94 Mg C ha^{-1} yr^{-1} (*216*), we divide them by 3.77, the ratio of our flux estimate (3.56 Mg C ha^{-1} yr^{-1}) and 0.94 Mg C ha^{-1} yr^{-1}. We assume windbreaks to be distributed among farm regions according to the combined share of total cumulative shelterbelt and field

windbreak acreage installed under the CRP in 2010 (*291*). These break-even prices do not account for any farm-level benefits of wind breaks such as reduced soil erosion or reduced desiccation of crops. We use the average break-even prices in each region (adjusted to 2015 prices), the average estimated C sequestration rate of windbreaks and the estimated regional potential increase in windbreak area to construct a U.S. MAC curve for windbreaks.

<u>Grazing Optimization and Legumes in Pastures</u>
These two NCS increase soil carbon based on 1) grazing optimization on rangeland and planted pastures and 2) sowing legumes in planted pastures. Our estimates are derived directly from a recent global study by Henderson *et al.* (*58*). Grazing optimization prescribes a decrease in stocking rates in areas that are over-grazed and an increase in stocking rates in areas that are under-grazed (fig. S17). Henderson *et al.* found that optimizing forage offtake would result in an increase in carbon storage on 22.1% of the grazing lands in the U.S. (other locations show no effect or a decrease in carbon storage). Implementing grazing optimization on the lands with a carbon benefit would also generate a net increase in forage offtake and livestock production. Henderson *et al.* (*58*) found that sowing legumes would generate net sequestration (after taking into account the increases in N_2O emissions associated with the planted legumes) on 13.2% of planted pastures in the U.S. (fig. S18). Henderson *et al.* (*292*) also conducted an assessment of the MAC of each practice (table S22).

Uncertainty for Grazing Optimization and Legumes in Pastures
The climate impacts of these practices have high spatial variability. We measured uncertainty in these projections by the reported spatial coefficient of variation across the subset of rangeland and pastures where these practices are beneficial (*58*), which translates to a 95% CI of ±227% for grazing optimization and ±137% for legumes in pastures.

Marginal abatement costs for Grazing Optimization and Legumes in Pastures
Henderson *et al.* (*58*) estimated the marginal abatement cost of grazing optimization and planting legumes in pastures. The costs associated with grazing optimization include reduced livestock production in some areas (offset by increased production in other areas). The costs associated with legume seeding include fertilizer, seed, labor, herbicide, and machinery requirements, and were assumed to be incurred every five years to maintain legume cover at roughly 20%.

<u>Grassland Restoration</u>
We assumed that 2.1 Mha were available for restoration of grassland from cropland. This is based on a scenario of 5.1 Mha of total cropland set aside for restoration (e.g., via the federal CRP), with the remaining 3 Mha restored to forests or wetlands (*8*).

We considered sequestration in both root biomass and soil carbon. Aboveground biomass, in both grasslands and croplands, is annually harvested, burned, grazed, or decomposed within a few years and therefore is not considered in our calculations of sequestration from restoration. We did not consider shrubland restoration here, as shrubland restoration is expensive and prone to establishment failure (*293*), and is therefore less likely to occur at large scales, compared to grassland restoration.
For soil carbon sequestration rate, we used a recent meta-analysis of grassland restoration studies (*294*). Kämpf *et al.* (*294*) estimated that soil carbon stocks increased 18% (95% CI: 8 to 28%) in

the upper 20 cm of soil. Since carbon change occurs beyond 20 cm depth (*26, 295*), we applied an exponential curve of the form found to fit global average carbon change data [fig. S8 in (*26*)] such that the average gain in the top 20 cm equaled 18%. Using this exponential extrapolation to 100 cm suggested that soil carbon in the whole 100 cm profile would increase by 12% (95% CI: 6 to 20%) with restoration of grasslands. Further, consistent with IPCC methodologies, we assume that soil carbon restoration occurs linearly over a 20-year period. Using these methodologies, a cropping soil with 125 Mg C ha^{-1} to 100 cm would sequester soil carbon at a rate of 0.73 Mg C ha^{-1} yr^{-1}, which is consistent with previous syntheses [e.g., 0.69 Mg C ha^{-1} yr^{-1}; (*296*)].

We spatially applied the 12% carbon gain value to the 100 cm soil carbon stocks reported in SoilGrids250m (*195*) for locations of recently abandoned cropland that have returned to grassland (*25*) (fig. S19). We limit our analysis to pixels that are classified as croplands by both Lark *et al.* (*25*) and SoilGrids250m (*196*) to quantify the initial soil carbon in areas likely to be restored to grassland. The carbon gain values from each pixel are then divided by 20 to get an annual sequestration rate. Finally, since we do not know the location of future restoration projects, these pixels are averaged to give a nationwide mean annual sequestration rate for grassland restoration. We found an average sequestration rate of 0.82 Mg C ha^{-1} yr^{-1} (95% CI: 0.40 to 2.02).

We assume that perennial root biomass is completely restored over 20 years, and therefore estimate an annual average rate of sequestration of $1/20^{th}$ the expected grassland root biomass in restored grasslands. We used root biomass data for U.S. grassland from Mokany *et al.* (*52*) to develop a predictive model for root biomass in grassland based on mean annual temperature, mean annual precipitation, and their interaction. Note that this is the same model that we used to predict grassland root biomass in the avoided grassland conversion NCS.

We used spatially explicit data on the location of recently abandoned cropland that returned to grassland (*25*) to estimate average per hectare root biomass in restored grasslands, under the assumption that the temperature and precipitation in these areas are representative of future grassland restoration sites. This model predicts an average rate of 0.51 Mg C ha^{-1} yr^{-1} sequestered in root biomass.

Combining both sequestration rates, we find a total sequestration rate of 1.19 Mg Ce ha^{-1} yr^{-1}. Applied to the 2.1 Mha of restorable croplands, we estimate a mitigation potential of 9 Tg CO_2e yr^{-1}.

Uncertainty for grassland restoration

Our area estimate is based on a policy determination of cropland area available for restoration, rather than an empirical estimate of biophysical potential for grassland restoration, and therefore has no associated uncertainty. To estimate uncertainty for flux per hectare, we combined the uncertainty associated with the soil carbon sequestration rate (95% CI: 0.49 to 2.43 Mg C ha^{-1} yr^{-1}) and the root biomass sequestration rate, which suggests an overall 95% CI of 3.1 to 21.4 Tg CO_2e yr^{-1}.

Marginal abatement costs for grassland restoration
We used county-level average per hectare payments from the CRP to estimate the spatial variation in the cost of restoring marginal cropland to grassland (*201*). We averaged county-level per-hectare payments from the last three years for which data were available, U.S. federal fiscal years 2007 to 2009. Before averaging, we adjusted payments to 2015USD. We created MAC curves based on this spatial variation in cost and the spatial variation in soil carbon and root biomass that we mapped, as described above (fig. S20).

Improved Rice Management
Much of the world's rice is grown in standing water, generating anaerobic conditions in the soil, which causes CH_4 and N_2O emissions. Rice paddy CH_4 emissions comprise 10 to 14% of anthropogenic CH_4 emissions (*219*, *297*, *298*). The EPA reports the mitigation potential from improved rice management for North America (*59*). Because over 97% of rice grown in North America is grown in the U.S. (*299*), we adopt those mitigation potential numbers directly. Water management techniques such as alternate wetting and drying (AWD) and midseason drainage (MSD) limit the time rice paddies spend in an anaerobic state and thereby reduce annual CH_4 emissions (*297*). The EPA modeled 26 mitigation scenarios representing AWD and MSD under different management regimes for fertilizer, residue incorporation, and tillage, using the Denitrification-Decomposition (DNDC) model. They report a maximum potential mitigation for North America in 2030 of 3.7 Tg CO_2e yr^{-1}, with potentials of 3.4, 2.8, and 1.5 Tg CO_2e yr^{-1} at USD 100, 50, 30, and 10 per Mg CO_2e, respectively.

Uncertainty for improved rice management
For flux uncertainty, we reviewed the literature on improved water management practices (AWD and MSD) (*297*, *300*–*302*) and found that the 95% CI of the mean percent emission reduction (considering both CH_4 and N_2O, converted to carbon dioxide equivalents) was ±12%. For area uncertainty, we used USDA data on temporal variability in planted rice acreage (*86*). Over ten years (2008 to 2017), the 95% CI was ±8%. We combined these uncertainties (*72*) to estimate an overall uncertainty of 3.2 to 4.2 Tg CO_2e yr^{-1}.

Marginal abatement costs for improved rice management
Our MAC estimates for abating U.S. GHG emissions from rice management are based on the break-even prices for CO_2e for North American rice production reported in US EPA [Table 2-8 in (*59*)]. The latter include both Mexico and the US, but with US rice production in 2016 accounting for 97.6% of all North American rice production [Table 10 in (*202*)], any errors from applying US EPA's (*59*) break-even prices for North American rice production to US rice production will be negligible given the size of reductions analyzed here. Break-even prices assume changes in rice field management that cause no impact on baseline rice production. We update the US EPA (*59*) North American break-even prices to 2015USD, fit a 3rd order polynomial to the ten data points (R^2=0.99; fig. S21) and use that function to estimate annual abatement at our three carbon price points.

Tidal Wetland Restoration
Over one-quarter of the tidal marsh in the U.S. has impaired or disconnected tidal connection with the sea, making these marshes subject to freshwater inundation and turning this formerly robust carbon sink into a potent CH_4 emitter (*30*). We define tidal wetlands to include both

estuarine marshes and mangroves. We first estimated the area of disconnected or impaired marsh with freshwater inundation as the target area for restoration. Using data from eight studies that surveyed 55% of the total tidal marsh area along the eastern coast of the United States, Kroeger *et al.* (*30*) found that, of the 0.97 Mha of marsh surveyed, 0.38 Mha (39%), were landward of a dike, transportation infrastructure, or other tidal restriction (*30*). Of the 0.38 Mha of tidally restricted marsh, an estimated 70% is considered "freshened" due to impaired drainage of fresh water. Freshened marshes have salinity levels below 18 psu (Practical Salinity Unit), allowing an increase in methanogenesis and CH_4 emissions to occur. Therefore, an estimated 27% of tidal marsh area is currently releasing CH_4 at an enhanced rate due to human actions (*30*). We apply this percentage to the current area of both tidal estuarine marsh and mangroves in the U.S. [1.79 Mha, (*303*)] to estimate that 0.48 Mha of tidal marsh in the U.S. is emitting anthropogenic CH_4 and could potentially be restored. Using estimates of CH_4 emissions from natural, unimpaired tidal marsh (0.46 g C m^{-2} yr^{-1}) and CH_4 emission estimates from freshened salt marsh [41.6 g C m^{-2} yr^{-1}, (*30*, *304*, *305*)] suggests an average anthropogenic CH_4 emission rate of 24.7 Mg CO_2e ha^{-1} yr^{-1}. This is based on a sustained global warming potential (GWP) conversion factor of CH_4 to CO_2e of 45 (*306*). The sustained GWP is substantially higher than the standard GWP of 32 over this time frame because the latter is based upon a single pulse of GHG release to the atmosphere. In contrast, the sustained GWP is based upon continuous release of CH_4 over the time frame and thus is substantially more realistic. Thus, we find that 12 Tg CO_2e yr^{-1} can be prevented by the reintroduction of tidal saline flows into impacted tidal marshes. We expect that this may also have some mitigation benefit from increasing carbon sequestration in soils and sediments, but this is poorly quantified and is not included in our estimate.

We note that our estimate omits drained tidal marshes due to lack of information about the extent to which they could be restored. Many drained tidal marshes are developed and thus are unlikely to be restored. However, drained tidal marshes that were cropped have the potential to recover large amounts of soil carbon (*307*). Inclusion of these additional restoration opportunities would reveal even greater potential for tidal marsh restoration than quantified here.

Uncertainty for tidal wetland restoration

To estimate uncertainty in extent, we combine three sources. Kroeger *et al.* (*30*) breaks tidally restricted marsh into two categories: "transportation related restrictions" and "diked and impounded." Across states, these were found to vary by ±62% (95% CI) and ±84% (95% CI), respectively. When summed, these have a CI of 52%. We estimate a 95% CI of ±10% for total tidal marsh extent based on Bridgham *et al.* (*308*). When multiplied, we find an overall uncertainty for the extent of tidal marsh that could be restored to have a 95% CI of ±53%.

To estimate uncertainty in flux, we calculated the 95% CI for CH_4 emissions from freshened (±53%) and restored (±90%) salt marsh (*304*). Combined, the 95% CI for the difference between these two numbers is ±89%. We then calculated the overall 95% CI for the mitigation potential as 0 to 24 Tg CO_2e yr^{-1}. We consider negative mitigation (i.e., an increase in CH_4 emissions) to be implausible because there is no mechanistic basis or empirical evidence for increased salinity to cause sustained increase in CH_4 emissions.

Marginal abatement costs for tidal wetland restoration

Our MAC curve for saltwater marsh restoration (fig. S22) was constructed analogously to that for seagrass restoration (see section *Marginal abatement cost* in the seagrass restoration NCS), except that it was not possible to correct flux intensity for probability of project success due to the very small number of saltwater marsh restoration studies that report on success (N = 4). Our analysis indicates that 60% of potentially feasible abatement is achievable for USD 100 Mg CO_2e^{-1} (fig. S22).

Peatland Restoration

Peatlands are drained in order to convert them to cropland or other uses, which results in removal of live vegetation, decomposition of the stored peat, and subsequent release of carbon dioxide to the atmosphere, and a decrease in CH_4 emissions. Some peatlands are degraded by drainage without wholesale conversion of the natural vegetation, often for forestry. CH_4 emissions are reduced in drained peatlands because draining limits the anaerobic conditions that promote methanogenesis. Restoration of peatlands involves rewetting them, and sometimes also replanting vegetation. Rewetting reduces decomposition and allows carbon to build up in the organic soils (i.e. builds peat), but it also increases CH_4 emissions. We calculated the net GHG benefit from restoring converted and degraded peatlands in CONUS, considering both carbon storage and CH_4 emissions.

We calculated separate estimates for regions of the U.S. based upon IPCC climate zones (tropical moist, warm temperate, cool temperate) (*309*) due to varying climate effects on peatland types and carbon cycling (tables S23 and S24). For states that occupy more than one climate zone, we categorized all the peatlands in that state into the climate zone that contains most of the peatlands in that state. Of particular interest are peatlands in Virginia, North Carolina, and South Carolina (a.k.a. pocosins) because many of these peatlands have been previously degraded (*310*) and evidence suggests that intact pocosin peatlands have low CH_4 emissions (*311–313*).

Peat soils are officially classified in the U.S. as Histosols. Maps of Histosol soils provide an estimate of the historic distribution of peatlands; even when a peatland is converted to a new land use, the soil type is still Histosol unless decomposition over many decades removes the peat. To estimate Histosol area by state, we first used the SSURGO database (*314*), which maps soils at scales ranging from 1:12,000 to 1:63,360. However, there are extensive areas of CONUS that are incomplete or unavailable in SSURGO, and some of these have large areas of peatlands. Therefore, we also used the much coarser STATSGO database, with a scale of 1:250,000, for areas with large data gaps in SSURGO and where STATSGO gave higher estimates for area of peatlands than SSURGO (*315*). Our final estimate was 10.3 Mha of Histosols in CONUS.

We are unaware of any previous direct estimate of the total area of restorable peatlands. To estimate this area, we used the area of intact peatlands defined as areas with Histosol soils that are also mapped as wetlands by the National Wetlands Inventory (*316*), which uses aerial imagery to identify extant wetlands. We consider the total area of restorable peatlands as the Histosol area minus the area of remaining peatlands. The area of forested and non-forested peatlands were taken from the USDA-FS Forest Inventory and Analysis database (*167*).

We estimated subsets of the total area of disturbed peatlands: those that were converted to crops, those that were converted to pasture, and those considered disturbed but still retaining semi-

natural vegetation. This latter category of disturbed peatlands have often been drained for commercial forestry operations. We obtained the area of former peatlands that are currently in crops and pasture from U.S. EPA (*309*).

In order to differentiate between cool and warm temperate disturbed peatlands, we first calculated a separate estimate of restorable Histosols in the southeastern U.S. (AL, FL, GA, LA, MS, NC, SC, VA) because this is where the large majority of warm temperate peatlands occur in the U.S. Histosols were identified with SSURGO. Histosol polygons were then overlaid with areas categorized as agriculture or non-wetland with the National Land Cover Database (*317*) to find the area of disturbed warm temperate peatlands. Finally, the area of disturbed cool temperate Histosols was estimated by subtracting the area of southeastern disturbed Histosols from the total for CONUS. Disturbed Histosols in warm temperate states outside of the southeast would be lumped in with cool temperate Histosols by this calculation, but we expect this area to be small.

To calculate maximum potential restoration extent, we assume that all restorable peatlands in CONUS could be restored. We note that this only requires the restoration of 0.7 Mha of cropland, a small portion of the 5.1 Mha of cropland restoration we assume across all NCS. Food production is safeguarded because restoring 5.1 Mha of cropland is equivalent to setting aside the same amount of land that was retired via the CRP in 2007, at its peak enrollment (*8*).

The peatland areas calculated above were then multiplied by the relevant sequestration/emission rates, broken out by IPCC climate zone when possible, to determine a carbon budget for peatland restoration in CONUS. The carbon budget (including changes in soil carbon and CH_4 emissions) depends on the converted use from which the peatland is being restored, which included pasture, cropland, horticulture and other degraded (including forestry). Rates for pocosins were calculated separately using a site-specific study (*318*). Rates of carbon dioxide emissions from currently degraded peatlands were taken from USEPA (*309*). Net carbon accumulation/loss rates for rewetted Histosols were taken from the IPCC Wetland Supplement (*305*); we used rates for “rich” Histosols for areas in cropland and for “poor” Histosols for areas converted to other uses. The rate of decrease in dissolved organic carbon export upon rewetting was also taken from the IPCC Wetland Supplement (*305*).

We assumed that only restored forested peatlands would produce substantial annual increases in live standing stocks of plant biomass, assuming that other plant functional types would relatively rapidly reach a steady-state standing stock. We estimated the increase in live biomass due to rewetting by assuming that the proportion of forested peatlands in restored peatlands would reflect the proportion of forested peatlands observed in intact peatlands today. To arrive at this estimate, we multiplied the area of all restorable peatlands by the percentage of forested/non-forested intact peatlands. The rate of biomass accumulation in restored forested peatlands was assumed to be 1.83 Tg CO_2e m^{-2} yr^{-1} (*308*).

We estimated the change in CH_4 emissions from a drained to a rewetted state. Emissions from drained Histosols were taken from the IPCC Wetland Supplement (*305*). Because of limited CH_4 emission data from rewetted peatlands in the U.S., we assumed that restored wetlands have similar CH_4 emissions to natural U.S. wetlands in the same climatic zone. Bridgham *et al.* (*308*) summarizes all known studies of CH_4 emissions from peatlands in CONUS at that time. We

updated that summary to include 79 studies and we used the geometric mean flux broken out by climatic zone. The IPCC Wetland Supplement (*305*) gives 2.3 times higher CH_4 fluxes from temperate rewetted rich Histosols than from poor Histosols, which the natural wetland emission dataset (*308*) does not capture because many of the published results do not allow a discrimination between "poor" and "rich" peatlands. Therefore, we estimated CH_4 emissions from restored, temperate-zone cropped Histosols from the IPCC Wetland Supplement (*305*). We converted CH_4 emissions to CO_2 equivalents based on a 100-year sustained GWP of 45 (*306*).

Uncertainty for peatland restoration
Most of our sources for sequestration/emission rates provide uncertainty calculated with IPCC Approach 2 (i.e., Monte Carlo simulations) (*72*) (table S25). CH_4 emission factors used the variation of the reported geometric mean from 79 peatland studies as described above. Uncertainties for soil carbon dioxide emissions from Histosols in agriculture, grasslands, and horticulture include uncertainty for both emission factors and area in the published estimates. However, in the other estimates we use our derived areas, as described above, which did not provide uncertainty estimates. Therefore, the calculated uncertainty in these cases only includes emission factors and will underestimate the true uncertainty. The uncertainties were combined by their weighted sums (*72*) to provide an overall 95% CI of -46% to 26%.

Marginal abatement costs for peatland restoration
To generate the MAC curve for peatland restoration, we combined the regional estimates of per hectare mitigation potential described above with state-level estimates of restorable peatland and county-level estimates of wetland restoration costs (*319*), aggregated to the state level.

<u>Avoided Seagrass Loss</u>
Mangroves, tidal marsh, and seagrasses are all being lost globally, contributing to blue carbon emissions (*320*). Of these three types of tidal wetlands, only seagrasses have meaningful rates of ongoing avoidable conversion in the continental U.S. Due to their protected status, the relatively small losses of mangroves and tidal marsh are due to sea level rise and sedimentation and cannot be avoided by improved management in the near term (*17*). Therefore, the climate change mitigation potential of avoiding tidal wetland loss is based on recent seagrass loss that is predominantly caused by poor water quality from various land-use practices (*70*).

We determined an annual loss rate for seagrasses in the U.S. (0.02 Mha yr^{-1}) by applying the mean loss rate of 1.5% (*61*) to the estimated 1.44 Mha of remaining seagrass (*17, 321*). Assuming 50% of the carbon stocks (89 Mg C ha^{-1}) contained in disappearing seagrass beds are lost to the atmosphere upon conversion (*62*), we calculated a maximum mitigation potential of avoiding the loss of seagrasses in the U.S. of 6.5 Tg CO_2e yr^{-1}.

Uncertainty for avoided seagrass loss
We use Pendleton *et al.* (*62*) to estimate a 95% CI of ±60% for avoided per hectare emissions. For their 1.5% decline per year estimate, Waycott *et al.* (*61*) report a standard error of 1.1, which translates to a 95% CI of 0 to 3.7% (we truncate the range at zero, because there is no empirical support for a net increase in seagrass area). We combine these uncertainties to calculate an overall 95% CI of 2.5 to 10.6 Tg CO_2e yr^{-1}.

Marginal abatement costs for avoided seagrass loss

Because nutrient pollution (primarily N, but also P) is the dominant driver of seagrass loss (*17*, *322*), the cost of avoided seagrass loss is the cost of reducing N loading into coastal waters though improved fertilizer application, reduced N export from concentrated animal production facilities and decentralized (septic) and centralized (municipal) wastewater treatment facilities (*113*, *322*), and reduced atmospheric loading through fossil fuel power plant N emission controls. We note that reductions in fertilizer application have direct benefits in terms of N_2O emission reductions (see cropland nutrient management NCS), which are not captured in this analysis of MAC for avoided seagrass loss, suggesting that our MAC estimates may be conservative.

We were unable to locate any study that estimates the MAC of avoided U.S. coastal wetland loss. Such MAC curves could be constructed for each remaining seagrass area based on data about current and predicted N concentrations in each of these areas and on least-cost approaches for reducing N loading in each area to levels that sea grass can tolerate. Such an analysis was beyond the scope of our study. Instead, we estimate the MAC using data on the cost of reducing nitrogen pollution (which allowed recovery of seagrass in Tampa Bay, Florida). We use data on the cost of reducing nitrogen pollution from both Tampa Bay and from the Mississippi River watershed.

Tampa Bay had lost approximately half its 1950 seagrass extent by the 1970s, primarily due to extreme eutrophication (*323*). Actions implemented since 1980 in the Tampa Bay watershed to reduce nutrient loading into the then heavily eutrophied Bay included waste water treatment plant upgrades, stormwater treatment (to reduce N) and phosphate industry BMPs (to reduce P) (*323*) as well as, most recently, fossil fuel power plant upgrades for N control (*324*). These actions have reduced annual N loading into the Bay by an estimated 70% (6,900 Mg yr^{-1}) from its peak, from around 10,000 Mg N yr^{-1} in the 1970s (*325*) to 3,100 Mg N yr^{-1} in 2011 (*326*). Bricker (*327*) reports that a 60% N loading reduction from 1970 levels — achieved around 2005 (*325*) — carried an estimated average annual cost of USD 28.7M, while Cooper (*328*) reports that the 952 Mg N yr^{-1} reduction achieved between 1992 and 2011 had a total cost of >USD 760M, or USD 38M yr^{-1}. We conservatively assume that these annual average costs are calculated from current dollar values. To adjust these values to 2015USD, we assume equal distribution of current dollar-value costs across their respective time periods [Bricker (*327*): 1980-2005; Cooper (*328*): 1992-2011] and adjust them using the U.S. Bureau of Labor Statistics Consumer Price Index (*75*). This results in a time series length-weighted mean of these average costs of 2015USD 51.25M yr^{-1}, and an estimated average annual cost-effectiveness of N load reductions of USD 7,420 per Mg of N.

Largely attributed to N (and P) load reductions, sea grass extent in Tampa Bay has increased from a low of around 8,200 ha in the 1970s (*323*) to 16,860 ha in 2016, slightly surpassing extent in the 1950s (*329*).

Given estimated GHG emissions from seagrass loss of 1.67 Mg C ha^{-1} yr^{-1} (*62*) and assuming that the N loading reductions in Tampa Bay achieved between 1980 and the present would have prevented the historic reduction in seagrass extent in the Bay had they been implemented in the 1950s and 1960s, the avoided loss of the 8,660 ha of sea grass recovered between the 1970s and 2016 would have avoided annual emissions of 53,040 Mg CO_2e yr^{-1}.

Given the estimated annual cost of the N loading reductions, this results in an estimated cost of avoided GHG emissions from sea grass loss in Tampa Bay of USD 966 Mg CO_2e^{-1} yr^{-1}.

The representativeness of this estimate for the MAC of avoided sea grass loss nationally may be limited for two reasons: first, the Tampa Bay N control effort appears not to have employed any market-based policy instruments to produce efficiency gains and result in lower control costs, such as cap-and-trade mechanisms for N loading coupled with point-nonpoint sources trading. Second, in many estuaries, agricultural BMPs may account for an important portion of total potential N load reductions. However, agricultural BMPs were not part of the Tampa Bay N loading control strategy. Finally, the relation between N loadings and seagrass loss is a function of the residence time and volume (depth) of coastal water in the area in question; the conditions of which in Tampa Bay may not represent the national average for remaining seagrass habitat.

Rabotyagov *et al.* (*330*) estimate the MAC of reducing the extent of the Gulf of Mexico hypoxic zone through implementation of cost-effectively targeted nutrient load reductions from agricultural lands in the Mississippi-Atchafalaya River Basin. Based on the eight hypoxia reduction extents and associated costs shown in their table S2, and assuming that hypoxia avoidance would have had the same costs as ex-post reduction of hypoxia and that hypoxia prevention would have avoided seagrasses loss, the MAC of avoided sea grass loss through prevention of hypoxic conditions in the Gulf of Mexico would have the MAC shown in fig. S23. Note that the actual potential for GHG emission reductions from avoided sea grass loss in the Gulf of Mexico is much lower than indicated in the figure given that much of the reduction in hypoxia extent modeled in Rabotyagov *et al.* (*331*) occurs in waters too deep for sea grass. These cost estimates per Mg CO_2e are up to one order of magnitude lower than those calculated for Tampa Bay. A 3rd order polynomial fits the data well and intersects the y axis at USD 200 Mg CO_2e^{-1}, indicating that no avoided GHG emissions from avoided sea grass loss would be obtainable for USD 100 Mg CO_2e^{-1} or less.

Seagrass Restoration

To calculate restoration potential, we assumed that, if steps were taken to improve water quality in areas that previously supported seagrasses, seagrasses could be restored to their historic extent. Because seagrass distribution is limited by light attenuation, sea level rise may submerge seagrasses to depths at which they do not have enough light survive, thereby limiting the ability to restore seagrasses to their full historic extent. However, restoration activities that clean waters (i.e. reduce turbidity) allow seagrass growth at greater depths (*332*). Notably, in clear waters, seagrass can occur up to depths of 90 m (*333*). Therefore, our analysis assumes that any seagrass area lost due to the modest increase in sea level rise expected by 2025 is offset by increased growth from reductions in turbidity and landward migration of seagrass.

We used the mean of two approaches to determine the area available for seagrass restoration in the U.S. The first approach uses the Waycott *et al.* (*61*) finding that seagrasses have declined by at least 29% since the beginning of the 20th century (a conservative estimate generated by comparing current extent with the area of mapped seagrasses at the turn of the 20th century, surely an underestimate because of incomplete mapping of historic extent). The second method uses the Waycott *et al.* (*61*) average loss rate of 1.5% yr^{-1} and our current estimate of seagrass extent. We started with the current estimated area of 1.44 Mha (*17*) and back-calculated to find

the area that would have existed in 1940 based on a 1.5% annual loss rate. These two approaches suggest a range of 0.59 to 3.00 Mha of former seagrass habitat that could be restored, with a mean of 1.79 Mha. We use data from six seagrass restoration sites in the U.S. older than two years (*63*, *334*) to estimate an average sequestration rate in restored seagrass sediments of 0.89 Mg C ha^{-1} yr^{-1}. We apply this rate to the mean area calculated above (1.79 Mha), to find a mitigation potential from seagrass restoration of 5.9 Tg CO_2e yr^{-1}.

Uncertainty for seagrass restoration

To calculate extent uncertainty, we used the results from the two approaches to estimate both the mean and the upper and lower bounds (*61*), which results in a range of ±67%. To calculate flux uncertainty, we used the measurements from the six restoration sites that were used to estimate sequestration rate to calculate a 95% CI of ±58% (*64*, *337*). Combined, this yields an overall expected range of ±89%, or 0.65 to 11.2 Tg CO_2e yr^{-1}

Marginal abatement costs for seagrass restoration

The MAC curve for this pathway was generated using the data on U.S. seagrass restoration projects in the Bayraktarov *et al.* (*335*) database, with costs updated to their 2015 values (*75*). Emissions reductions were calculated using the total project area from the database and our estimated annual sequestration rate ha^{-1}, multiplied by the mean probability of restoration success as reported for those projects in Bayraktarov *et al.* We calculate restoration costs per Mg CO_2e as (restoration costs ha^{-1} * #ha)/(#ha * emissions reductions ha^{-1}). The cost data in the Bayraktarov *et al.* database include technical restoration costs (capital and operating costs) but not monitoring, opportunity or transaction costs. Even though costs are reported in the database as ha^{-1} yr^{-1}, they appear to refer to costs during project implementation, causing a temporal mismatch between reported annual costs and reported annual carbon sequestration estimates, which are given as Tg ha^{-1} yr^{-1}. To remove this mismatch, we annualized restoration costs using the formula

$$AC=PVC*(r*(1+r)^{n}) / ((1+r)^{(n+1)}-1)$$

(*336*), where r is the discount rate, n is the duration of the policy, PVC is the present value of the costs, and AC is the annualized value of the costs. As the discount rate, we used the U.S. Lending Interest Rate, which averaged 3.26% in 2015 (*337*). For the duration of the policy we used 100 years, based on the time until saturation, which for seagrass is >100 years (table S1). We find that all seagrass restoration projects were substantially more expensive than USD 100 Mg CO_2e^{-1}.

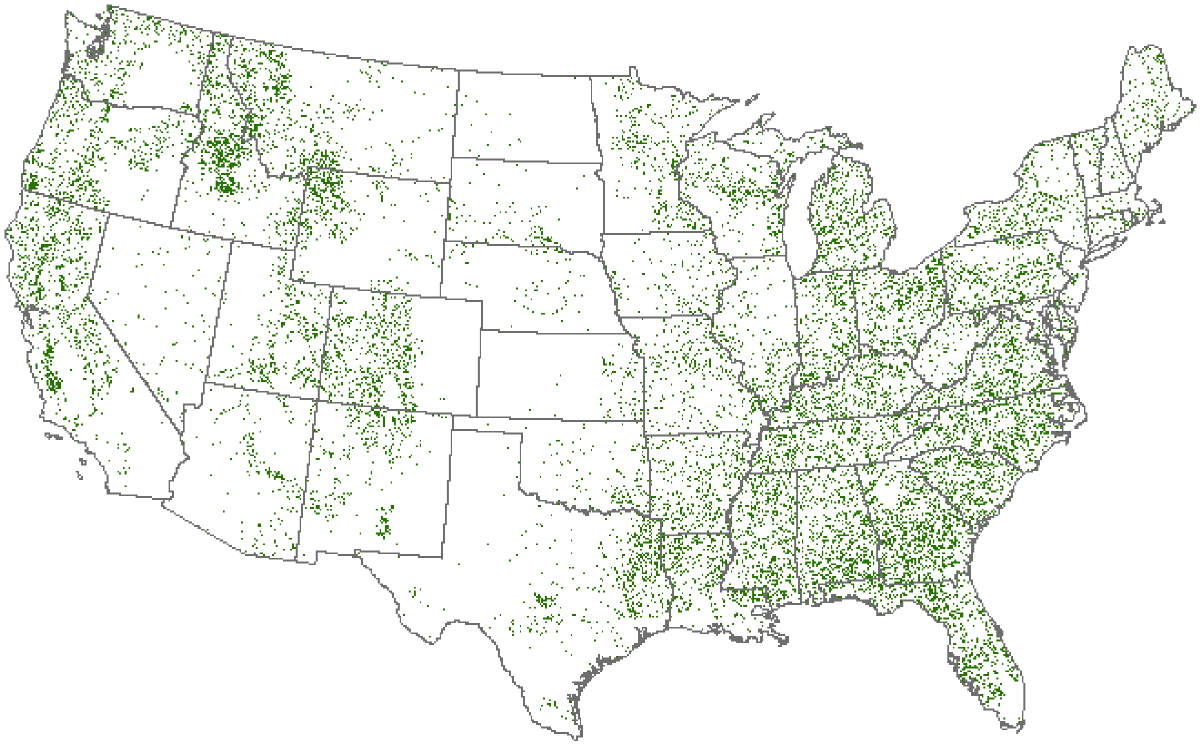

Fig. S1. Mapped reforestation opportunity areas in the lower 48 states. Dark green areas represent reforestation opportunities in areas historically forested that are currently unforested. We do not depict crop or pasture land as we removed the majority of these lands (see text).

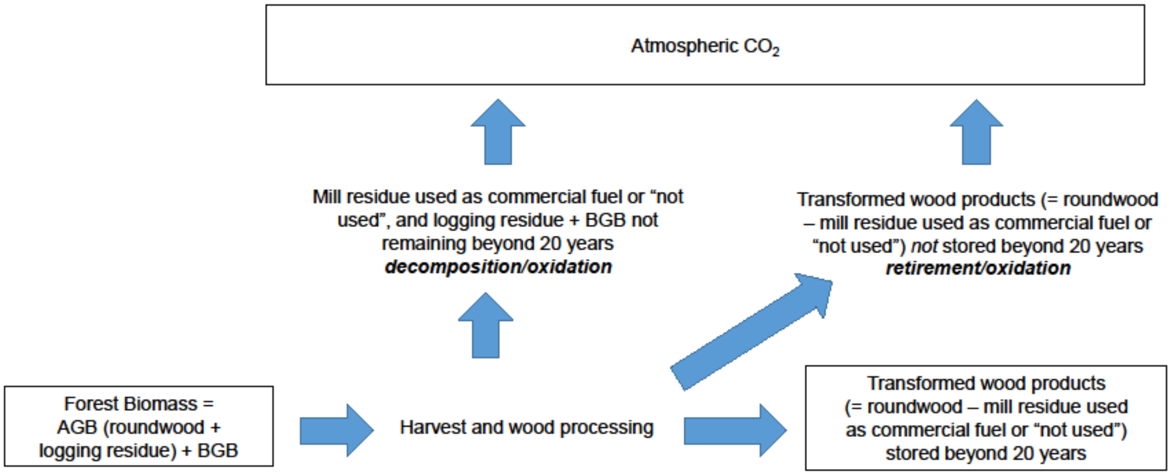

Fig. S2. Conceptual framework for improved forest management carbon accounting. All carbon that starts as forest biomass is, twenty years after harvest, either emitted as CO_2 via decomposition or combustion or is retained in woody biomass or long-lived wood products.

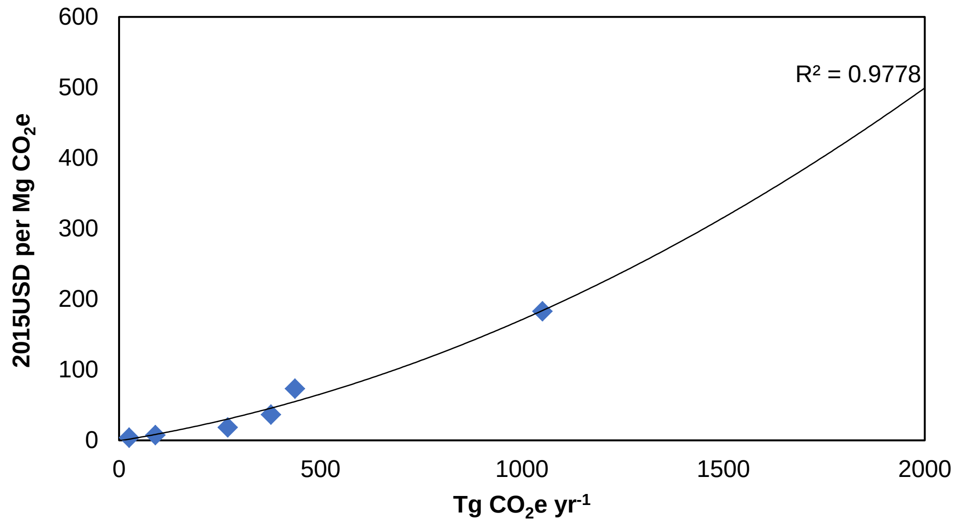

Fig. S3. MAC for carbon sequestration through forest management and aging, after Golub *et al*. (*99*).

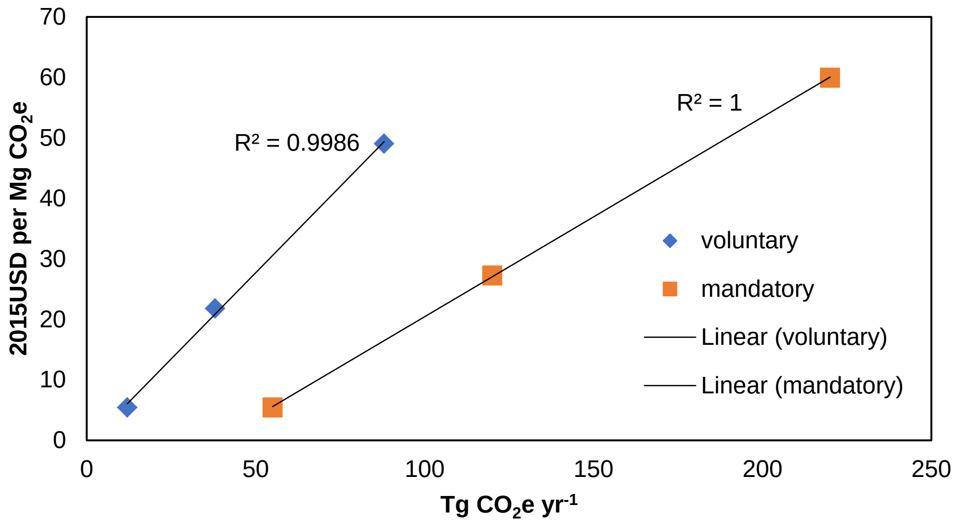

Fig. S4. MAC for natural forest management after Latta *et al*. (*98*) and best-fit functions.

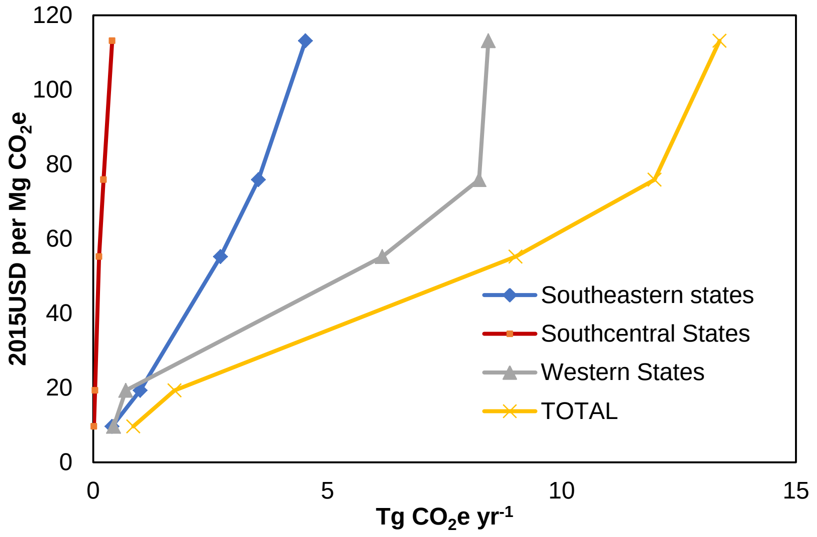

Fig. S5. MAC curves for improved plantations. From Sohngen and Brown (*130*), with sequestration converted from discounted present value to annual equivalents.

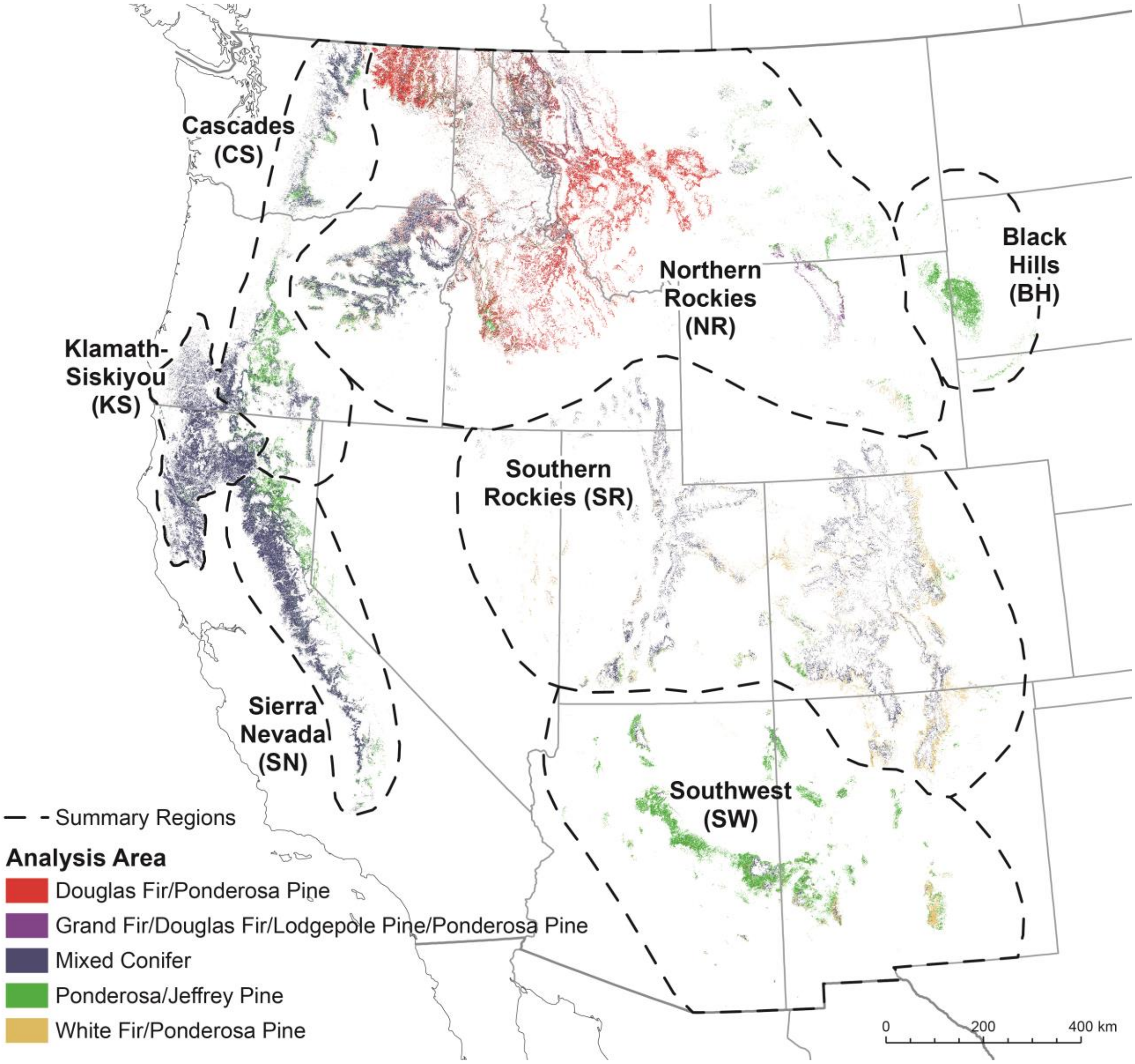

Fig. S6. Fire management analysis area.

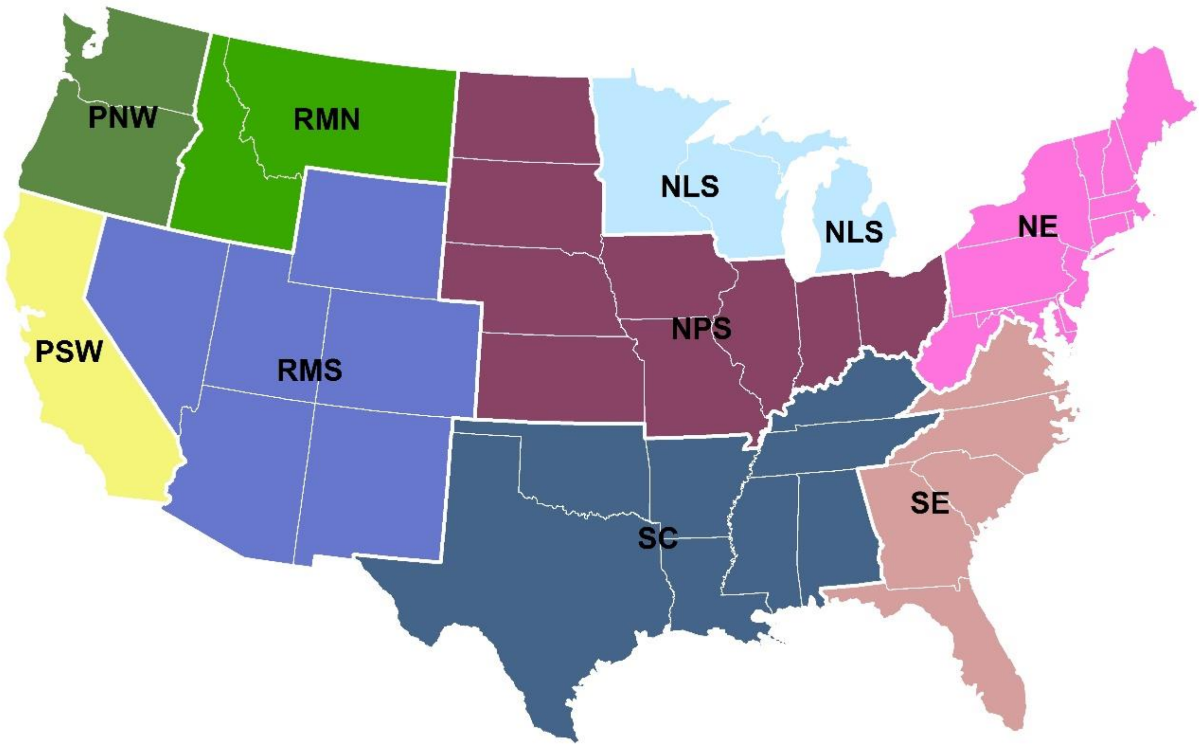

Fig. S7. Regions used for reporting avoided forest conversion results.

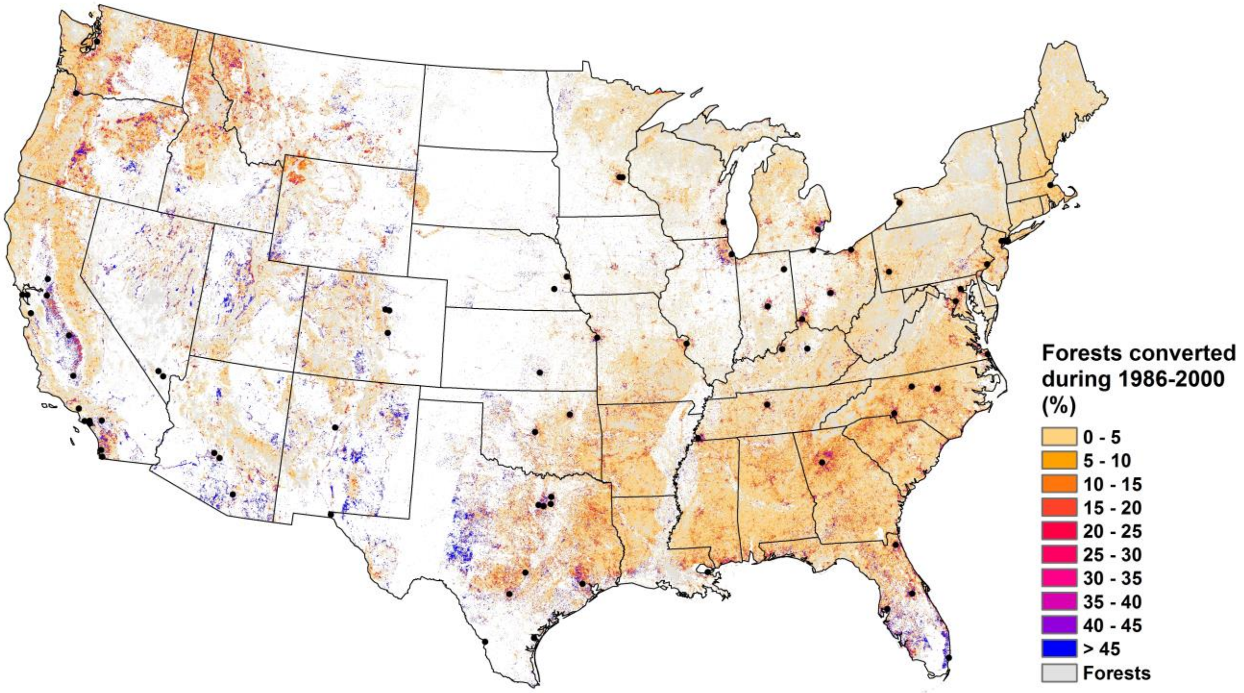

Fig. S8. Forest conversion from 1986 to 2000. Percentage of forest pixels converted, mapped at a 990 m x 990 m resolution. All cities with a population greater than 250,000 are displayed as black dots.

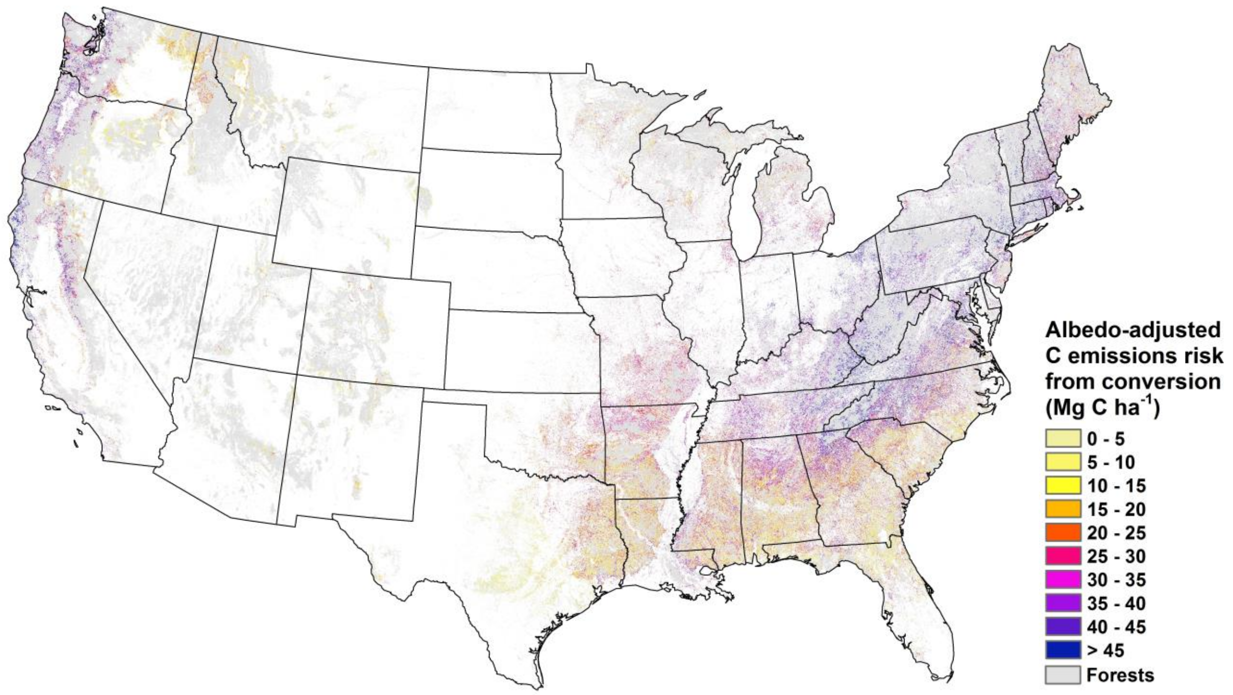

Fig. S9. Potential carbon emissions from areas at high risk of forest conversion. Potential carbon emissions that would occur if areas forested in 2010 were to be converted to non-forest, shown only for areas at highest risk of conversion, and adjusted for an albedo-related cooling offset in coniferous forests. High conversion risk was defined as 990 m x 990 m areas which experienced the top 25% rates of forest conversion during the historic period of 1986 to 2000 (>1.06% of forest converted per year).

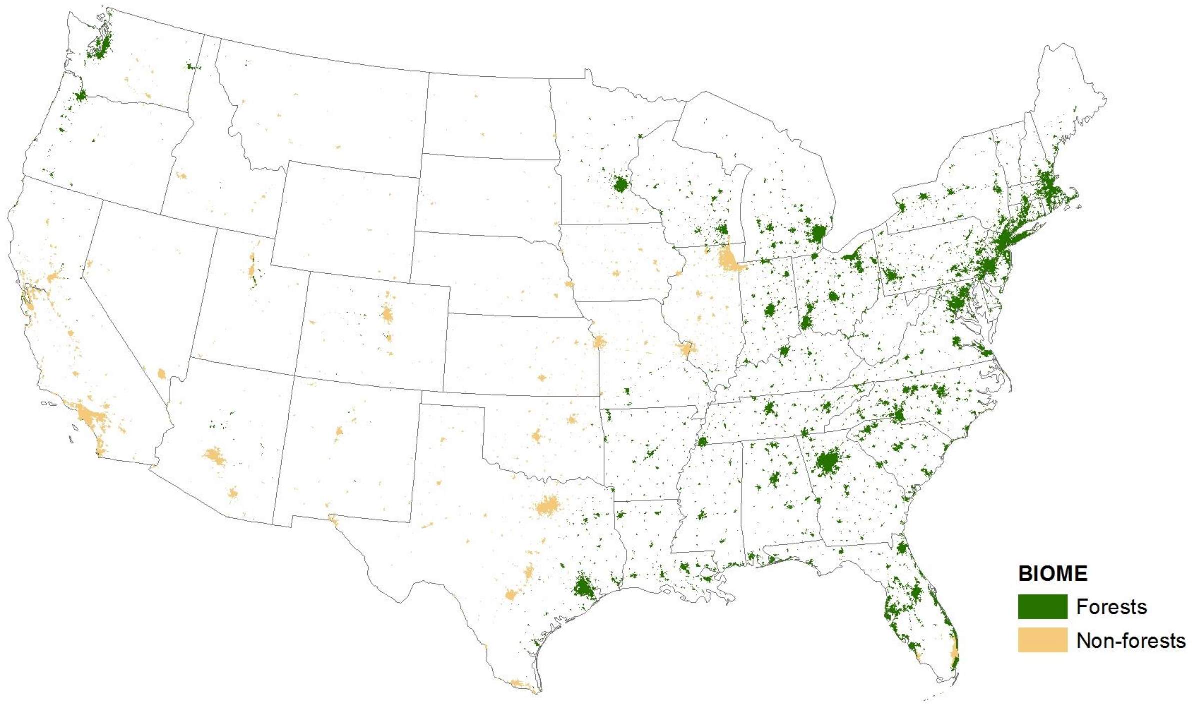

Fig. S10. Cities included in the urban reforestation analysis. In cities in forest biomes, we estimated the mitigation potential for both street trees and patches of trees. In cities in non-forest biomes, we only estimated the mitigation potential from street trees.

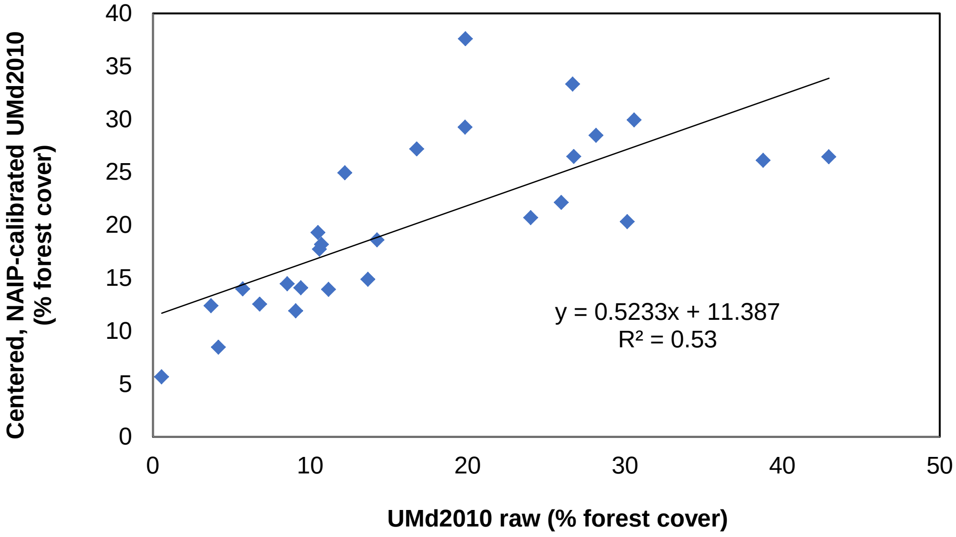

Fig. S11. Calibration of remote sensing data for forest cover estimation in urban areas. Linear regression to produce the correction equation used to account for under-representation of street trees in the 30-meter forest dataset (*168*). Relationship between UMd 2010 %FC and NAIP-calibrated, centered UMd 2010 %FC in 27 U.S. cities (see text).

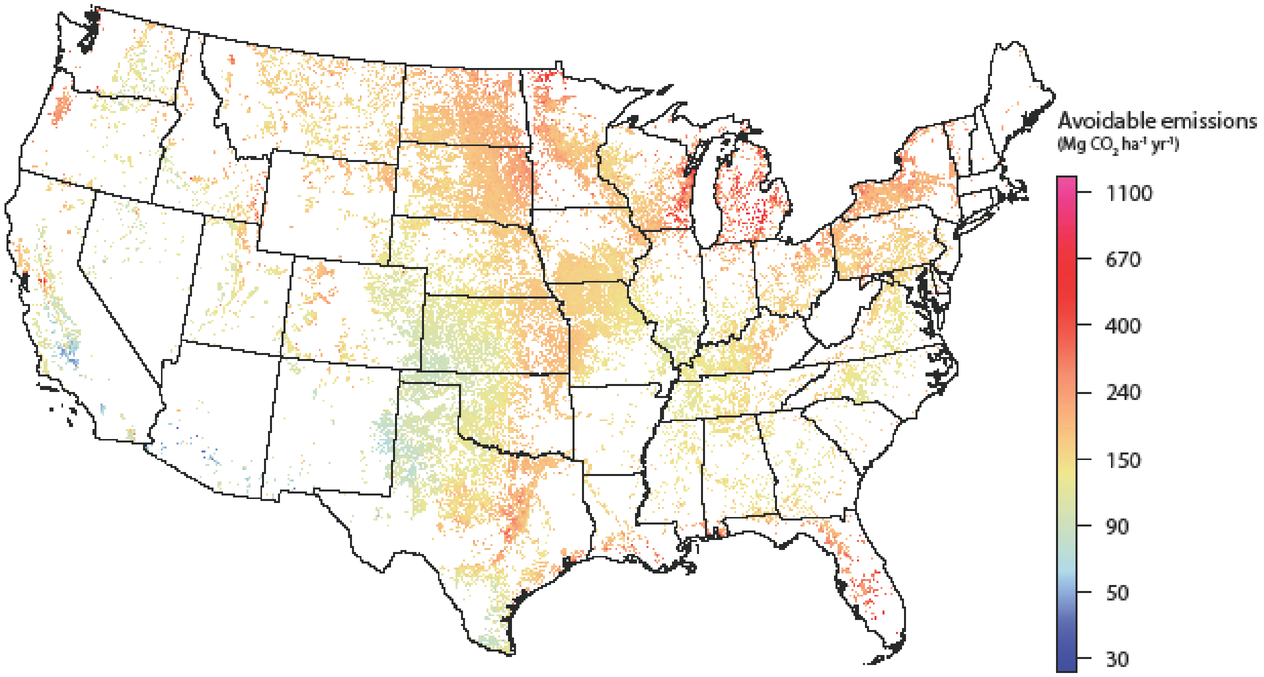

Fig. S12. Avoided grassland conversion map. Pixels show the per hectare emissions from grasslands and shrublands converted in the conterminous U.S. in 2008 to 2012 (*25*).

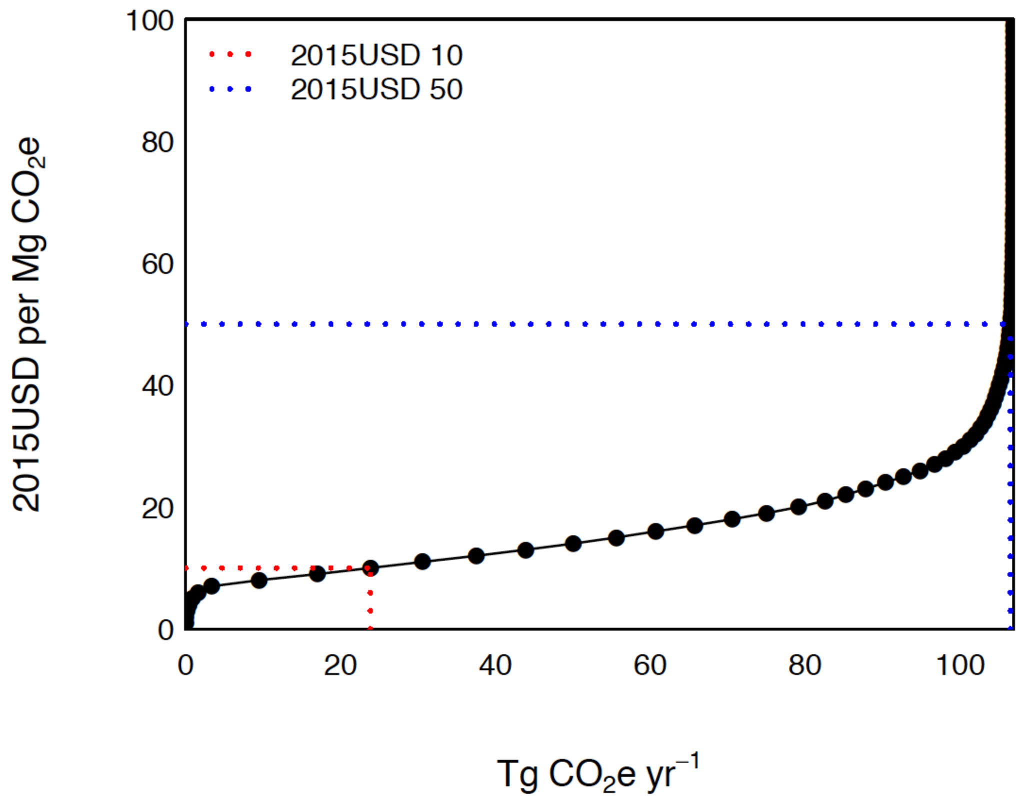

Fig. S13. MAC curve for avoided grassland conversion. Costs are based on county-level average payments for Conservation Reserve Program easements. Mitigation is based on avoided loss of soil carbon and root biomass (see text).

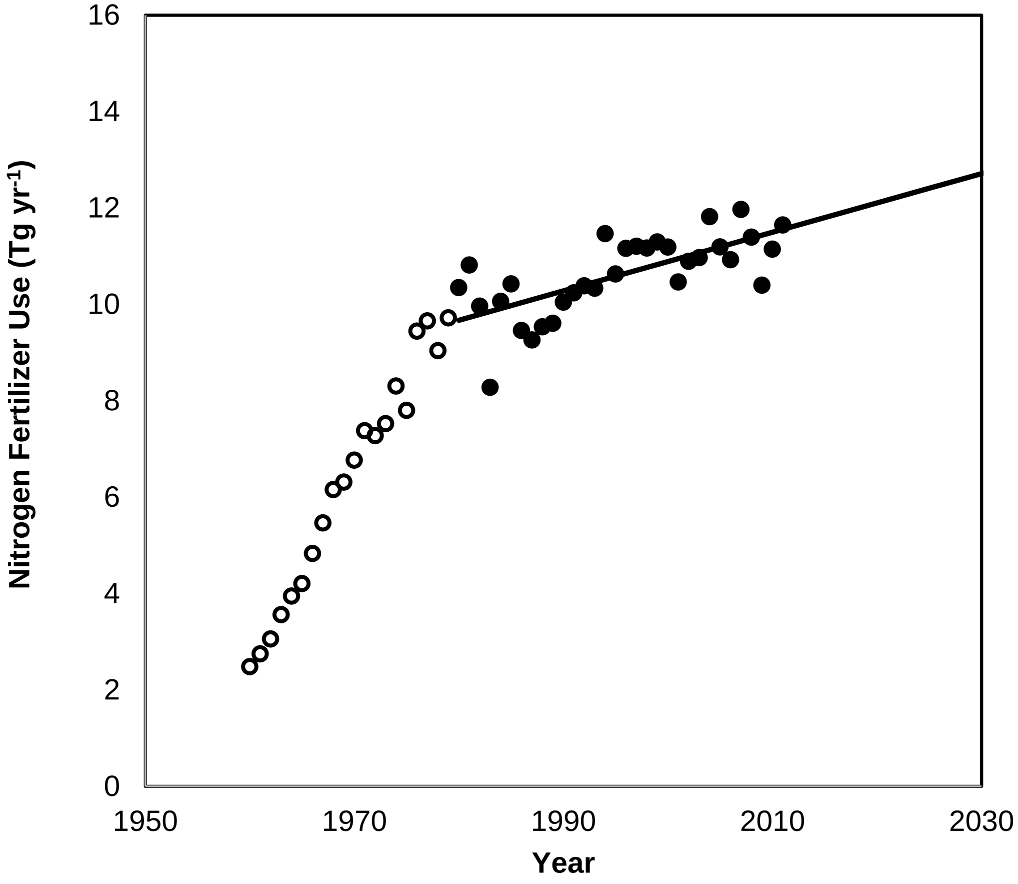

Fig. S14. Nitrogen fertilizer use in the United States. Data from 1960 through 2010 with linear model based on data from 1980 to 2011 extrapolated to 2030 (*334*).

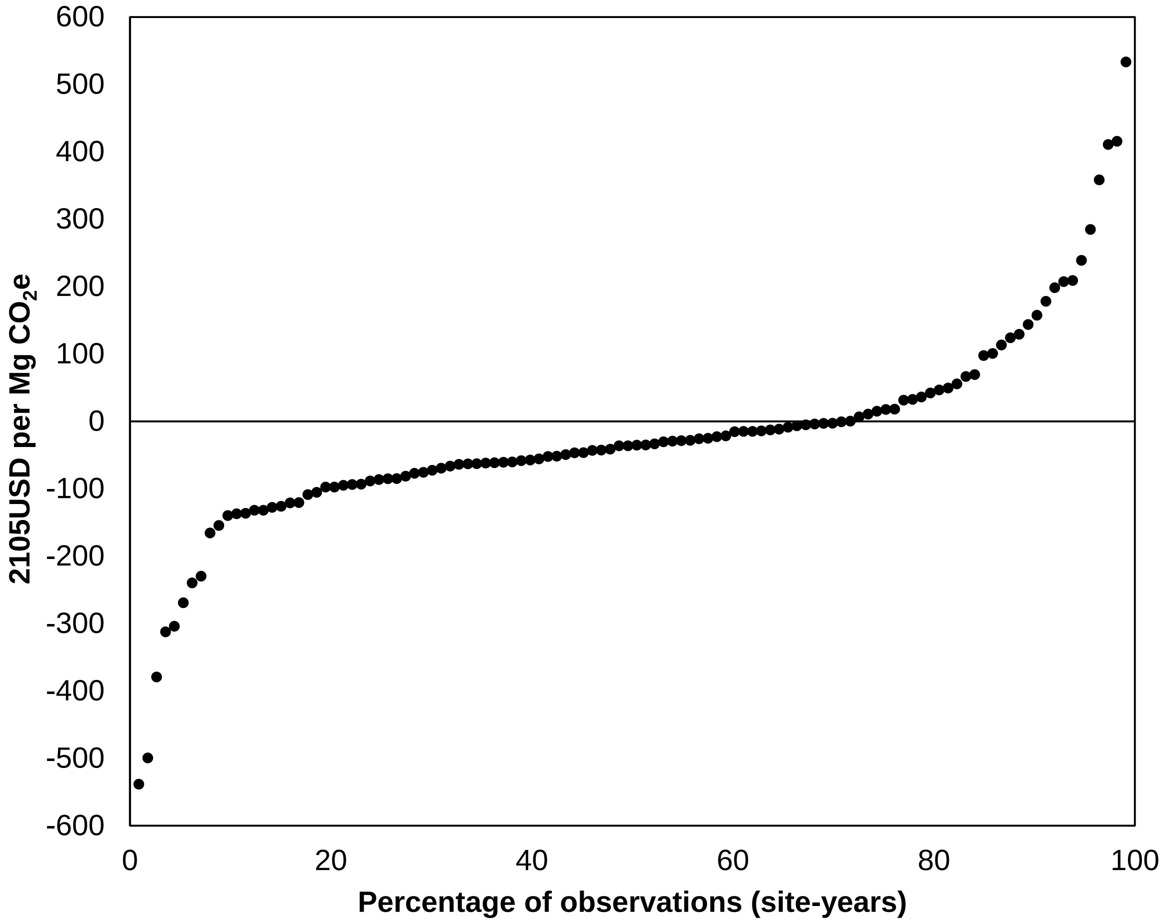

Fig. S15. Marginal abatement cost curve for reducing N fertilizer rate. Rate reduction uses a precision tool based on field trials from maize fields in the USA. Includes upstream, direct, and indirect N_2O emissions [based on data in (*259*)].

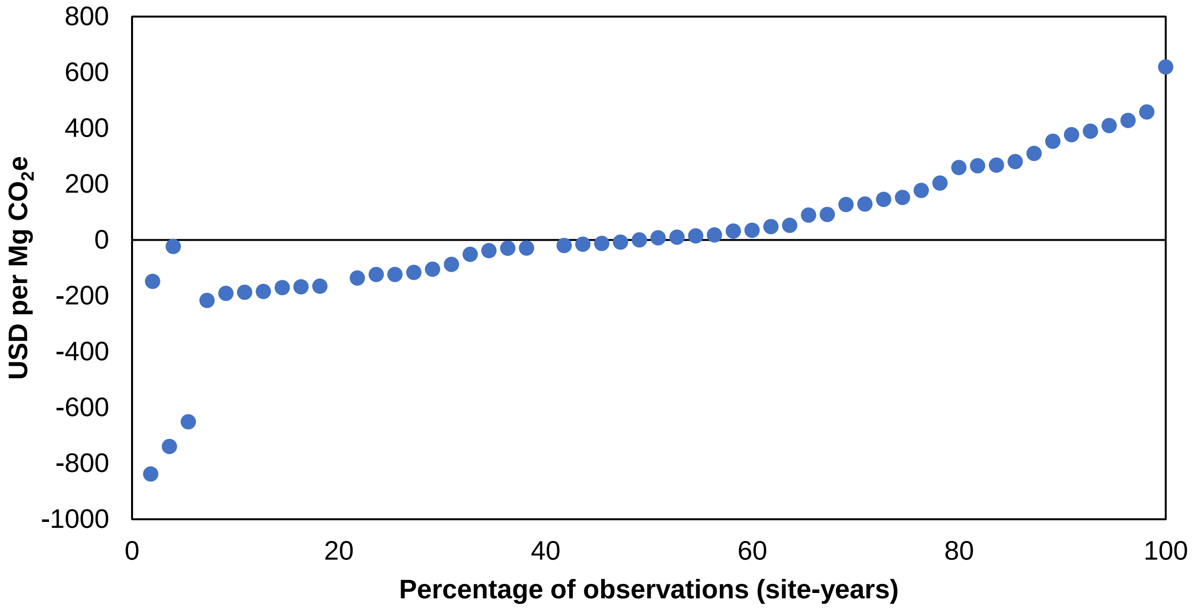

Fig. S16. Marginal abatement cost curve for applying variable rate technology fertilizer application. Based on field trials from maize fields in Missouri. Includes upstream, direct, and indirect N_2O emissions (see text for details) [data source for N reduction and profit change: (*276*)].

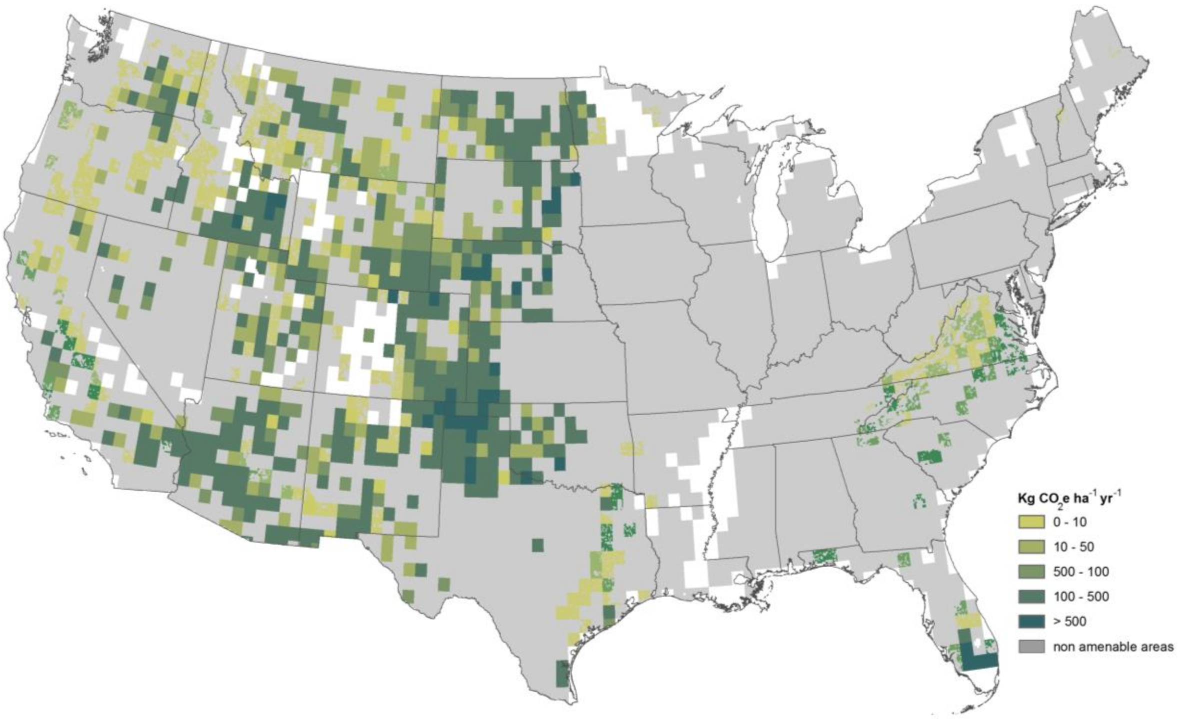

Fig. S17. Grazing optimization map. Colors indicate the annual per hectare sequestration rate from grazing optimization. White areas do not contain “managed grasslands,” where managed grasslands are defined as having >1 head of cattle per square km. Gray areas are “non-amenable” because grazing optimization would not result in additional carbon sequestration.

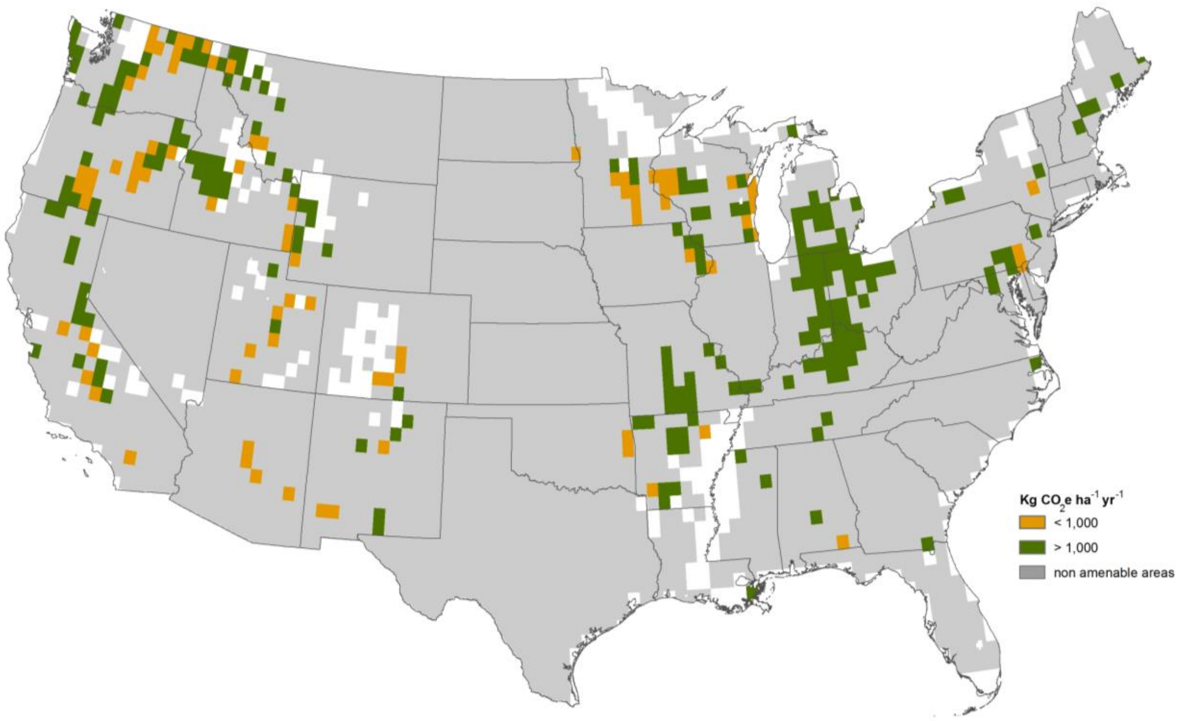

Fig. S18. Legumes in pastures map. Colors indicate the annual per hectare mitigation rate from interseeding legumes in planted pastures. Note this mitigation rate accounts for both increased soil carbon accumulation and increased emissions of N_2O associated with legumes. White areas do not contain “managed grasslands”, where managed grasslands are defined as having >1 head of cattle per square km. Gray areas are “non-amenable” because interseeding legumes would not result in additional net mitigation.

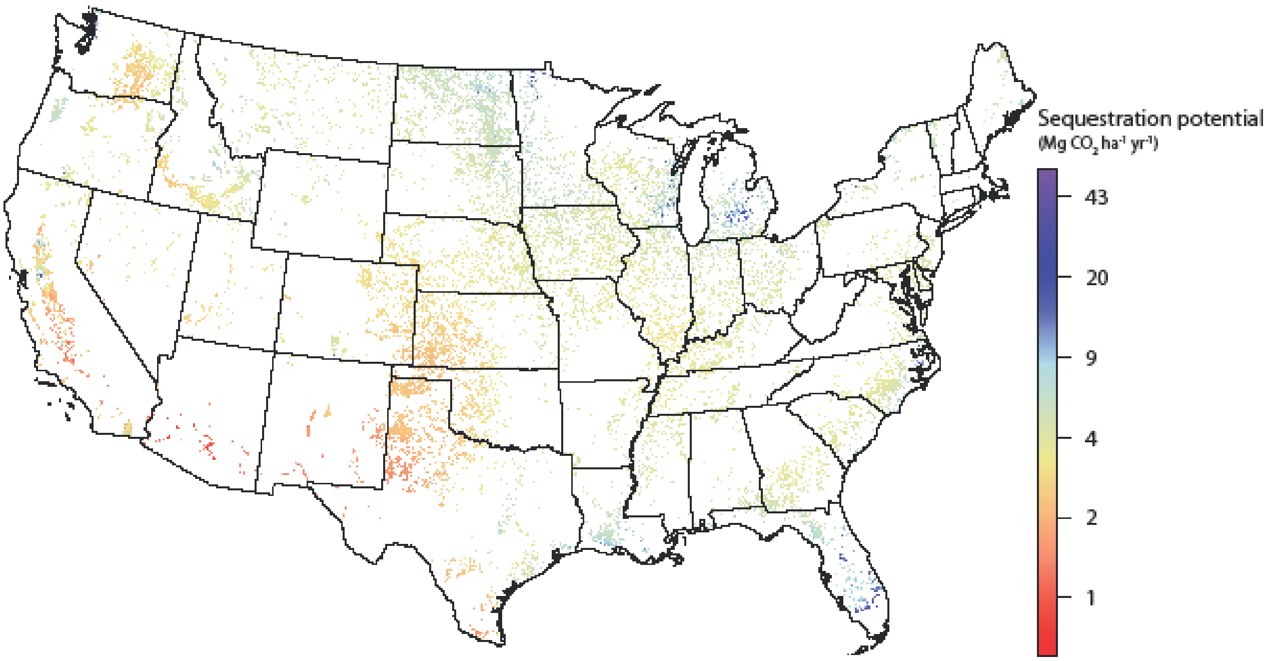

Fig. S19. Grassland restoration map. Pixels show annual per hectare sequestration rates for likely areas of grassland restoration, defined as areas in the conterminous U.S. where cropland was abandoned to grassland 2008-2012 (*25*).

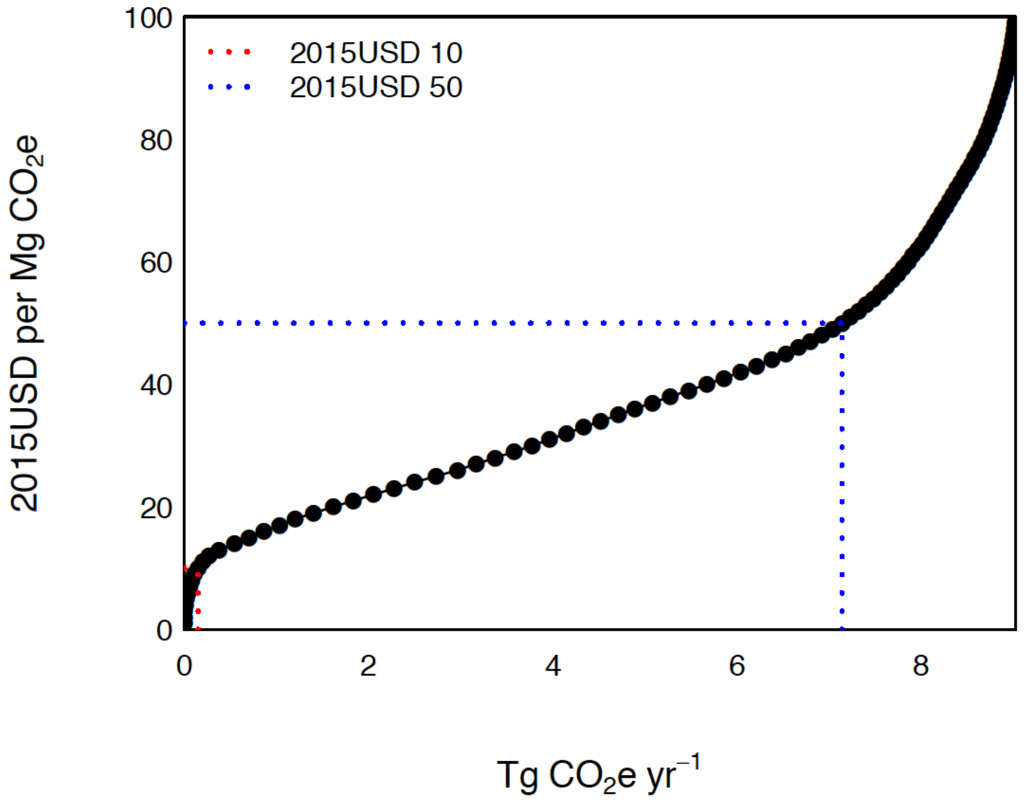

Fig. S20. MAC curve for grassland restoration. Costs are based on county-level average annual payments for Conservation Reserve Program easements. Mitigation is based on annual carbon sequestration in soils and root biomass (see text).

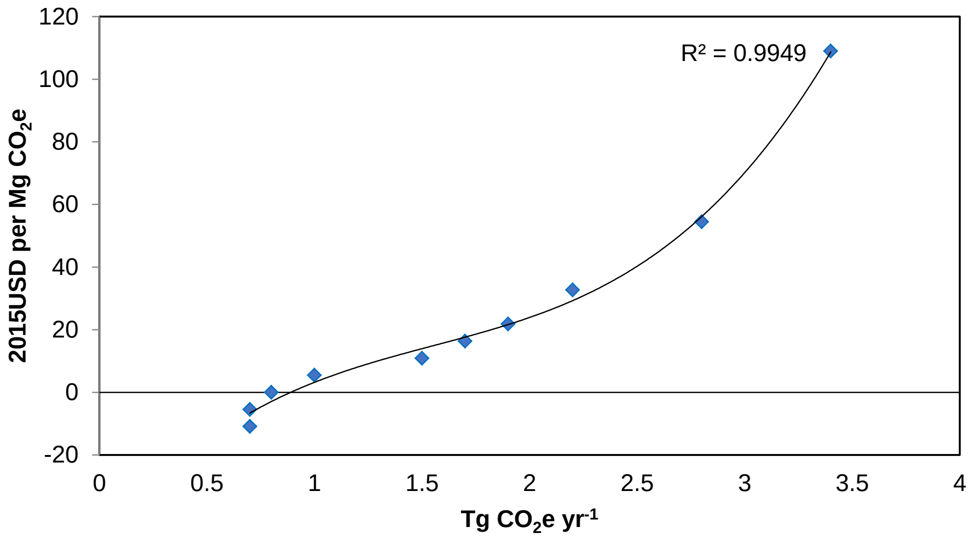

Fig. S21. Break-even prices for GHG abatement from rice production.

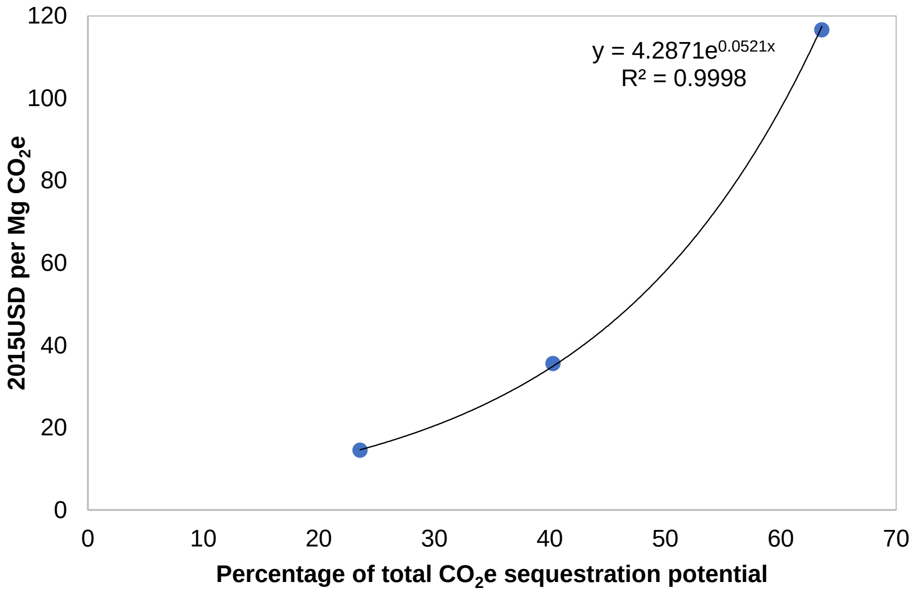

Fig. S22. MAC curve for salt marsh restoration. We used restoration projects in the U.S. with a MAC of up to approximately USD 120 Mg CO_2e^{-1} in Bayraktarov *et al.* (*331*) database and best-fit (exp.) function to estimate the MAC curve. The X-axis indicates the percentage accounted for by these relatively affordable projects out of the total sequestration achievable by all U.S. saltmarsh restoration projects in the database. Bayraktarov *et al.* (*331*) database.

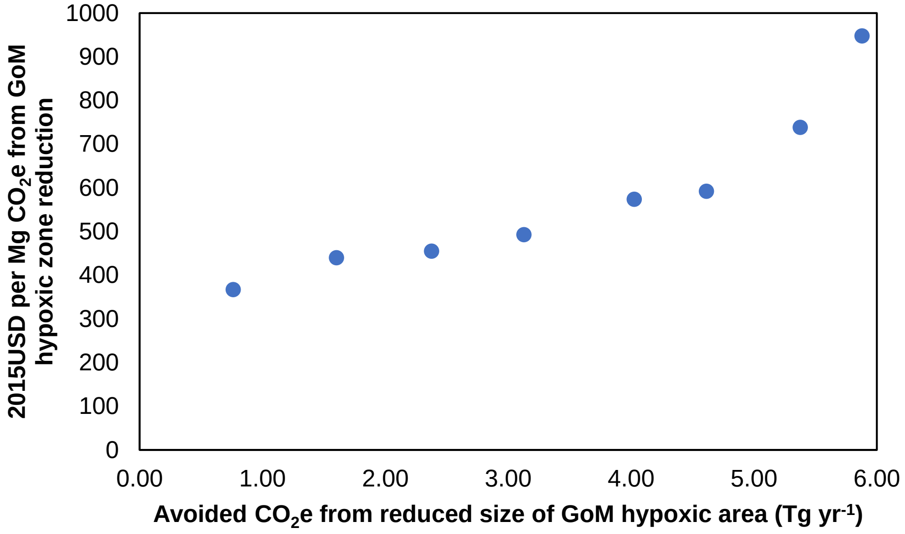

Fig. S23. MAC of avoided GHG emissions from seagrass. Avoided emissions due to avoided hypoxic conditions

Table S1. Mitigation potential of NCS in 2025. The length column refers to the number of years following NCS implementation until saturation, after which additional carbon storage slows or stops. Biochar, cropland nutrient management, and improved manure management are not based on areal estimates, but rather the amount of agricultural residue, nitrogen fertilizer, and hog and dairy head, respectively. See text for additional details.

**NCS Name**	**Area (Mha)**	**Flux (Mg Ce ha^{-1})**	**Length (yr)**	**Max (Tg CO_2e yr^{-1})**	**MAC USD 100**	**50**	**10**
					**(Tg CO_2e yr^{-1})**		
Reforestation	63 (34-92)	1.33 (0.17-5.01) yr^{-1}	>90	307 (90-777)	252	252	11
Natural Forest Mgmt.	123	0.59 yr^{-1}	25	267 (232-302)	267	229	64
Fire Mgmt.	17	0.29 (-0.09-0.67) yr^{-1}	>100	18 (-5-42)	13	10	0
Avoided Forest Conv.	0.38 (0.33-0.43) yr^{-1}	27 (17-38)	>100	38 (22-53)	38	38	37
Urban Reforestation	3.3 (2.6-4.0)	1.90 (1.62-2.47) yr^{-1}	>40	23 (19-30)	0	0	0
Improved Plantation	31	0.11 yr^{-1}	50	12 (11-14)	12	8	1
Avoided Grassland Conv.	0.69 (0.60-0.78) yr^{-1}	42 (22-70)	>100	107 (55-188)	107	107	24
Cover Crops	88	0.32 (0.16-0.48) yr^{-1}	>50	103 (53-154)	103	100	100
Biochar			>100	95 (64-135)	95	9	0
Alley Cropping	15 (8-80)	1.45 (0.74-2.16) yr^{-1}	>50	82 (35-166)	82	66	4
Cropland Nutrient Mgmt.			>100	52 (17-121)	50	32	28
Improved Manure Mgmt.			>100	24 (18-30)	22	20	12
Windbreaks	0.9 (0.4-1.7)	3.56 (1.88-5.23) yr^{-1}	>50	11 (3-30)	11	11	5
Grazing Optimization	53	0.05 yr^{-1}	>100	11 (-13-35)	9	8	6
Grassland Restoration	2.1	1.19 (0.49-2.43) yr^{-1}	>50	9 (3-21)	9	7	0
Legumes in Pastures	5.5	0.35 yr^{-1}	>100	7 (-3-17)	6	5	3
Improved Rice Mgmt.	1.1 (1.0-1.2)	0.92 (0.81-1.03) yr^{-1}	>100	3.7 (3.2-4.2)	3	3	2
Tidal Wetland Restoration	0.48 (0.23-0.73)	6.7 (0.8-12.6) yr^{-1}	>100	12 (0-24)	7	5	2
Peatland Restoration	3.0	0.80 (0.43-1.01) yr^{-1}	>100	9 (5-11)	9	7	0
Avoided Seagrass Loss	0.02 (0.00-0.05) yr^{-1}	89 (36-142)	67	7 (2-11)	0	0	0
Seagrass Restoration	1.8 (0.6-3.0)	0.89 (0.37-1.41) yr^{-1}	>100	6 (1-11)	0	0	0
Total				1,204 (855-1,644)	1,097	918	299

Table S2. Co-benefits of NCS. We summarize publications providing evidence that a given type of ecosystem service is enhanced due to implementation of a pathway. Cells in white indicate cases where we did not identify clear evidence of enhanced ecosystem services. See Materials and Methods section for definition of each of the four service types (biodiversity, water, soil, air).

**Pathway**	**Air** (filtration)	**Biodiversity** (alpha, beta, gamma)	**Soil** (enrichment)	**Water** (filtration, flood control)
Forests				
Reforestation	Ozone abatement benefits of reforestation (*101*). Multiple modeling studies describe health benefits of air filtration by forests (*183*, *339*).	Tree plantings can create wildlife corridors and buffer areas that enhance biological conservation (*340*).	Measured increase in soil fauna in reforested sites. During drought conditions earthworms only survived in reforested areas (*341*).	Improved availability of water for crop irrigation, drought mitigation; avoided sedimentation and water regulation for hydroelectric dams (*342*).
Natural Forest Management		"Species richness of invertebrates, amphibians, and mammals decreases as logging intensity increases" (*343*).	Timber harvesting that removes large amounts of woody debris reduces soil biological and physical properties thereby reducing health and productivity (*344*).	Harvesting that removes large proportions of biomass increases water flows and flooding thereby altering freshwater ecosystem integrity (*345*).
Fire Management	"Possibility of small increases in mortality due to abrupt and dramatic increases in particulate matter concentrations from wildfire smoke" (*346*).	Fire management that mimics natural historic fire regimes can improve forest biodiversity (*347*).	Forests that survive fires (i.e. reduced catastrophic wild fires) contain more organic matter, improved soil properties, and lower recovery times enhance water infiltration and retention (*132*).	Increased runoff associated with severe forest fires due to eliminating the water holding capacity of the near surface organic layer and surface vegetation (*132*).
Avoided Forest Conversion	Ozone abatement benefits of reforestation (*101*). Multiple modeling studies describe health benefits of air filtration by forests (*183*, *339*).	"Results indicate the irreplaceable value of continuous primary forests for conserving biodiversity" (*348*).	Water retention and flow regulation (*346*). Maintains soil biological and physical properties ensuring health and productivity of forests (*344*).	Improved availability of water for crop irrigation, drought mitigation; avoided sedimentation and water regulation for hydroelectric dams (*342*).
Urban Reforestation	Pollutant removal including O3, SO2, NO2, CO, and particulate matter (*349*, *350*).	Researchers found that "urban green space with natural structures can maintain high ecological diversity" of bird species (*351*).		Urban forests can attenuate flooding for extreme weather events by storing water and limiting runoff (*352*).

**Pathway**	**Air** (filtration)	**Biodiversity** (alpha, beta, gamma)	**Soil** (enrichment)	**Water** (filtration, flood control)
Improved Plantations		Forest plantations that consider community type such as polycultures over monocultures, native over exotics, disturbance pattern replication, longer rotations, and early thinning can enhance biodiversity (*353*).		
Agriculture and Grasslands				
Avoided Grassland Conversion	Cropland causes air quality issues due to ammonia and particulates that have significant health impacts (*260*, *261*).	Important habitat for nesting and foraging birds (*354*).	Perennial grasses have little soil and nutrient loss compared to cropland (*355*, *356*).	Permanent grasslands provide "biological flood control" and maintain ecosystem water balance assuring adequate water resources (*357*).
Cover Crops			Reduces soil erosion and redistribution maintaining soil depth and water retention (*358*).	Reduces agricultural water demands with appropriate cover crops (*359*).
Biochar			The addition of biochar enhances soil quality and fertility in temperate regions (*360*).	
Alley Cropping	Tree planting helps capture airborne particles and pollutant gasses (*358*).	Agroforestry provides habitat for species and supports connectivity (*361*).	Decreased soil erosion (*362*).	Sediment retention and water recharge (*361*).
Cropland Nutrient Management	Nitrogen fertilization causes air quality issues due to ammonia and particulates, which are reduced at lower fertilization rates (*363*).	Increased indicators of stream health from macroinvertebrates (*364*) and leaf litter breakdown (*365*)		Reduced nitrate leaching (*366*) has benefits associated with improved drinking water quality, increased opportunities for recreation, and health benefits (*367*).
Improved Manure Management	Reduced N_2O and CH_4 emissions from manure management (*368*).	Hypoxic conditions related to manure runoff from agriculture causes attributed to Gulf of Mexico "dead zone" (*369*).	Manure management increase soil nutrients (*368*).	Proper management and timing of the use of manure as a soil amendment limit the chances of increased Nitrogen and

**Pathway**	**Air** (filtration)	**Biodiversity** (alpha, beta, gamma)	**Soil** (enrichment)	**Water** (filtration, flood control)
				Phosphorus runoff (*370*).
Windbreaks	Tree planting helps capture airborne particles and pollutant gasses (*358*).	Agroforestry provides habitat for species and supports connectivity (*361*).	Decreased soil erosion (*362*).	Erosion control and water recharge (*361*).
Grazing Optimization		A gradient of intensive to extensively grazed pastures reduces overall disturbance to plant-insect interactions (*371*).	Over grazing can reduce the soils ability to trap contaminants and cause a release of these and other suspended sediments (*358*).	Nearly 70% of water use for cattle occurs during farm grazing, managed grazing practices can reduce water use on managed pastures (*372*).
Grassland Restoration	Cropland causes air quality issues due to ammonia and particulates that have significant health impacts (*258*, *259*).	Important habitat for nesting and foraging birds (*354*).	"Soil macroinvertebrates are important prey for breeding wading birds on lowland wet grassland" (*354*).	Permanent grasslands provide "biological flood control" and maintain ecosystem water balance assuring adequate water resources (*357*).
Legumes in Pastures		The presence of legumes in prairie leads to higher insect herbivore and insect predator diversity (*373*).	"Legumes provide other ecological services including improved soil structure, erosion protection and greater biological diversity" (*374*).	
Improved Rice Management				Alternating wet dry and midseason drainage of irrigated rice fields reduces water demands for agriculture (297). The use of gray water in agriculture can reduce gross water consumption (*375*).
Wetlands				
Tidal Wetlands Restoration		Maintains the provision of structure, nutrients and primary productivity and nurseries for commercial fish and shrimp (*321*, *376–378*).	Benefits of cross-system nutrient transfer to coral reefs, coastal protection, and water quality regulation (*379*).	Remove nutrients and sediments from estuarine waters (*380*).

Pathway	Air (filtration)	Biodiversity (alpha, beta, gamma)	Soil (enrichment)	Water (filtration, flood control)
Peatland Restoration	Exposure to pollutants from peat fires increases in the need for health services to treat lung and pulmonary disorders (*381*). Rewetting peatlands reduces fire risk (*382*).	Regeneration of peatlands re-establishes diverse communities (*383*).	Restoring degraded lands to high productivity depend on faunal species that help develop soil structure and fertility (*384*).	Removal of nutrients from surface and groundwaters and storm water remediation (*378*, *385*).
Avoided Seagrass Loss		Increases faunal species richness, abundance and diversity and serves as nurseries for commercially important fish and shrimps (*380*, *386*).	Wave attenuation protects shorelines from erosion (*380*).	Remove nutrients and sediments from marine waters (*380*).
Seagrass Restoration		"increases faunal species richness, abundance and diversity" and serves as nurseries for commercially important fish and shrimps (*386*).	Wave attenuation protects shorelines from erosion (*380*).	Remove nutrients and sediments from marine waters (*380*).

Table S3. Literature MAC estimates for reforestation of agricultural lands.

**MAC**<br><br>**2015USD Mg CO_2e^{-1}**	**Alig *et al.*** (*96*)	**Golub *et al.*** (*99*)	**Haim *et al.*** (*97*)	**Latta *et al.*** (*98*)**, voluntary**	**Latta *et al.*** (*98*)**, mandatory**	**Mean**
			**Tg $CO_2e\ yr^{-1}$**			
10	0	37	0	7	15	12
50	388	280	346	47	96	231
100	557	595	495	108	212	393

Table S4. Literature estimates of reforestation costs used to estimate MAC of reforesting natural ecosystems.

**Source**	**Reforestation location and type**	**Detail of cost**	**Cost ha^{-1}, 2015USD**
Kroeger *et al.* (*101*)	TX bottomland hardwoods	Average of low and high cost assumptions; 730 seedlings ha^{-1}	728
Stanturf *et al.* (*103*)	MS bottomland hardwoods	Seedlings planted at density needed to achieve 309 stems ha^{-1} target density	524
USDA (*387*)	U.S. National forests	Average cost in FY 2007 of forest vegetation establishment on National Forests [from pp. 8-28 and 8-30 in USDA (*387*)]	1525
Sessions *et al.* (*102*)	OR, reforestation in Siskiyou NF following severe fire	Average cost of most cost-effective reforestation approach in each of 4 post-burn years at two study locations	1145
Atkinson and Fitzgerald (*100*)	OR	Average of low and high cost data, 730 seedlings ha^{-1}	722

Table S5. Estimated marginal abatement cost of fire management by major forest region. It is assumed that leverage = 1**.**

**Forest region**	**MAC (USD per Mg CO_2e)**	**Tg CO_2e yr^{-1}**	**Cumulative Tg CO_2e yr^{-1}**
Black Hills	15.8	2.8	2.8
Southwest	19.5	3.2	6.0
Sierra Nevada	46.7	3.2	9.2
Cascades	57.9	2.5	11.7
Northern Rockies	129.1	4.3	16.0
Southern Rockies	3024.8	0.1	16.1

Table S6. Forest disturbance rates by source. Areas and rates of forest, forest disturbance by all disturbance types, anthropogenic clearing events (i.e., disturbance not due to fire or bark beetle), and forest conversions (i.e. cleared areas that did not return to forest within 10 years).

	**Area (Mha)**	**% per year**	**ha per year**
Area of forest in the NAFD product (*82*)	243.7	--	--
Area disturbed from 1986 to 2000	39.5	1.1%	2,630,056
Areas cleared from 1986 to 2000	35.5	1.0%	2,369,308
Area cleared from 1986 to 2000 that persisted as non-forest through 2010	5.7	0.16%	380,417

Table S7. Mean annual forest hectares cleared per year from 1986 to 2000.

Forest Type Group	NE	NLS	NPS	PNW	PSW	RMN	RMS	SC	SE
White/Red/Jack Pine Group	4670	13249	68	0	0	0	0	106	607
Spruce/Fir Group	26652	8705	0	0	0	0	0	0	0
Longleaf/Slash Pine Group	0	0	0	0	0	0	0	23745	89265
Loblolly/Shortleaf Pine Group	4447	0	84	0	0	0	0	340475	203125
Pinyon/Juniper Group	198	68	982	3669	4896	2991	62150	5145	59
Douglas-fir Group	2	0	3	112657	2289	60513	2753	0	0
Ponderosa Pine Group	0	0	4945	46417	8945	15505	16339	1	0
Western White Pine Group	0	0	0	18	25	6	0	0	0
Fir/Spruce/Mountain Hemlock Group	0	0	0	19075	3716	26656	11606	0	0
Lodgepole Pine Group	0	0	2	9846	1013	15611	8732	0	0
Hemlock/Sitka Spruce Group	0	0	0	14084	0	2224	0	0	0
Western Larch Group	0	0	0	352	0	821	0	0	0
Redwood Group	0	0	0	0	3508	0	0	0	0
Other Western Softwood Group	0	0	0	143	235	893	1822	0	0
California Mixed Conifer Group	0	0	0	908	27135	125	146	0	0
Exotic Softwoods Group	42	4	0	0	0	0	0	0	0
Oak/Pine Group	2187	91	1171	0	0	0	0	70221	46797
Oak/Hickory Group	65402	12738	79413	0	0	0	13	265803	156555
Oak/Gum/Cypress Group	330	0	172	0	0	0	0	70308	62031
Elm/Ash/Cottonwood Group	2163	2988	8923	790	0	941	240	27063	2313
Maple/Beech/Birch Group	87348	19639	9111	0	0	0	0	580	323
Aspen/Birch Group	2674	61152	653	75	38	2762	10245	59	0
Alder/Maple Group	0	0	0	13187	137	0	0	0	0
Western Oak Group	0	0	0	1600	39301	113	6484	1895	0
Tanoak/Laurel Group	0	0	0	730	5717	0	0	0	0
Other Western Hardwoods Group	0	0	0	750	680	350	5896	2535	0
Tropical Hardwoods Group	0	0	0	0	0	0	0	0	894
Exotic Hardwoods Group	0	0	0	0	169	0	0	1066	15

Table S8. Mean annual forest hectares cleared per year from 2001 to 2010.

Forest Type Group	NE	NLS	NPS	PNW	PSW	RMN	RMS	SC	SE
White/Red/Jack Pine Group	6899	15971	53	0	0	0	0	177	568
Spruce/Fir Group	28185	11197	1	0	0	0	0	0	0
Longleaf/Slash Pine Group	0	0	0	0	0	0	0	40526	140121
Loblolly/Shortleaf Pine Group	3946	0	96	0	0	0	0	491651	275870
Pinyon/Juniper Group	181	39	1604	3916	3674	2198	123239	7830	70
Douglas-fir Group	1	0	20	109945	1489	56645	8256	0	0
Ponderosa Pine Group	0	0	7106	38173	8524	15580	28402	2	0
Western White Pine Group	0	0	0	23	41	4	0	0	0
Fir/Spruce/Mountain Hemlock Group	0	0	0	11883	2944	21052	17392	0	0
Lodgepole Pine Group	0	0	3	5207	1146	10343	6137	0	0
Hemlock/Sitka Spruce Group	0	0	0	17066	0	2831	0	0	0
Western Larch Group	0	0	0	242	0	687	0	0	0
Redwood Group	0	0	0	0	2727	0	0	0	0
Other Western Softwood Group	0	0	0	198	331	559	3015	0	0
California Mixed Conifer Group	0	0	0	971	25938	172	205	0	0
Exotic Softwoods Group	40	3	0	0	0	0	0	0	0
Oak/Pine Group	2018	80	1162	0	0	0	0	87857	56833
Oak/Hickory Group	70606	10135	76882	0	0	0	15	319725	179412
Oak/Gum/Cypress Group	404	0	149	0	0	0	0	87133	77963
Elm/Ash/Cottonwood Group	2321	2545	9220	1085	0	2762	457	31369	2837
Maple/Beech/Birch Group	104232	18943	8562	0	0	0	0	575	295
Aspen/Birch Group	2706	60810	795	46	36	1742	15073	73	0
Alder/Maple Group	0	0	0	9990	92	0	0	0	0
Western Oak Group	0	0	0	1850	36395	476	14746	1976	0
Tanoak/Laurel Group	0	0	0	628	3639	0	0	0	0
Other Western Hardwoods Group	0	0	0	656	496	334	5151	4300	0
Tropical Hardwoods Group	0	0	0	0	0	0	0	0	1155
Exotic Hardwoods Group	0	0	0	0	276	0	0	1369	12

Table S9. Mean annual forest hectares converted per year from 1986 to 2000.

Forest Type Group	NE	NLS	NPS	PNW	PSW	RMN	RMS	SC	SE
White/Red/Jack Pine Group	802	1888	14	0	0	0	0	28	88
Spruce/Fir Group	620	572	0	0	0	0	0	0	0
Longleaf/Slash Pine Group	0	0	0	0	0	0	0	2955	13594
Loblolly/Shortleaf Pine Group	471	0	7	0	0	0	0	33176	19823
Pinyon/Juniper Group	41	5	146	934	566	510	18964	1042	5
Douglas-fir Group	0	0	1	19538	269	20032	842	0	0
Ponderosa Pine Group	0	0	1075	15948	1771	4690	4081	0	0
Western White Pine Group	0	0	0	11	8	1	0	0	0
Fir/Spruce/Mountain Hemlock Group	0	0	0	5922	1147	7381	3286	0	0
Lodgepole Pine Group	0	0	1	4779	267	5718	3424	0	0
Hemlock/Sitka Spruce Group	0	0	0	1460	0	545	0	0	0
Western Larch Group	0	0	0	97	0	245	0	0	0
Redwood Group	0	0	0	0	213	0	0	0	0
Other Western Softwood Group	0	0	0	24	23	218	464	0	0
California Mixed Conifer Group	0	0	0	307	5325	77	19	0	0
Exotic Softwoods Group	9	1	0	0	0	0	0	0	0
Oak/Pine Group	404	11	163	0	0	0	0	11174	7717
Oak/Hickory Group	10547	1017	12422	0	0	0	1	43276	29708
Oak/Gum/Cypress Group	53	0	10	0	0	0	0	7420	7050
Elm/Ash/Cottonwood Group	327	397	1244	250	0	220	72	5179	504
Maple/Beech/Birch Group	6144	1206	1969	0	0	0	0	209	20
Aspen/Birch Group	256	5867	102	30	3	322	2067	8	0
Alder/Maple Group	0	0	0	1745	19	0	0	0	0
Western Oak Group	0	0	0	511	8601	11	1462	414	0
Tanoak/Laurel Group	0	0	0	90	382	0	0	0	0
Other Western Hardwoods Group	0	0	0	133	304	57	2507	657	0
Tropical Hardwoods Group	0	0	0	0	0	0	0	0	403
Exotic Hardwoods Group	0	0	0	0	15	0	0	256	10

Table S10. Proportion of areas cleared from 1986 to 2000 that had not regenerated to forest by 2010.

Forest Type Group	NE	NLS	NPS	PNW	PSW	RMN	RMS	SC	SE
White/Red/Jack Pine Group	0.17	0.14	0.21	0.00	0.00	0.00	0.00	0.27	0.14
Spruce/Fir Group	0.02	0.07	0.07	0.00	0.00	0.00	0.27	0.00	0.00
Longleaf/Slash Pine Group	0.00	0.00	0.00	0.00	0.00	0.00	0.00	0.12	0.15
Loblolly/Shortleaf Pine Group	0.11	0.00	0.09	0.00	0.00	0.00	0.00	0.10	0.10
Pinyon/Juniper Group	0.21	0.07	0.15	0.25	0.12	0.17	0.31	0.20	0.09
Douglas-fir Group	0.01	0.00	0.31	0.17	0.12	0.33	0.31	0.00	0.00
Ponderosa Pine Group	0.00	0.07	0.22	0.34	0.20	0.30	0.25	0.01	0.00
Western White Pine Group	0.00	0.00	0.00	0.59	0.32	0.13	0.00	0.00	0.00
Fir/Spruce/Mountain Hemlock Group	0.00	0.00	0.00	0.31	0.31	0.28	0.28	0.00	0.00
Lodgepole Pine Group	0.00	0.00	0.30	0.49	0.26	0.37	0.39	0.00	0.00
Hemlock/Sitka Spruce Group	0.00	0.00	0.00	0.10	0.08	0.24	0.00	0.00	0.00
Western Larch Group	0.00	0.00	0.00	0.28	0.00	0.30	0.00	0.00	0.00
Redwood Group	0.00	0.00	0.00	0.00	0.06	0.00	0.00	0.00	0.00
Other Western Softwood Group	0.00	0.00	0.00	0.17	0.10	0.24	0.25	0.00	0.00
California Mixed Conifer Group	0.00	0.00	0.00	0.34	0.20	0.61	0.13	0.00	0.00
Exotic Softwoods Group	0.21	0.16	0.00	0.00	0.00	0.00	0.00	0.00	0.00
Oak/Pine Group	0.18	0.12	0.14	0.00	0.00	0.00	0.92	0.16	0.16
Oak/Hickory Group	0.16	0.08	0.16	0.00	0.00	0.00	0.11	0.16	0.19
Oak/Gum/Cypress Group	0.16	0.28	0.06	0.00	0.00	0.00	0.00	0.11	0.11
Elm/Ash/Cottonwood Group	0.15	0.13	0.14	0.32	0.00	0.23	0.30	0.19	0.22
Maple/Beech/Birch Group	0.07	0.06	0.22	0.00	0.00	0.00	0.00	0.36	0.06
Aspen/Birch Group	0.10	0.10	0.16	0.40	0.07	0.12	0.20	0.13	0.00
Alder/Maple Group	0.00	0.00	0.00	0.13	0.14	0.00	0.00	0.00	0.00
Western Oak Group	0.00	0.00	0.00	0.32	0.22	0.10	0.23	0.22	0.00
Tanoak/Laurel Group	0.00	0.00	0.00	0.12	0.07	0.00	0.00	0.00	0.00
Other Western Hardwoods Group	0.00	0.00	0.00	0.18	0.45	0.16	0.43	0.26	0.00
Tropical Hardwoods Group	0.00	0.00	0.00	0.00	0.00	0.00	0.00	0.00	0.45
Exotic Hardwoods Group	0.00	0.00	0.00	0.00	0.09	0.00	0.00	0.24	0.67

Table S11. Mean predisturbance dry biomass (kg m^{-2}) in forest areas converted from 1986 to 2000.

Forest Type Group	NE	NLS	NPS	PNW	PSW	RMN	RMS	SC	SE
White/Red/Jack Pine Group	14	7	14	0	0	0	0	13	13
Spruce/Fir Group	10	7	6	0	0	0	6	0	0
Longleaf/Slash Pine Group	0	0	0	0	0	0	0	8	8
Loblolly/Shortleaf Pine Group	11	0	11	0	0	0	0	9	10
Pinyon/Juniper Group	12	6	7	6	6	5	4	5	12
Douglas-fir Group	12	0	6	19	26	10	8	0	0
Ponderosa Pine Group	0	9	6	8	11	6	7	7	0
Western White Pine Group	0	0	0	17	17	13	0	0	0
Fir/Spruce/Mountain Hemlock Group	0	0	0	15	20	11	10	0	0
Lodgepole Pine Group	0	0	8	8	18	10	9	0	0
Hemlock/Sitka Spruce Group	0	0	0	23	21	14	0	0	0
Western Larch Group	0	0	0	11	0	10	0	0	0
Redwood Group	0	0	0	0	33	0	0	0	0
Other Western Softwood Group	0	0	0	15	15	8	7	0	0
California Mixed Conifer Group	0	0	0	9	19	5	13	0	0
Exotic Softwoods Group	13	9	0	0	0	0	0	0	0
Oak/Pine Group	12	7	9	0	0	0	6	7	10
Oak/Hickory Group	14	8	10	0	0	0	6	9	12
Oak/Gum/Cypress Group	11	11	10	0	0	0	0	9	9
Elm/Ash/Cottonwood Group	13	8	10	9	0	5	5	6	11
Maple/Beech/Birch Group	13	8	12	0	0	0	0	10	17
Aspen/Birch Group	12	8	11	10	14	7	8	6	0
Alder/Maple Group	0	0	0	19	23	0	0	0	0
Western Oak Group	0	0	0	13	10	5	6	4	0
Tanoak/Laurel Group	0	0	0	21	28	0	0	0	0
Other Western Hardwoods Group	0	0	0	20	6	7	5	4	0
Tropical Hardwoods Group	0	0	0	0	0	0	0	0	8
Exotic Hardwoods Group	0	0	0	0	11	0	0	6	7

Table S12. Mean predisturbance dry biomass (kg m^{-2}) in forest areas converted from 2001 to 2010.

Forest Type Group	NE	NLS	NPS	PNW	PSW	RMN	RMS	SC	SE
White/Red/Jack Pine Group	14	8	14	0	0	0	0	15	14
Spruce/Fir Group	11	8	6	0	0	5	6	0	14
Longleaf/Slash Pine Group	0	0	0	0	0	0	0	9	8
Loblolly/Shortleaf Pine Group	11	0	12	0	0	0	0	10	10
Pinyon/Juniper Group	12	7	6	6	5	5	4	4	12
Douglas-fir Group	13	0	6	23	27	10	8	0	0
Ponderosa Pine Group	0	8	7	9	11	6	7	9	0
Western White Pine Group	0	0	0	18	15	10	15	0	0
Fir/Spruce/Mountain Hemlock Group	0	0	0	14	18	11	10	0	0
Lodgepole Pine Group	0	0	8	9	16	9	9	0	0
Hemlock/Sitka Spruce Group	0	0	0	27	21	14	0	0	0
Western Larch Group	0	0	0	11	0	11	0	0	0
Redwood Group	0	0	0	0	34	0	0	0	0
Other Western Softwood Group	0	0	0	14	12	8	7	0	0
California Mixed Conifer Group	0	0	0	9	18	4	10	0	0
Exotic Softwoods Group	13	9	0	0	0	0	0	0	0
Oak/Pine Group	13	8	9	0	0	0	5	9	10
Oak/Hickory Group	14	9	10	0	0	7	6	10	12
Oak/Gum/Cypress Group	12	10	12	0	0	0	0	10	10
Elm/Ash/Cottonwood Group	12	8	9	8	0	5	4	8	13
Maple/Beech/Birch Group	13	9	12	0	0	0	6	11	17
Aspen/Birch Group	11	8	7	12	11	6	7	5	0
Alder/Maple Group	0	0	0	21	27	0	0	0	0
Western Oak Group	0	0	0	12	10	4	5	4	0
Tanoak/Laurel Group	0	0	0	21	28	0	0	0	0
Other Western Hardwoods Group	0	0	0	21	9	5	5	3	0
Tropical Hardwoods Group	0	0	0	0	0	0	0	0	8
Exotic Hardwoods Group	0	0	17	0	10	0	0	8	7

Table S13. Carbon emissions (Mg C year^{-1}) from estimated forest conversion from 2001 to 2010.

Forest Type Group	NE	NLS	NPS	PNW	PSW	RMN	RMS	SC	SE
White/Red/Jack Pine Group	52211	53792	472	0	0	0	0	2195	3610
Spruce/Fir Group	22725	18117	1	0	0	0	0	0	0
Longleaf/Slash Pine Group	0	0	0	0	0	0	0	140744	549807
Loblolly/Shortleaf Pine Group	14741	0	304	0	0	0	0	1435094	868798
Pinyon/Juniper Group	1417	63	4769	19032	7338	5688	452465	22166	226
Douglas-fir Group	1	0	126	1387391	14712	587091	66952	0	0
Ponderosa Pine Group	0	0	32905	360068	58743	94518	149199	0	0
Western White Pine Group	0	0	0	768	641	18	0	0	0
Fir/Spruce/Mountain Hemlock Group	0	0	0	166003	52547	205701	158826	0	0
Lodgepole Pine Group	0	0	19	70617	15135	111425	70721	0	0
Hemlock/Sitka Spruce Group	0	0	0	152676	0	31252	0	0	0
Western Larch Group	0	0	0	2297	0	6978	0	0	0
Redwood Group	0	0	0	0	17652	0	0	0	0
Other Western Softwood Group	0	0	0	1419	1262	3265	17553	0	0
California Mixed Conifer Group	0	0	0	9015	295218	1408	802	0	0
Exotic Softwoods Group	340	15	0	0	0	0	0	0	0
Oak/Pine Group	14685	237	4591	0	0	0	4	383466	302054
Oak/Hickory Group	506998	22143	373186	0	0	0	30	1602213	1298905
Oak/Gum/Cypress Group	2378	2	333	0	0	0	0	302177	292391
Elm/Ash/Cottonwood Group	13003	8886	35586	8728	0	10300	1741	155111	24562
Maple/Beech/Birch Group	308940	33460	70049	0	0	0	0	7191	1012
Aspen/Birch Group	9312	155598	2781	700	83	3967	68072	162	0
Alder/Maple Group	0	0	0	87751	1106	0	0	0	0
Western Oak Group	0	0	0	23058	251565	590	50946	4820	0
Tanoak/Laurel Group	0	0	0	5083	21449	0	0	0	0
Other Western Hardwoods Group	0	0	0	7699	6276	811	33316	11471	0
Tropical Hardwoods Group	0	0	0	0	0	0	0	0	12515
Exotic Hardwoods Group	0	0	0	0	752	0	0	8032	173

Table S14. Albedo-adjusted carbon emissions equivalent (Mg C_e $year^{-1}$) from estimated forest conversion from 2001 to 2010.

Forest Type Group	NE	NLS	NPS	PNW	PSW	RMN	RMS	SC	SE
White/Red/Jack Pine Group	26106	26896	236	0	0	0	0	1097	1805
Spruce/Fir Group	11362	9058	0	0	0	0	0	0	0
Longleaf/Slash Pine Group	0	0	0	0	0	0	0	70372	274904
Loblolly/Shortleaf Pine Group	7371	0	152	0	0	0	0	717547	434399
Pinyon/Juniper Group	709	31	2384	9516	3669	2844	226232	11083	113
Douglas-fir Group	0	0	63	693695	7356	293546	33476	0	0
Ponderosa Pine Group	0	0	16453	180034	29372	47259	74599	0	0
Western White Pine Group	0	0	0	384	321	9	0	0	0
Fir/Spruce/Mountain Hemlock Group	0	0	0	83002	26273	102851	79413	0	0
Lodgepole Pine Group	0	0	9	35308	7567	55713	35360	0	0
Hemlock/Sitka Spruce Group	0	0	0	76338	0	15626	0	0	0
Western Larch Group	0	0	0	1149	0	3489	0	0	0
Redwood Group	0	0	0	0	8826	0	0	0	0
Other Western Softwood Group	0	0	0	710	631	1633	8776	0	0
California Mixed Conifer Group	0	0	0	4508	147609	704	401	0	0
Exotic Softwoods Group	170	7	0	0	0	0	0	0	0
Oak/Pine Group	14685	237	4591	0	0	0	4	383466	302054
Oak/Hickory Group	506998	22143	373186	0	0	0	30	1602213	1298905
Oak/Gum/Cypress Group	2378	2	333	0	0	0	0	302177	292391
Elm/Ash/Cottonwood Group	13003	8886	35586	8728	0	10300	1741	155111	24562
Maple/Beech/Birch Group	308940	33460	70049	0	0	0	0	7191	1012
Aspen/Birch Group	9312	155598	2781	700	83	3967	68072	162	0
Alder/Maple Group	0	0	0	87751	1106	0	0	0	0
Western Oak Group	0	0	0	23058	251565	590	50946	4820	0
Tanoak/Laurel Group	0	0	0	5083	21449	0	0	0	0
Other Western Hardwoods Group	0	0	0	7699	6276	811	33316	11471	0
Tropical Hardwoods Group	0	0	0	0	0	0	0	0	12515
Exotic Hardwoods Group	0	0	0	0	752	0	0	8032	173

Table S15. Urban reforestation maximum potential annual net C sequestration in 2025.

Reforestation extent	Tg C yr^{-1} (95% CI)	Tg CO_2 yr^{-1} (95% CI)
95% CI Low	5.12 (4.28–6.48)	18.80 (15.72–23.79)
Mean	6.35 (5.22–8.21)	23.30 (19.17–30.15)
95% CI High	7.58 (6.16–9.95)	27.80 (22.62–36.51)

Table S16. Uncertainty in urban reforestation average annual abatement (Tg CO_2) by 2025 at a cost of USD 100 per Mg CO_2.

Reforestation extent	Low net C seq. rate	Mean net C seq. rate	High net C seq. rate
Low	0.1	0.2	0.4
Mean	0.1	0.2	0.4
High	0.1	0.3	0.5

Table S17. Profitability impacts of cover crops for selected crops.

Main crop	Profitability change vs. no cover crop, 2015USD ha^{-1}			Benefits analyzed
	Low	Mean	High	
No-till corn (*388*)	-0.8	55.8	127.9	Increased soil productivity
Corn (*389*)	-11.3	24.4	89.1	Reduced erosion, increased SOM, water holding capacity and nutrients (N)
Corn w/ stover removal (*389*)	-3.9	25.0	88.7	At the lower of their 2 assessed stover prices
No-till corn-soybean (*390*)	n/a	58.2	n/a	First rotation, short-term benefits only
No-till corn-soybean (*390*)	n/a	109.7	n/a	After first rotation, short-term benefits only
No-till corn-soybean (*390*)	n/a	128.7	n/a	After first rotation, including long-term benefits (increased soil fertility and water holding capacity)
Strip-till cotton (*391*)	34.6	143.3	318.6	Average of 4 different nitrogen application levels
No-till cotton (*218*)	n/a	-66.8	n/a	Long-term impacts (years 14 to 20 after plot establishment)
Conventional till cotton [a] (*218*)	n/a	-85.7	n/a	

a. For Cochran *et al.* (*218*), cost is calculated from the difference in profits ha^{-1} between the most profitable no cover crop and the most profitable cover crop option.

Table S18. Marginal abatement costs of cover crops in the five primary crops.

	Potential abatement Tg CO_2e yr^{-1}	Cost, USD per Mg CO_2e		
		Low	Mean	High
Corn	42.7	-86.8	-29.9	4.5
Soybean	37.9	-170.9	-88.6	8.6
Cotton–strip till	1.2	-271.6	-122.1	-29.5
Cotton–conventional till	2.0	n/a	73.0	n/a
Cotton–no-till	1.5	n/a	56.9	n/a
Rice	1.4	-179.2	-80.2	-12.5
Wheat	22.8	-179.2	-80.2	-12.5

Table S19. Maximum feasible N_2O reduction for multiple nitrogen fertilizer practices.

Practice	Reduction (ratio for treated ha)	Applicable area[a] (fraction of total)	Proportion remaining after reduction (ratio)	Calculate new emission (cumulative, multiplicative)[b] (ratio)	Source of reduction value
Reduce rate	0.21	0.64	0.87	0.87	Sela *et al.* (*265*)
Switch from anhydrous to urea	0.29	0.35	0.90	0.78	Eagle *et al.* (*6*)
Improve timing	0.09	0.50	0.96	0.74	Eagle *et al.* (*6*)
Use variable rate within field	0.15	0.64	0.90	0.67	Our estimate (see table S20).
**New Emission (Fraction of total BAU emission)**				**0.67**	

a. Applicable area estimates derived from (*6*).

b. Effects of each practice are multiplied together to calculate the total new emission.

Table S20. Results from the literature of the potential for reducing N fertilizer rate using within-field management.

Reference	Crop	Region	Comments	Site-years	Reduction	Reduction units	Reduction (ratio over control treatment)
Sehy *et al.* (*279*)	Maize	Germany (near Munich)	Within-field treatment included 25 kg N ha^{-1} more on 66% of field with higher yields and 25 kg N ha^{-1} less on 34% of lower yielding portion of field. Includes freeze-thaw period.	1	0.41	kg N_2O-N ha^{-1}	0.12
Brandes *et al.* (*37*)	Maize, Soybean	State of Iowa	Model analysis of entire state at sub-field scale over multiple years to determine portion of crop area that incurs a loss of greater than or equal to USD 250 ha^{-1}	4 years, all fields in IA	0.27	portion of cropland area with negative profit.	0.27
Roberts *et al.* (*392*)	Maize	USA: MO	Across a range of soil types, N sensor-based applications and use of adjacent N-rich reference strips could reduce N rate by 10–50 kg N ha^{-1}.	16	0.16	N Rate	0.16
Scharf *et al.* (*397*), Hong *et al.* (*394*).	Maize	USA: MO	Half of each field is over or under-fertilized by 34 kg N, based on calculations of EONR for 20x40 m regions within each field. This variation is similar for the upper and lower quartiles, therefore 25% of each field is overfertilized by 34 kg N (*393*).	8	0.06	N rate	0.06
**AVERAGE**							**0.15**

Table S21. Current and projected GHG emissions from nitrogen fertilizer manufacturing in the United States.

**Fertilizer type**	**Average upstream emissions**[a] **(kg-CO_2e kg-N^{-1})**	**Current N fertilizer use**[b] **(% of total)**	**Projected BAU N fertilizer use in 2025 (% of total)**	**Projected BMP N fertilizer use in 2025 (% of total)**
Anhydrous ammonia	2.60	31%	31%	0%
Aqueous ammonia	2.60	0%	0%	0%
Ammonium nitrate	9.70	3%	3%	3%
Urea	3.20	27%	27%	58%
Urea-ammonium nitrate	6.86	31%	31%	31%
Ammonium sulfate		3%	3%	3%
Other		5%	5%	5%
Sum		**100%**	**100%**	**100%**
Average upstream emission factor (g-CO_2e g-N^{-1})	**4.41**	**4.41**	**4.41**	**4.62**
Total N fertilizer use (Tg N yr^{-1})		**11.86**	**12.41**	**9.66**
Total upstream GHG emissions (Tg CO_2e yr^{-1})		**52.37**	**54.78**	**44.62**

a. Source: Snyder *et al.* (*395*)

b. Source: AAPFCO 2014 (*396*)

Table S22. Mitigation potential for grazing optimization and legumes in pasture NCS at different marginal abatement costs.

Carbon price (USD Mg CO_2e^{-1})	Grazing optimization (Tg CO_2e)	Legumes interseeding (Tg CO_2e)
0	5.31	2.50
10	6.09	3.36
20	6.90	4.40
50	7.77	5.44
100	8.51	5.62
Maximum	10.60	7.03

Table S23. Areas and carbon fluxes for Histosols in the conterminous United States. Negative values indicate net fluxes to the atmosphere.

IPCC Climate Zone[a,b]	Undisturbed Histosols [b]	Crop	Pasture	Other disturbed	Net CO_2 flux	Net CH_4 flux	Total flux
	Thousands of ha				Mg CO_2e yr^{-1} ha^{-1}		
Tropical Moist	15,943	2,100[b]	1,100[b]	3,365[b]	7.04	-2.51	4.52
VA,NC,SC	6,151	---------- 448[b] -------		749[b]	5.67	-0.21	5.46
Warm Temp.	15,099	663[c]	589[c]	414[b]	11.09	-2.89	8.20
Cool Temp.	66,258	3,900[c]	3,200[c]	2,967[b]	5.61	-2.14	3.47
**Total**	103,451	7,111	4,889	7,495	NA	NA	NA

a. Tropical Moist = FL; Warm Temperate = AL, AR, AZ, CA, DE, GA, IA, IL, IN, KS, KY, LA, MD, MO, MS, NJ, OK, TN, TX; Cool Temperate = CO, CT, ID, MA, ME, MI, MN, MT, ND, NE, NH, NM, NV, NY, OH, OR, PA, RI, SD, UT, VT, WA, WI, WV, WY.
b. See main text.
c. US EPA (*309*).

Table S24. Peatland restoration mitigation calculations for climate zones within the United States.

	BAU emissions			Benefit from restoring:				CH4 emissions from restoring:				
IPCC Climate Zone[a,b]	Crop & Pasture soils	Horti-culture soils	Other Soils	Crop Soils	Pasture Soils	Bio-mass	DOM export reduc-tion	Crop	Pasture	Hort.	Other	Total
	Tg C yr^{-1}							Tg CO_2e				
Tropical Moist	-3.33[c,d]	-0.09[c,g]	-0.87[f]	0.00[h]	0.00[h]	0.25[k,l]	0.08[m]	-0.52[n]	-0.27[n]	-0.02[g,u]	-0.84[r]	2.97
VA,NC,SC	-0.20[e]	-0.03[c,g]	-0.25[e]	0.03[e]	0.04[e]	0.12[e]	0.01[e]	-----0.01[e] -----		0.00[e]	-0.02[e]	0.00
Warm Temp.	-1.60[c,d]	-0.08[c,g]	-0.11[f]	-0.03[i]	0.02[j]	0.05[k,l]	0.02[m]	-0.23[o]	-0.13[p]	-0.02[g,v]	-0.09[s]	1.37
Cool Temp.	-4.46[c,d]	-0.02[c,g]	-0.77[f]	-0.20[i]	0.14[j]	0.32[k,l]	0.12[m]	-1.38[o]	-0.38[q]	- 0.04[g,w]	-0.35[t]	3.50
**Total**	-9.58	-0.23	-2.00	-0.20	0.21	0.74	0.23	-2.14	-0.79	-0.08	-1.30	8.49

a. Tropical Moist = FL; Warm Temperate = AL, AR, AZ, CA, DE, GA, IA, IL, IN, KS, KY, LA, MD, MO, MS, NJ, OK, TN, TX; Cool Temperate = CO, CT, ID, MA, ME, MI, MN, MT, ND, NE, NH, NM, NV, NY, OH, OR, PA, RI, SD, UT, VT, WA, WI, WV, WY.
b. See main text.
c. US EPA (*309*).
d. Does not include soil C change associated with federal croplands, CRP enrollment after 2010, or sewage sludge application to soils.
e. Richardson *et al.* (*318*).
f. Using an emission factor of -2.6 Mg CO_2 ha^{-1} yr^{-1} for drained, forested, temperate Histosols (*305*).
g. Values were apportioned to the different climate zones by the proportion of peat extracted from nutrient-rich and nutrient-poor peatlands, with the former primarily from southern states (i.e., tropical moist and warm temperate zones) and the latter from northern Sphagnum bogs (i.e., cool temperate zone) (*309*).

h. Using an emission factor of 0.00 Mg CO_2 ha^{-1} yr^{-1} for tropical rewetted Histosols (*305*).
i. Using an emission factor of -0.50 Mg CO_2 ha^{-1} yr^{-1} for temperate rich rewetted Histosols (*305*).
j. Using an emission factor of 0.23 Mg CO_2 ha^{-1} yr^{-1} for temperate poor rewetted Histosols (*305*).
k. Using an annual live biomass increment of 50 g m^{-2} yr^{-1} (*308*).
l. Assuming all Other Disturbed Histosols will be restored to forests and the proportion of crop and pasture areas restored to forests will reflect the current distribution of forested peatlands.
m. Using a decrease in dissolved organic carbon flux upon rewetting of 12.2 g C m^{-2} yr^{-1} (*305*).
n. Using geometric mean CH_4 flux from 12 studies in mostly natural wetlands in the U.S. [-156 Kg CH_4 ha^{-1} yr^{-1}; (*308*), updated] minus flux from drained, tropical and sub-tropical, cropland and grassland Histosols [-5.3 Kg CH_4 ha^{-1} yr^{-1}, (*305*)].
o. Using an emission factor of -216 kg CH_4 ha^{-1} yr^{-1} for temperate, rich, rewetted Histosols (*305*) minus flux from drained, boreal and temperate, rich Histosols [0 Kg CH_4 ha^{-1} yr^{-1}, (*305*)].
p. Using geometric mean CH_4 flux from 14 studies in natural wetlands in the U.S. [-141 Kg CH_4 ha^{-1} yr^{-1}; (*308*), updated] minus flux from drained, temperate, nutrient-poor, grassland Histosols [-1.4 Kg CH_4 ha^{-1} yr^{-1}, (*305*)].
q. Using geometric mean CH_4 flux from 53 studies in natural wetlands in the U.S. [-74 Kg CH_4 ha^{-1} yr^{-1}; (*308*), updated] minus flux from drained, temperate, nutrient-poor, grassland Histosols [-1.4 Kg CH_4 ha^{-1} yr^{-1}, (*305*)].
r. Using geometric mean CH_4 flux from 12 studies in mostly natural wetlands in the U.S. [-156 Kg CH_4 ha^{-1} yr^{-1}; (*308*), updated] minus flux from drained, tropical and sub-tropical, forested Histosols [-3.7 Kg CH_4 ha^{-1} yr^{-1}, (*305*)].
s. Using geometric mean CH_4 flux from 14 studies in natural wetlands in the U.S. [-141 Kg CH_4 ha^{-1} yr^{-1}; (*308*), updated] minus flux from drained, temperate, forested Histosols [-1.9 Kg CH_4 ha^{-1} yr^{-1}, (*305*)].
t. Using geometric mean CH_4 flux from 53 studies in natural wetlands in the U.S. [-74 Kg CH_4 ha^{-1} yr^{-1}; (*308*), updated] minus flux from drained, temperate, forested Histosols [-1.9 Kg CH_4 ha^{-1} yr^{-1}, (*305*)].
u. Using geometric mean CH_4 flux from 12 studies in mostly natural wetlands in the U.S. [-156 Kg CH_4 ha^{-1} yr^{-1}; (*308*), updated] minus flux from peat extraction sites [-4.6 Kg CH_4 ha^{-1} yr^{-1}, (*305*)].
v. Using geometric mean CH_4 flux from 14 studies in natural wetlands in the U.S. [-141 Kg CH_4 ha^{-1} yr^{-1}; (*308*), updated] minus flux from peat extraction sites [-4.6 Kg CH_4 ha^{-1} yr^{-1}, (*305*)].
w. Using geometric mean CH_4 flux from 53 studies in natural wetlands in the U.S. [-74 Kg CH_4 ha^{-1} yr^{-1}; (*308*), updated] minus flux from peat extraction sites [-4.6 Kg CH_4 ha^{-1} yr^{-1}, (*305*)].

Table S25. 95% CIs for Histosol calculations.

	Mean Tg C yr^{-1}	lower bound %	upper bound %
Soil C Loss from Crop, Pasture[a]	-9.58	43	33
Soil C Loss from Other Disturbed[b]	-2.00	23	23
Oxidation due to Horticulture[c]	-0.20	19	19
Change in Soil C from Crop Restored[b]	-0.23	342	242
Change in Soil C from Pasture, Disturbed Restored[b]	0.21	178	278
Live Biomass Accumulation Restored[d]	0.74	25	25
Reduction in DOM Export Restored[b]	0.23	50	50
Change in CH_4 Emissions Due to Restored Crop[e]	-2.14	22	22
Change in CH_4 Emissions Due to Restored Pasture[e]	-0.79	22	22
Change in CH_4 Emissions Due to Restored Other Disturbed[e]	-1.30	22	22
Change in CH_4 Emissions Due to Restored Horticulture[e]	-0.03	22	22
0verall[f]		46	29

a. From USDA (*397*) for just agriculture.
b. From IPCC (*305*) for emission factor, but does not include error for area.
c. From EPA (*309*).
d. Brown *et al.* (*398*) gave only a 2% sampling error for wetland forest growth in the South Atlantic States, but this was increased to 25% to account for wetland forests on mineral soil in the South Atlantic States and the majority of U.S. Histosols are elsewhere.
e. Using the variation of the geometric mean of CH_4 emissions for all U.S. Histosols [update of (*308*)].
f. The uncertainties were combined by their weighted sums (*72*).
